# A Photonastic Prototissue Capable of Photo‐Mechano‐Chemical Transduction

**DOI:** 10.1002/adma.202502830

**Published:** 2025-05-12

**Authors:** Agostino Galanti, Beatrice Rosetti, Stefano Valente, Nicoletta Braidotti, Maria Sbacchi, Silvia Todros, Piero Pavan, Pierangelo Gobbo

**Affiliations:** ^1^ Department of Chemical and Pharmaceutical Sciences Università degli Studi di Trieste Via L. Giorgieri, 1 Trieste 34127 Italy; ^2^ Department of Industrial Engineering Università degli Studi di Padova Padova 35131 Italy; ^3^ Tissue Engineering Lab Fondazione Istituto di Ricerca Pediatrica Città della Speranza Corso Stati Uniti, 4F Padova 35127 Italy; ^4^ National Interuniversity Consortium of Materials Science and Technology Unit of Trieste Via G. Giusti 9 Firenze 50121 Italy

**Keywords:** energy transduction, far‐from‐equilibrium material, photonastic behaviour, protocell, prototissue

## Abstract

Despite recent significant advances in the controlled assembly of protocell units into complex 3D architectures, the development of prototissues capable of mimicking the sophisticated energy transduction processes fundamental to living tissues remains a critical unmet challenge in bottom‐up synthetic biology. Here a synthetic approach is described to start addressing this challenge and report the bottom‐up chemical construction of a photonastic prototissue endowed with photo‐mechano‐chemical transduction capabilities. For this, novel protocells enclosing photothermal transducing proto‐organelles based on gold nanoparticles and a thermoresponsive polymeric proto‐cortex are developed. These advanced protocell units are assembled into prototissues capable of light‐induced reversible contractions and complex motions, which can be exploited to reversibly switch off a coordinated internalized enzyme metabolism by blocking the access of small substrate molecules. Overall, the work provides a synthetic pathway to constructing prototissues with sophisticated energy transduction mechanisms, enabling the rational design of emergent behaviors in synthetic materials and addressing critical challenges in bottom‐up synthetic biology and bioinspired materials engineering.

## Graphical abstract

Mimicking energy transduction in prototissue assemblies remains a challenge of bottom‐up synthetic biology. In this work, prototissues integrating protocells with photothermal gold nanoparticle proto‐organelles and a thermoresponsive polymeric proto‐cortex are developed. These prototissues exhibited light‐controlled reversible contractions, programmable motions, and dynamic control of enzymatic metabolism by restricting substrate access, pioneering a route toward bioinspired materials with emergent energy transduction behaviors.

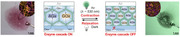

## 1 Introduction

All living tissues exhibit higher‐order behaviors that allow the cells that compose them to respond collectively and rapidly to changes in their environment through the use of a range of adaptive mechanisms. At the core of these higher‐order behaviors and adaptive nature of living tissues are hierarchical signaling cascades that enable the cellular building units to transform a stimulus signal into a variety of outputs. These functions are made possible by sophisticated receptors that are capable of transducing a range of energy types, including luminous, chemical, mechanical, and other forms. These energy interconversions in biological systems usually occur at the nanoscale thanks to transmembrane proteins (e.g., L‐type calcium channels, G‐protein‐coupled receptors) and cascades of biological events that can lead to microscale (e.g., sarcomere contraction, auxin‐promoted cell expansion) and macroscale events (e.g., muscle contractility, circumnutation of plant stems).

Mimicking such sophisticated biological energy transduction processes in synthetic materials represents one of the greatest scientific challenges of our time. As Nature taught us, addressing this challenge requires the chemical design and synthesis of specialized systems for energy transduction, the integration of such systems into materials capable of self‐assembly, and synergistic feedback between the chemical properties of the enclosed energy transduction systems and the mechanical properties of the ensemble.

Nature also showed us that the key to fabricating the next generation of materials capable of energy transduction and transformation (also termed “active matter”)^[^ ^1^ ^]^ relies on the hierarchical ordering of molecular building blocks and cellular units through compartmentalization. Only through the coordinated activities and the emergent responses of the constituent parts higher‐order behaviors such as the steadfast coordinated contraction of the heart or the marvelous photonastic opening and closure of a flower can be achieved in synthetic materials.

Inspired by these observations, herein we report the bottom‐up chemical construction of photonastic^[^ ^2^ ^]^ tissue‐like materials, termed “prototissues” or “protocellular materials” (PCMs), endowed with a photo‐mechano‐chemical transduction mechanism. In these advanced forms of biomimetic materials, a light‐induced mechanical movement can influence an internalized enzyme metabolism hosted within the constituent units of the material itself.

Prototissues (or PCMs) are synthetic tissue‐like materials formed from the assembly of protocell units. These materials are free‐standing, have dimensions ranging between hundreds of microns and a few centimeters in size, and are capable of performing complex functions, including long‐range communication, macroscopic deformation, signal propagation, and enhanced chemical gradient sensing.^[^ ^3^, ^4^, ^5^, ^6^, ^7^, ^8^, ^9^ ^]^ Protocells are broadly defined as highly simplified models of natural cells assembled from the bottom up from natural or synthetic components.^[^ ^10^ ^]^ A range of different protocells have been developed so far based on lipid vesicles,^[^ ^11^, ^12^, ^13^ ^]^ polymersomes,^[^ ^14^, ^15^ ^]^ polypeptide capsules,^[^ ^16^, ^17^ ^]^ dendrimersomes,^[^ ^18^ ^]^ colloidosomes,^[^ ^19^ ^]^ and coacervate microdroplets.^[^ ^20^, ^21^ ^]^ Protocells are engineered to display life‐like functionalities including enzymatic metabolism, energy generation, motility, communication, division, and evolution.^[^ ^22^ ^]^ Among the different protocell models, proteinosomes are gaining increasing interest. They are a fully organic type of colloidosome whose membrane is composed of a monolayer of self‐assembled protein‐polymer nanoparticles. One of the most interesting features of proteinosomes is their membrane semi‐permeability, being characterized by a molecular weight cut‐off (MWCO). Proteinosomes are also biocompatible, and their membrane can be engineered to display an intrinsic enzymatic activity.^[^ ^23^, ^24^, ^25^, ^26^ ^]^

To date, research on prototissues focuses on developing methodologies to assemble protocell units into increasingly precise 3D architectures and on the interaction between prototissues and living cells and tissues.^[^ ^27^ ^]^ For example, in our group, we developed a method that allows to covalently bind protocells together using bio‐orthogonal chemistry, and specifically the interfacial strain‐promoted alkyne‐azide cycloaddition (I‐SPAAC) reaction.^[^ ^7^ ^]^ We further refined this approach through a “floating mold technique” to fabricate free‐standing, millimeter‐sized prototissues (PCMs) with controlled 3D architectures.^[^ ^8^ ^]^ This technique involves floating a laser‐cut, 0.5 mm thick poly(tetrafluoroethylene) (PTFE) sheet on an aqueous polysorbate 80 solution (5 wt.%) to contain a 1:1 mixture of azide‐ and strained alkyne‐functionalized proteinosomes in oil. The PTFE mold holds in place the emulsion, while the surfactant and Marangoni flow (generated by surface tension gradients between the aqueous and oil phases) progressively remove the oil. This brings the bio‐orthogonally reactive proteinosomes into contact, allowing them to form covalent bonds and create the prototissue structure. Networks of interconnected protocells with complex geometries and behaviors were also fabricated by 3D printing of lipid‐based protocells with single protocell resolution,^[^ ^4^ ^]^ or by using the magneto‐Archimedes effect allowing to assemble giant unilamellar vesicles into prototissues capable of interfac with living tissues.^[^ ^5^ ^]^ A type of prototissue was recently made by embedding protocells into a gelatin matrix. Despite the fact that this material features no direct protocell‐protocell adhesions (engineering protocell‐protocell adhesions is currently one of the major challenges in prototissue engineering), the authors showed that this material is capable of responding to various external stimuli (i.e., osmosis, temperature, light) and of two‐way chemical communication in prototissue pairs.^[^ ^28^ ^]^ In addition, our group, in collaboration with Prof. T. Banno, has recently developed a method to extrude free‐standing and modular prototissue fibers of controlled length, diameter, and shape by exploiting interfacial salt bridges between vesicle units.^[^ ^29^ ^]^ Despite these important advancements, the development of synthetic prototissues endowed with higher‐order behaviors to date remains essentially unexplored.

Inspired by the higher‐order behaviors of living tissues, in this work, we present the bottom‐up chemical construction of photonastic prototissues capable of photo‐mechano‐chemical transduction. We achieved this first by developing novel bio‐orthogonally reactive proteinosomes enclosing in their lumen poly(ethylene glycol)‐stabilized 14 nm gold nanoparticles (PEG‐AuNPs) as photothermal transducing proto‐organelles, and endowed with synthetic thermoresponsive poly(*N*‐isopropylacrylamide) (PNIPAM)‐based proto‐cortex. Inspired by the cortex of living cells, our synthetic PNIPAM‐based proto‐cortex was a polymeric network layer that underlay the entire protocell membrane.^[^ ^6^, ^30^, ^31^ ^]^ The synthetic proto‐cortex, similarly to its natural counterpart, provided mechanical robustness to the microcompartment and generated forces that allowed the entire protocell to modulate its shape and mechanical properties. These advanced proteinosomes were assembled into photonastic prototissues of different sizes and shapes using the floating mold technique. Upon 520 nm light irradiation, the PEG‐AuNPs proto‐organelles contained in the protocell lumen generated localized heat through the photothermal effect, causing the PNIPAM‐based thermoresponsive proto‐cortex to contract and triggering fast and reversible contractions of the entire prototissue. This photoresponsive behavior was then exploited to fabricate a prototissue with the shape of a six‐armed starfish capable of closing like a flower upon irradiation. This prototissue comprised a top layer of photo‐contractile protocells and a bottom layer of non‐photo‐contractile protocells. The structural continuity between the two layers created via I‐SPAAC reaction allowed light irradiation to trigger a contraction in the photoresponsive layer, which induced a strain field on the underlying non‐responsive layer, causing the entire structure to bend as it maintained self‐equilibrium. Importantly, we also demonstrated that the photonastic behavior could reversibly switch off the permeability of the protocell membranes that compose the material. We exploited this property to fabricate a prototissue that exhibited a photo‐mechano‐chemical transduction behavior, where the system remained inert during continuous light irradiation but activated an internalized protocell‐coordinated enzyme cascade when the light was switched off. This sophisticated energy transduction mechanism relied on the properties of the PNIPAM‐based proto‐cortex, which contracted and expelled water when heated by the AuNPs photothermal effect. This contraction made the polymer network hydrophobic and decreased its mesh size, preventing hydrophilic substrate molecules from permeating the membrane to reach the enzymes in the protocell lumen. Both the photonastic and the photo‐mechano‐chemical transduction higher‐order behaviors emerged from the collective interactions and coordinated responses that were enabled by the spatial integration of the protocell units within the prototissue.

## 2 Results and Discussion

### 2.1 Bottom‐Up Assembly of Photonastic Prototissues

**Figure 1:**
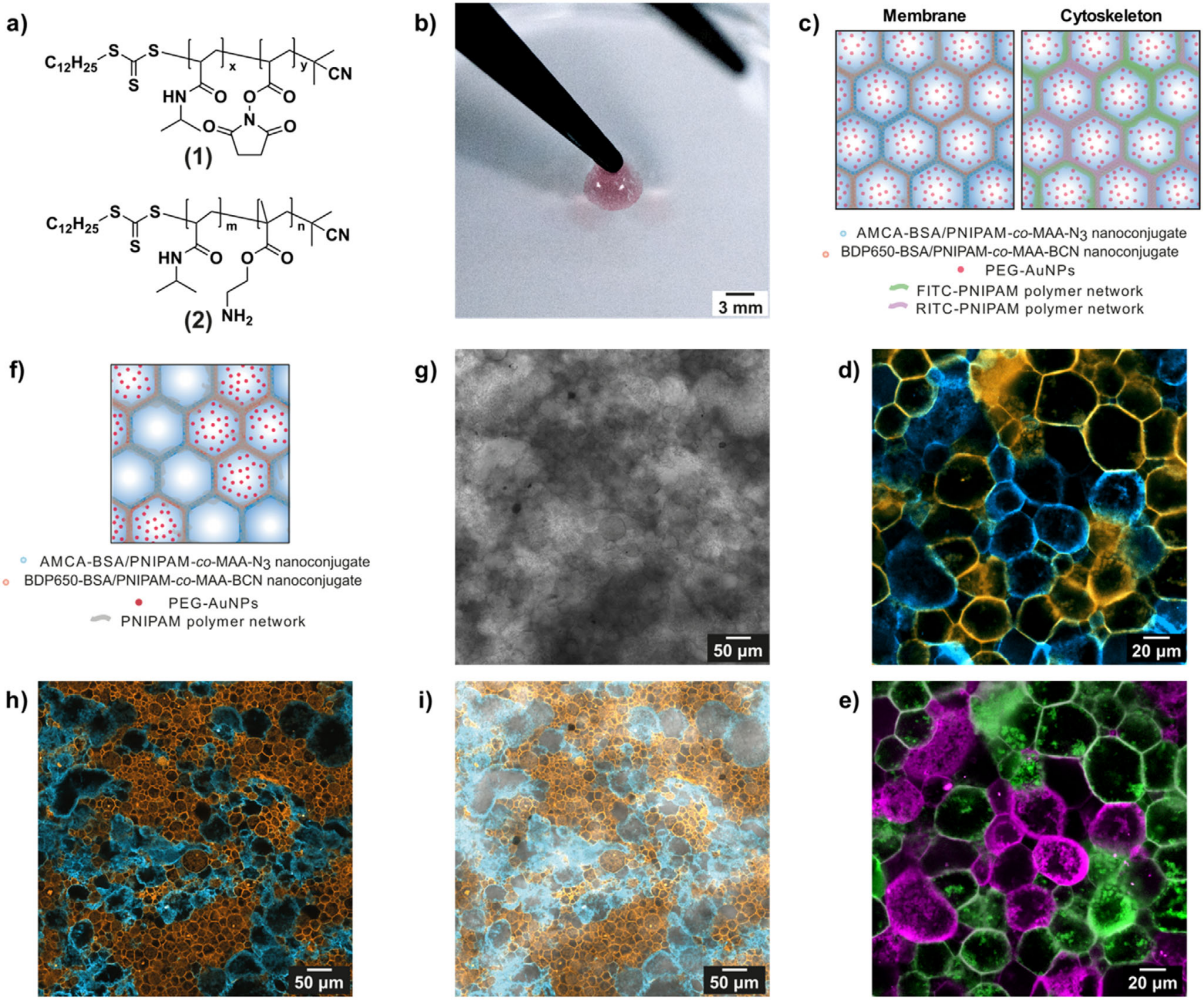
Structural characterization of photonastic prototissues. a) Structures of the PNIPAM‐based copolymers (1) and (2) used to build the proteinosome proto‐cortex. b) Photograph of a prototissue composed of protocells endowed with a PNIPAM proto‐cortex and encapsulating PEG‐AuNPs showing the typical pink coloration originating from the gold colloid. c) Scheme showing the structure of a prototissue, which comprises interconnected protocells composed of a membrane of AMCA‐labeled azide‐functionalized BSA/PNIPAM‐*co*‐MAA nanoconjugate and of a RITC‐tagged proto‐cortex, or composed of a membrane of BDP650‐labeled BCN‐functionalized BSA/PNIPAM‐*co*‐MAA nanoconjugate and of an FITC‐tagged proto‐cortex. Both types of protocells enclose PEG‐AuNPs. The left scheme highlights the membrane composition, whereas the right scheme highlights the proto‐cortex architecture and composition. d,e) Confocal fluorescence microscopy images showing the inner structure of a prototissue structured like in (c), highlighting the interconnected protocell membranes (azide‐functionalized AMCA‐labeled BSA/PNIPAM‐*co*‐MAA nanoconjugate – blue fluorescence, and BCN‐functionalized BDP650‐labeled BSA/PNIPAM‐*co*‐MAA nanoconjugate – orange fluorescence) d), or the two differently tagged protocell proto‐cortexes (FITC‐labeled proto‐cortex—green fluorescence, and RITC‐labeled proto‐cortex—purple fluorescence) e). f) Scheme showing the structure of a prototissue which comprises interconnected protocells composed of a membrane of AMCA‐ or BDP650‐labeled azide‐ or BCN‐functionalized BSA/PNIPAM‐*co*‐MAA nanoconjugate and of a non‐fluorescently tagged proto‐cortex. Only the BDP650‐labeled protocells encapsulate PEG‐AuNPs. g) Brightfield microscopy image of the prototissue in (f). Dark areas highlight the presence of PEG‐AuNPs. h) Confocal fluorescence microscopy image of the prototissue in (f), highlighting the interconnected structure of protocell membranes (azide‐functionalized AMCA‐labeled BSA/PNIPAM‐*co*‐MAA nanoconjugate – blue fluorescence, and BCN‐functionalized BDP650‐labeled BSA/PNIPAM‐*co*‐MAA nanoconjugate – orange fluorescence). i) Merged microscopy image of channels shown in (g) and (h). The correspondence of dark areas in (g) with the orange areas in (h) indicates that the PEG‐AuNPs were specifically localized inside the BDP650‐labeled protocells.

Photonastic prototissues were assembled using the floating mold technique^[^ ^8^ ^]^ from a 1:1 binary population of bioinspired azide‐ and bicyclononyne (BCN)‐functionalized proteinosomes endowed with a PNIPAM‐based synthetic polymeric proto‐cortex and PEG‐AuNPs as proto‐organelles capable of transducing light into thermal energy. These proteinosomes were fabricated using the Pickering emulsion technique, first by mixing fluorescently labeled azide‐ or BCN‐functionalized bovine serum albumin (BSA)/PNIPAM‐*co*‐methacrylic acid (MAA) nanoconjugates (Section S2.1, Supporting Information), copolymer (**1**), copolymer (**2**) (**Figure** **1**a; Sections S2.2 and S2.3, Supporting Information), and PEG‐AuNPs (Section S2.8, Supporting Information) in Na_2_CO_3_ buffer (pH 8.5, 100 mm), and then by emulsification with 2‐ethyl‐1‐hexanol (Section S3, Supporting Information). Within the so‐formed BSA/PNIPAM‐*co*‐MAA nanoconjugate‐stabilized water‐in‐oil microdroplets, copolymer (**1**) reacted with copolymer (**2**) to generate an internal polymer network, and also reacted with the residual interfacial amines of the BSA/PNIPAM‐*co*‐MAA nanoconjugate to crosslink the proteinosome membrane. The hydrophilic and non‐reactive PEG‐AuNPs remained dissolved in the aqueous phase and enclosed inside the chemically crosslinked proteinosome membrane. The resulting proteinosomes in oil showed irregular shapes, with lower sphericity compared to “empty” azide‐ or BCN‐functionalized proteinosomes in oil.^[^ ^7^ ^]^ They had a mean equivalent diameter of 17 ± 7 µm (mean volume ≈ 3 pL) and a roundness of 0.75 ± 0.15 (Figure S32, Supporting Information). The protein‐polymer nanoconjugate formed a protocell membrane that had an irregular thickness of ≈3 ± 1 µm, whereas the PNIPAM‐based polymer network and the PEG‐AuNPs were composing the protocell protocytoplasm.

Circular photonastic prototissues 5 mm in diameter were assembled from a 1:1 binary population of this azide‐ and BCN‐functionalized proteinosome units using the floating mold technique. For this, an emulsion of bio‐orthogonally reactive proteinosomes was drop‐cast into a circular 5 mm in diameter PTFE mold and left overnight, allowing the simultaneous assembly of the prototissue with the desired size and shape and its transfer to bulk water phase (Sections S4.1, Supporting Information). Figure 1b shows an image of a freshly formed PCM, which looks red due to the presence of the enclosed PEG‐AuNPs. The presence of PEG‐AuNPs inside the material was confirmed by UV–vis spectroscopy analysis carried out on the prototissue, which showed the presence of the typical plasmon resonance band at 527 nm (Figure S33, Supporting Information). Despite the irregular morphology of the protocell units in oil, confocal fluorescence microscopy (Figure 1c–e) showed proper formation of PCMs, with results comparable to what previously reported by our group.^[^ ^8^ ^]^ The protocell units were fully interconnected and showed thin and well‐defined protein‐polymer nanoconjugate membranes. Interestingly, upon transfer to water, most of the PNIPAM‐based polymer network adhered to the inner part of the protocell membrane, forming a 2 ± 1 µm thick cortex‐like structure and leaving an internal lumen (Figure 1e). Importantly, the BSA/PNIPAM‐*co*‐MAA proteinosome membrane, which had a molecular weight cut‐off (MWCO) of 25 kDa (vide infra), effectively prevented the leakage of high molecular weight copolymers (1) and (2) (*M_n_* of 125.2 ± 0.8 × 10^3^ g mol^−1^ and 65.3 ± 0.6 × 10^3^ g mol^−1^, respectively) and PEG‐AuNPs. In fact, by fluorescently tagging copolymer (2) either with fluorescein isothiocyanate or rhodamine B isothiocyanate (Sections S2.3, Supporting Information), confocal microscopy confirmed the segregation of differently tagged proto‐cortexes within distinct proteinosome populations (Figure 1c–e). To verify PEG‐AuNPs confinement, a prototissue was created using a 1:1 binary emulsion of proteinosomes, with only one population containing PEG‐AuNPs. Merged confocal fluorescence and brightfield microscopy imaging confirmed that PEG‐AuNPs remained within their initially encapsulating proteinosome population (Figure 1f–i).

**Figure 2:**
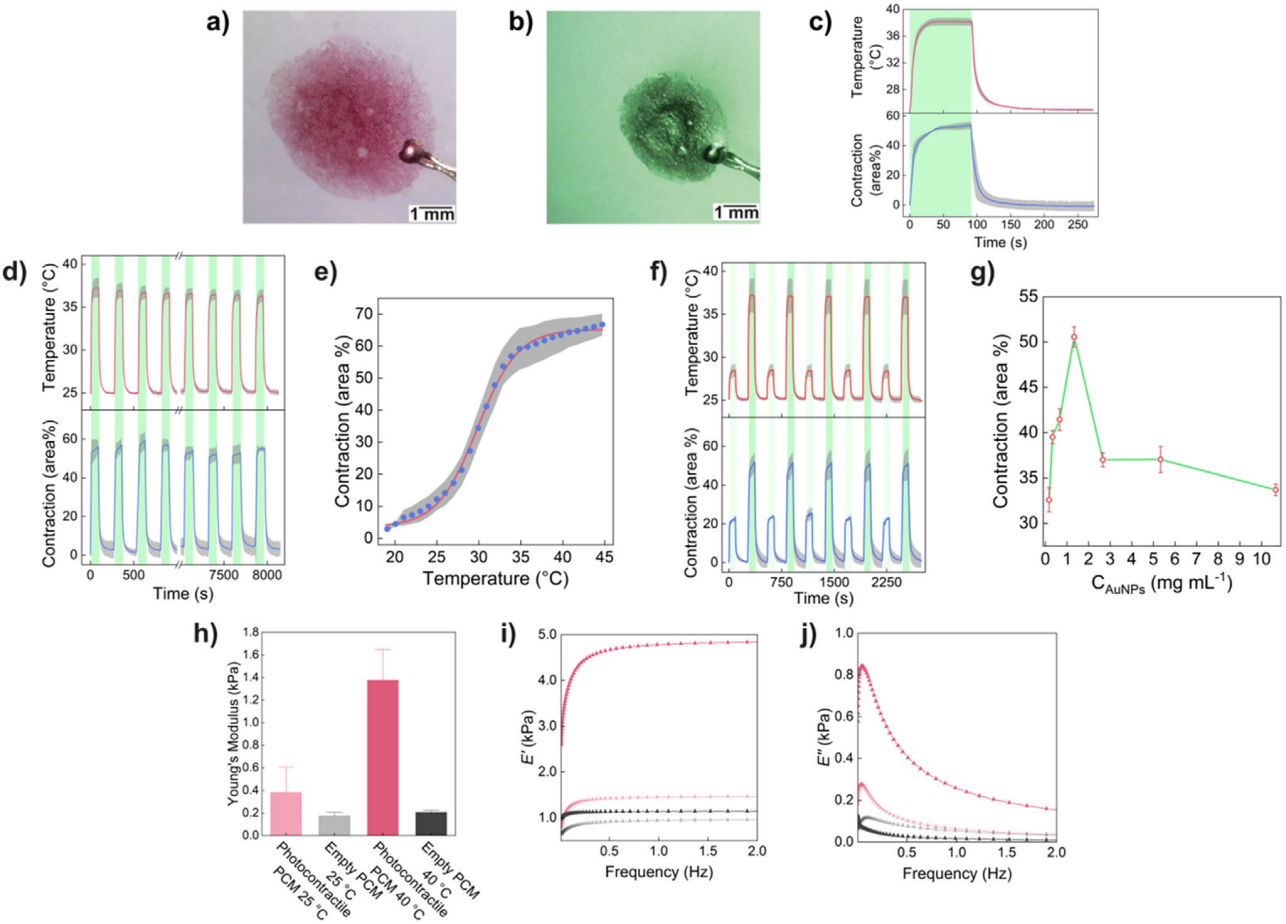
Light‐induced reversible contractions in prototissues. a) Photograph of a photo‐contractile prototissue composed of protocells endowed with a PNIPAM proto‐cortex and enclosing PEG‐AuNPs. The PCM was kept in the absence of light irradiation, at 25 °C, floating on water, and held on a thermocouple (bottom right metallic object). b) Photograph of the same PCM in (a) after 90 s of green light irradiation (*λ_max_* ≈520 nm, *Irr* = 1.35 W cm^−2^). c) Plot showing the material time‐dependent light‐induced contraction and concomitant localized temperature variation during a single light‐dark cycle (green area = light on, *Irr* = 1.35 W cm^−2^, Video S1, Supporting Information). d) Plot showing the material time‐dependent light‐induced contraction and concomitant localized temperature variation during 30 light/dark cycles (green areas: light on, *Irr* = 1.35 W cm^−2^). e) Plot showing the temperature‐dependent area variation of photo‐contractile prototissues (no light irradiation). f) Time‐dependent reversible contraction and concomitant temperature change in photonastic prototissues upon multiple cycles of irradiation with different light intensities (light green areas: *Irr* = 0.3 W cm^−2^, darker green areas: *Irr* = 1.35 W cm^−2^). Grey band in (c), (d), (e), and (f) = standard error calculated upon repeating the measurements on at least three independently prepared prototissue samples. g) Plot showing the photoinduced contraction of PCMs as a function of the amount of PEG‐AuNPs encapsulated in the protocell units that compose them (irradiation time: 120 s, *λ_max_* ≈520 nm, *Irr* = 1.35 W cm^−2^). Error bars = standard error calculated upon repeating the measurements on at least three independently prepared samples. h) Comparison of Young's modulus values between prototissues composed of protocells endowed with PNIPAM proto‐cortex (red) and prototissues composed of empty proteinosomes (grey) at 25 °C (light colors) and 40 °C (dark colors). i) Comparison of storage modulus (*E’*) values between prototissues composed of protocells endowed with proto‐cortex (red) and prototissues composed of empty protocells (grey) at 25 °C (light colors) and 40 °C (dark colors) (data with relative errors: Figure S42, Supporting Information). j) Comparison of loss modulus values (*E’’*) between prototissues composed of protocells endowed with proto‐cortex (red) and prototissues composed of empty protocells (grey) at 25 °C (light colored) and 40 °C (dark colored) (data with relative errors: Figure S42, Supporting Information).

Subsequently, we studied the photo‐contractile behavior of the PCMs assembled from a 1:1 binary population of bio‐orthogonal proteinosomes both encapsulating PNIPAM proto‐cortex and PEG‐AuNPs. For this, we irradiated (*λ* = 520 ± 20 nm, irradiance – *Irr* = 1.35 W cm^−2^) a PCM floating on Milli‐Q water thermostated at 25 °C (Section S1.5 and Figure S3, Supporting Information). Light irradiation of PCMs led to a fast contraction which reached a plateau of up to 50% of their initial area upon a concomitant localized increase in temperature (*ΔT*) of ≈13 °C after 90 s (**Figure** **2**a–c; Video S1, Supporting Information, details of how the area contraction of a prototissue was measured are given in Section S1.5, Supporting Information). This plateau corresponded to an energy dissipative stationary state, stable until the light was turned off. Moreover, PCMs showed excellent stability and durability. No drift in their contractile behavior (both contraction and *ΔT*) was observed over multiple light‐dark cycles, including 30 light‐dark cycles at 0–57% area contraction (Figure 2d) and 200 cycles at 47–57% area contraction (Figure S34a, Supporting Information. UV–vis spectroscopy analysis of the aqueous solution before and after fatigue testing revealed no AuNPs leakage and minimal to no loss of polymeric material (Figure S34b, Supporting Information). The photo‐contractile behavior was ascribed to the synergistic effect of the PEG‐AuNP photothermal properties and the thermally induced phase transition behavior of PNIPAM. When exposed to 520 nm light, the PEG‐AuNPs proto‐organelles inside the protocell lumen produced localized heat via the photothermal effect. This heat caused the thermosensitive PNIPAM proto‐cortex and membrane to shrink, resulting in rapid and reversible contractions of the entire prototissue. In fact, this was consistent with the thermally induced contractions of the tissue‐like material, as PCMs could reversibly contract of up to 70% upon going from 18 to 45 °C (Figure 2e). The extent of the contraction and the temperature at which the transition occurs (volume phase transition temperature – VPTT = 30 °C) did not seem to depend on the concentration of copolymers (1) and (2) encapsulated inside the proteinosomes (Figure S35, Supporting Information). Importantly, the presence of PEG‐AuNPs did not influence the thermoresponsive behavior of the prototissues, despite the presence of PEG, which is well known to increase the volume phase transition temperature of PNIPAM‐based hydrogels and broaden its profile (Figure S36, Supporting Information).^[^ ^32^, ^33^ ^]^ This was ascribed to the fact that the PEG‐AuNPs were not covalently bound to the PNIPAM proto‐cortex and were entrapped within the aqueous lumen of the protocell units.

To provide further evidence that the photothermal increase in temperature was the phenomenon that induced the volume phase transition of the PNIPAM polymer network contained in the material, we investigated the PCM contraction upon variation of the irradiance of the light source (Figure 2f; Figure S37, Supporting Information). In a set of dynamic experiments, upon varying the irradiance, we verified that the contraction and the *ΔT* could be modulated and cycled with excellent reproducibility (Figure 2f). Moreover, in a set of static experiments performed by irradiating the prototissue at variable irradiance for a fixed time (120 s), we verified that the photoinduced contraction reached a plateau beyond *Irr* = 0.9 W cm^−2^, while *ΔT* increased linearly over the whole range of irradiance explored (Figure S37, Supporting Information). This is due to the fact that being the system thermostated at 25 °C, with *Irr* = 0.9 W cm^−2^, *ΔT* already exceeds 8 °C. At such photon flux, the prototissue was close to the temperature of its maximum contraction, i.e., ≈33 °C. Further investigation of the photothermal contraction was performed by varying the starting temperature (*T_light OFF_*, Figure S38, Supporting Information) at a fixed irradiation of 1.35 W cm^−2^. As expected, we observed a drop of the PCM contraction from 25 °C and no substantial decrease of *ΔT*. This was attributed to the fact that the PCM already reached a contracted state prior to light irradiation.

We then investigated the effect of PEG‐AuNPs concentration on the photoinduced PCM contraction (Figure 2g). The results showed a sharp increase in the maximum contraction with increasing PEG‐AuNPs concentration from 0.2 to 1.33 mg mL^−1^, followed by a sharp decrease and a plateau at ≈35% contraction when the concentration of PEG‐AuNPs exceeded 2 mg mL^−1^. Initially the PCM contraction increased rapidly with PEG‐AuNPs concentration due to an increase in photon absorption and localized photothermal heating, demonstrating the critical role of PEG‐AuNPs as photothermal transducers. At concentrations above 1.33 mg mL^−1^ a filtering effect was instead observed due to the first layers of the PCM closer to the light source absorbing most of the incident light, causing inhomogeneous heating of the material. UV–vis spectroscopy of the same prototissues corroborated these findings, revealing a progressive decline in transmittance with increasing PEG‐AuNPs concentration. At concentrations above 2 mg mL^−1^, transmittance dropped below 10% and almost reached 0% at 10.63 mg mL^−1^ (Figure S39a,b, Supporting Information). Notably, the plasmon resonance band remained stable at 527 nm across all concentrations, indicating minimal to no PEG‐AuNPs aggregation (Figure S39c, Supporting Information).

We complemented these initial studies by examining the influence of prototissue thickness on the photoinduced contraction (Figure S40, Supporting Information). For this, we varied the prototissue thickness from ≈50 to 170 µm by injecting increasing volumes of 1:1 binary emulsion volume into the floating PTFE mold.^[^ ^8^ ^]^ Interestingly, we observed a maximum extent of photoinduced contraction with the prototissues ≈100 µm thick. This bell‐shaped trend seems to be consistent with a filtering effect: at low thicknesses, the prototissue has less light absorption, resulting in a lower temperature increase, whereas at higher thicknesses, the low transparency of the PCM leads to inhomogeneous heating, as the more superficial layers of the PCM closer to the light source absorb most of the incident light.

The temperature‐dependent mechanical properties of the prototissue were characterized by micro‐indentation (Sections S1.7 and S5, Supporting Information). This allowed for the determination of the soft material Young's modulus (*E*) (Figure 2h), the instantaneous and equilibrium moduli (measure of the instantaneous material response, *E_0_*, and of the material response after complete viscous relaxation, *E_∞_*, respectively, Figure S41, Supporting Information), and the storage and loss moduli (*E’* and *E’’*, respectively, determined at 2 Hz; Figure 2i,j; Figure S42, Supporting Information). All moduli were measured at 25 °C and at 40 °C, and are summarized in **Table** **1** (vide infra). As expected, the photo‐contractile prototissues, whose proteinosomes are endowed with a proto‐cortex, were more robust than the PCMs formed from “empty” proteinosomes.^[^ ^8^ ^]^ Passing from 25 to 40 °C the different moduli of photo‐contractile prototissues increased drastically due to the thermoresponsive properties of PNIPAM. In contrast, the moduli of prototissues assembled from empty proteinosomes showed a minimal increase with the temperature, even though the proteinosome membranes were composed of thermoresponsive BSA/PNIPAM‐*co*‐MAA.

**Table 1:** Table summarizing the temperature‐dependent mechanical properties of the different types of prototissues synthesized and used in this work.

Temperature [°C]	Moduli [kPa]	Prototissues assembled from proteinosomes endowed with a PNIPAM proto‐cortex	Prototissues assembled from empty proteinosomes	Prototissues assembled from proteinosomes endowed with a PDMAM proto‐cytoskeleton
25	*E*	0.4 ± 0.2	0.17 ± 0.04	1.3 ± 0.5
	*E_0_*	1.5 ± 0.5	1.0 ± 0.1	2.7 ± 0.3
	*E_∞_*	0.9 ± 0.3	0.70 ± 0.05	2.4 ± 0.3
	*E’* (at 2 Hz)	1.5 ± 0.5	1.0 ± 0.1	2.6 ± 0.4
	*E’*’ (at 2 Hz)	0.03 ± 0.02	0.03 ± 0.02	0.018 ± 0.005
40	*E*	1.4 ± 0.2	0.21 ± 0.02	0.7 ± 0.1
	*E_0_*	4.5 ± 0.3	1.2 ± 0.1	2.1 ± 0.3
	*E_∞_*	2.8 ± 1.0	1.0 ± 0.1	1.7 ± 0.2
	*E’* (at 2 Hz)	4.8 ± 1.1	1.1 ± 0.1	2.1 ± 0.4
	*E’*’ (at 2 Hz)	0.15 ± 0.07	0.008 ± 0.001	0.016 ± 0.004

**Figure 3:**
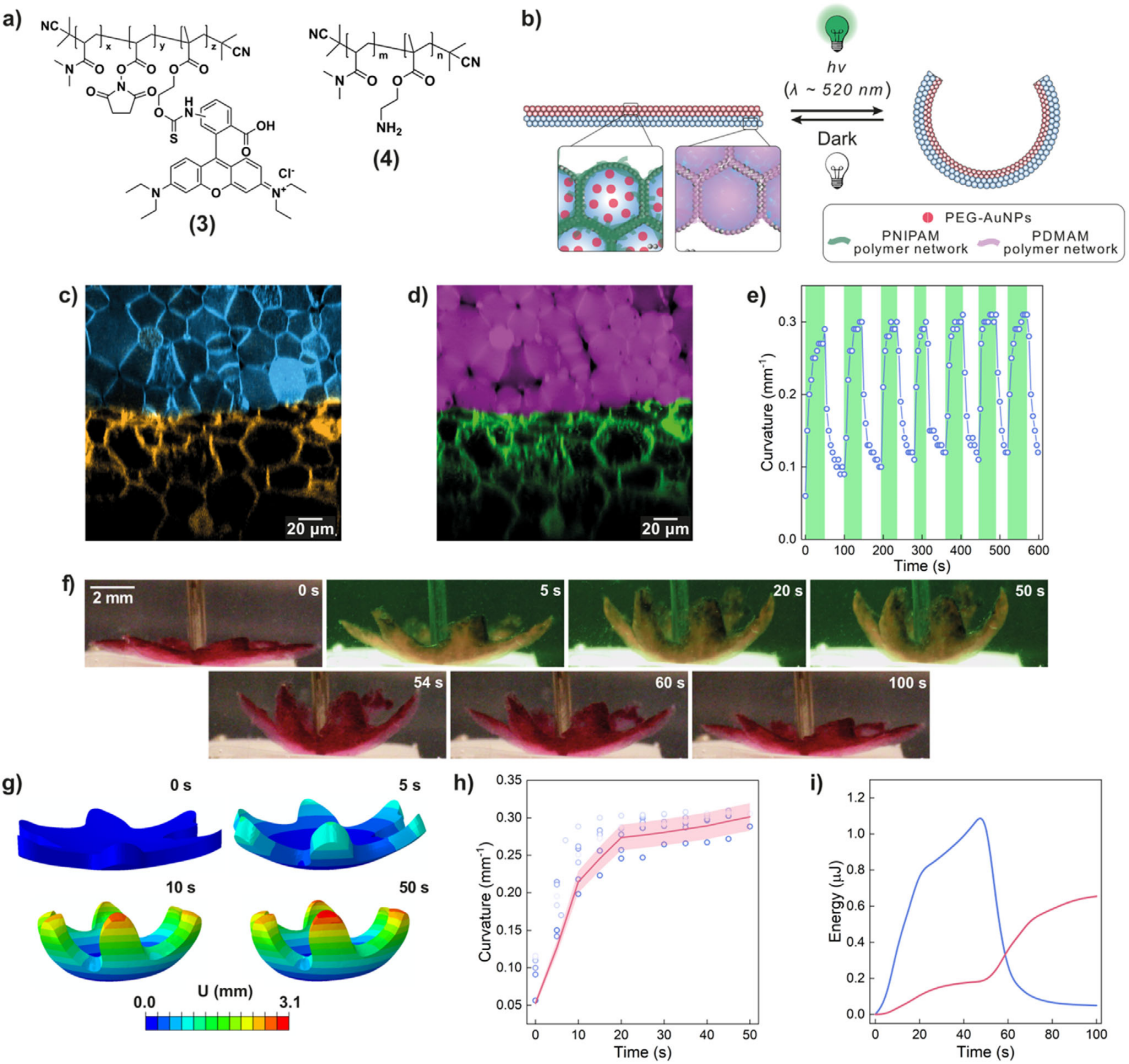
Structure and movement characterization of a photonastic prototissue. a) Molecular structures of the PDMAM‐based copolymers (3) and (4) used to build the non‐thermoresponsive protocell proto‐cytoskeleton. b) Scheme describing the working principle of the photonastic prototissue bilayer. The top layer was composed of photo‐contractile protocells endowed with a PNIPAM proto‐cortex and encapsulating PEG‐AuNPs light‐transducing proto‐organelles. The bottom layer was made of non‐contractile protocells endowed with a PDMAM proto‐cytoskeleton. Light irradiation induces photothermal heating, contraction and stiffening of the top layer, while causing a decrease in rigidity of the bottom layer, overall leading to a reversible bending of the prototissue. c,d) XZ orthogonal plane of a confocal fluorescence Z‐stack obtained by imaging the bilayer prototissue described in (b) at the interface between the two layers (composition: Section S4.2, Supporting Information). c) Fluorescence channels corresponding to the bio‐orthogonally reactive protein‐polymer nanoconjugates: AMCA‐labeled azide‐ or BCN‐functionalized BSA/PNIPAM‐*co*‐MAA nanoconjugate (blue fluorescence), and BDP650‐labeled azide‐ or BCN‐functionalized BSA/PNIPAM‐*co*‐MAA nanoconjugate (orange fluorescence). d) Fluorescence channels corresponding to the FITC‐labeled PNIPAM proto‐cortex (green fluorescence) and RITC‐labeled PDMAM proto‐cytoskeleton (purple fluorescence). e) Plot of the photonastic prototissue bilayer curvature over seven light‐dark cycles (*λ_max_* = 520 nm, *Irr* = 1.35 W cm^−2^, Video S2, Supporting Information). f) Image sequence showing the first light/dark cycle in (e) and the reversible bending of the photonastic prototissue. g) Magnitude displacement fields U (mm) on the photonastic prototissue in (f) at time 0, 5, 10, and 50 s obtained from the FEM numerical simulation of the light‐dark cycle. h) Comparison of FEM numerical results (red line) and experimental data (blue points, colors from darker to lighter correspond to 1st to 7th light‐dark cycles) of the time‐dependent curvature changes of the photonastic prototissue. The curvature from the numerical model was calculated along six radial directions and reported as mean ± standard deviation (light red band). i) Time‐dependent changes in the stored strain energy (blue line) and dissipated viscous energy (red line) evaluated by the FEM model for a single light/dark cycle (0–50 s irradiation, 51–100 s light off).

Excited by the possibility of using light to induce fast and reversible contractions in our tissue‐like materials, we next showcased that by capitalizing on the multi‐microcompartmentalized nature of the PCMs and on the possibility of generating complex 3D architectures using the floating mold technique, we could fabricate tissue‐like materials endowed with a higher‐order photonastic behavior from the bottom‐up. To achieve this objective, we started by fabricating novel, non‐thermoresponsive prototissues from non‐thermoresponsive protocell units. To assemble the latter, poly(*N,N*‐dimethylacrylamide) (PDMAM)‐based copolymers (**3**) and (**4**) (**Figure** **3**a) were mixed with the azide‐ or BCN‐functionalized BSA/PNIPAM‐*co*‐MAA nanoconjugate in Na_2_CO_3_ buffer (pH 8.5, 100 mm), and emulsified (Section S3.3 and Figure S43, Supporting Information). Similarly to what described previously, within the water‐in‐oil microdroplet stabilized by a membrane of bio‐orthogonally reactive BSA/PNIPAM‐*co*‐MAA nanoconjugate, copolymer (**3**) reacted with copolymer (**4**) to generate an internal polymer network and reacted with the residual interfacial amines of the protein‐polymer nanoconjugate to crosslink and stabilize the proteinosome membrane for the subsequent PCM generation and transfer to water.

Compared to the photo‐contractile proteinosomes, these PDMAM‐based proteinosomes in oil presented a prevalently spherical morphology, with a mean equivalent diameter of 13 ± 5 µm (mean volume ≈1.2 pL) and a roundness of 0.90 ± 0.05 (Figure S43, Supporting Information). The bio‐orthogonally reactive BSA/PNIPAM‐*co*‐MAA nanoconjugates formed a regular and well‐defined self‐assembled membrane at the oil/water interface, whereas the PDMAM polymer network showed a homogeneous distribution within the protocell lumen. Seen that the polymer network was distributed homogeneously inside the proteinosome protocytoplasm, in analogy with similar systems,^[^ ^6^, ^30^, ^31^, ^34^ ^]^ we termed the PDMAM polymer network “proto‐cytoskeleton”. This was different from the PNIPAM‐based “proto‐cortex”, which was instead a polymer network mostly located at the inner edge of the protocell membrane. This difference in the distribution of the two types of polymer networks was in line with the more hydrophilic character of PDMAM compared to PNIPAM. During the emulsification process, PDMAM remained dissolved in the aqueous phase, whereas the PNIPAM tended to segregate at the water/oil interface due to its amphiphilic character.

Non‐thermoresponsive PCMs could then be fabricated from a 1:1 population of azide‐ and BCN‐functionalized PDMAM‐filled proteinosomes in oil using the floating mold technique (Figures S44 and S45, Supporting Information). The temperature‐dependent mechanical properties of these prototissues were then characterized using micro‐indentation. These PCMs composed of protocells endowed with a PDMAM proto‐cytoskeleton at 25 °C were found to be three times more elastic than the photo‐contractile PCMs. At 40 °C they showed instead a slight decrease of elastic properties and an almost unchanged viscous behavior due to the non‐thermoresponsive nature of PDMAM (Table 1, Section S5, Figures S46–S48, Supporting Information).

To obtain a prototissue capable of a higher‐order photonastic behavior, we employed the floating mold technique to generate a stratified prototissue in the shape of a six‐armed starfish, consisting of a bottom layer of non‐thermoresponsive PDMAM‐filled protocells and a top layer of photo‐contractile PNIPAM‐based protocells Figure 3b–f (Section S4.2, Figure S49, Supporting Information). In particular, Figure 3c,d shows that upon PCM assembly, the individual protocell building blocks retained their original structure and composition, without leakage of the polymeric constituents of the PNIPAM‐based proto‐cortex or of the PDMAM‐based proto‐cytoskeleton throughout the material. The thickness of the two layers of the stratified six‐armed starfish‐shaped prototissue corresponded to 176 ± 9 µm and 480 ± 30 µm for the photo‐contractile and non‐contractile layers, respectively (Figure S49, Supporting Information).

Irradiation of the stratified PCM from the top (*λ_max_* = 520 nm, *Irr* = 1.35 W cm^−2^) caused a fast and reversible closure of the six arms toward the light source due to selective contraction of the top photo‐contractile layer, as shown in Figure 3e,f and Video S2 (Supporting Information). The material could reversibly undergo seven bending‐relaxing cycles, going from an initial curvature of 0.06 to 0.29 mm^−1^ after 50 s of irradiation. After the first irradiation cycle, the PCM showed stable initial and final curvature values over repeated light‐induced bending‐relaxing cycles, demonstrating excellent stability.

Thanks to our ability to characterize the mechanical properties of the two types of prototissue that composed the photonastic stratified prototissue, we studied its mechanical response to light irradiation through numerical analyses based on the Finite Element Method (FEM—see Section S6, Supporting Information). The geometry of the model was deduced by a segmentation procedure starting from digital images of the sample and assuming a constant thickness for the PNIPAM (176 mm) and the PDMAM (480 mm) layers. The PNIPAM and PDMAM layers were modeled as isotropic linear viscoelastic materials, with equilibrium elastic moduli depending on the temperature, according to experimental data (Section S5, Supporting Information). Nonlinear quasi‐static analyses were carried out to simulate the deformation path of the structure under a light irradiation half‐cycle of 50 s. Temperature‐time function and PNIPAM contraction‐temperature function were deduced from experimental data (Figure 2c). The deformation of the structure in the half‐cycle of irradiation is shown in Figure 3g. The contraction of the PNIPAM layer induced a bending of the structure that described well the data of curvature as a function of time obtained in the experiment (Figure 3h). The good agreement between experimental and simulated curvature data confirmed that the reversible bending of the photonastic prototissue is governed by two synergistic mechanisms: the thermally induced variation in mechanical properties between the PNIPAM and PDMAM layers, and the structural continuity achieved through I‐SPAAC bonding that couples the light‐responsive and non‐responsive layers. This covalent integration ensures efficient strain transfer, allowing localized photothermal contraction in the active layer to generate a propagating strain field that bends the entire structure while maintaining self‐equilibrium. Furthermore, the numerical simulation also allowed us to estimate the energy quantities over time in terms of strain energy (stored energy that can be recovered in the light‐off phase) and dissipated energy due to viscous phenomena present during both the irradiation and the light‐off phases (Figure 3i).

From a general perspective, our rational design and the bottom‐up assembly of protocell building blocks into multi‐micro‐compartmentalized materials demonstrate a powerful strategy for achieving the next generation of prototissues. In particular, the photonastic behavior exhibited by our prototissues emerged from the synergistic interplay of four key components: i) the interfacial bio‐orthogonal reactivity of the protocell membrane that enables direct protocell‐protocell adhesions, ii) the selective membrane permeability that enables polymer and nanoparticle retention while allowing water molecule mobility, iii) the photothermal transduction by PEG‐AuNPs proto‐organelles, and iv) the thermoresponsive properties of the PNIPAM‐based proto‐cortex. This highly modular approach, characterized by chemically programmable proteinosomes and multi‐micro‐compartmentalization, not only allows for the construction of prototissues capable of light‐induced complex motions, such as our starfish‐like prototissue, but also promises other interesting possibilities, vide infra.

### 2.2 Photo‐Mechano‐Chemical Transduction Within a Prototissue

Having established the possibility of fabricating free‐standing prototissues capable of a higher‐order photonastic behavior, we hypothesized that the light‐induced contraction of the prototissue could be used to engineer a form of photo‐mechano‐chemical transduction within prototissues, where the light‐induced contraction of the material could down‐regulate or even switch off an internalized enzyme metabolism hosted within the constituent protocells.

**Figure 4:**
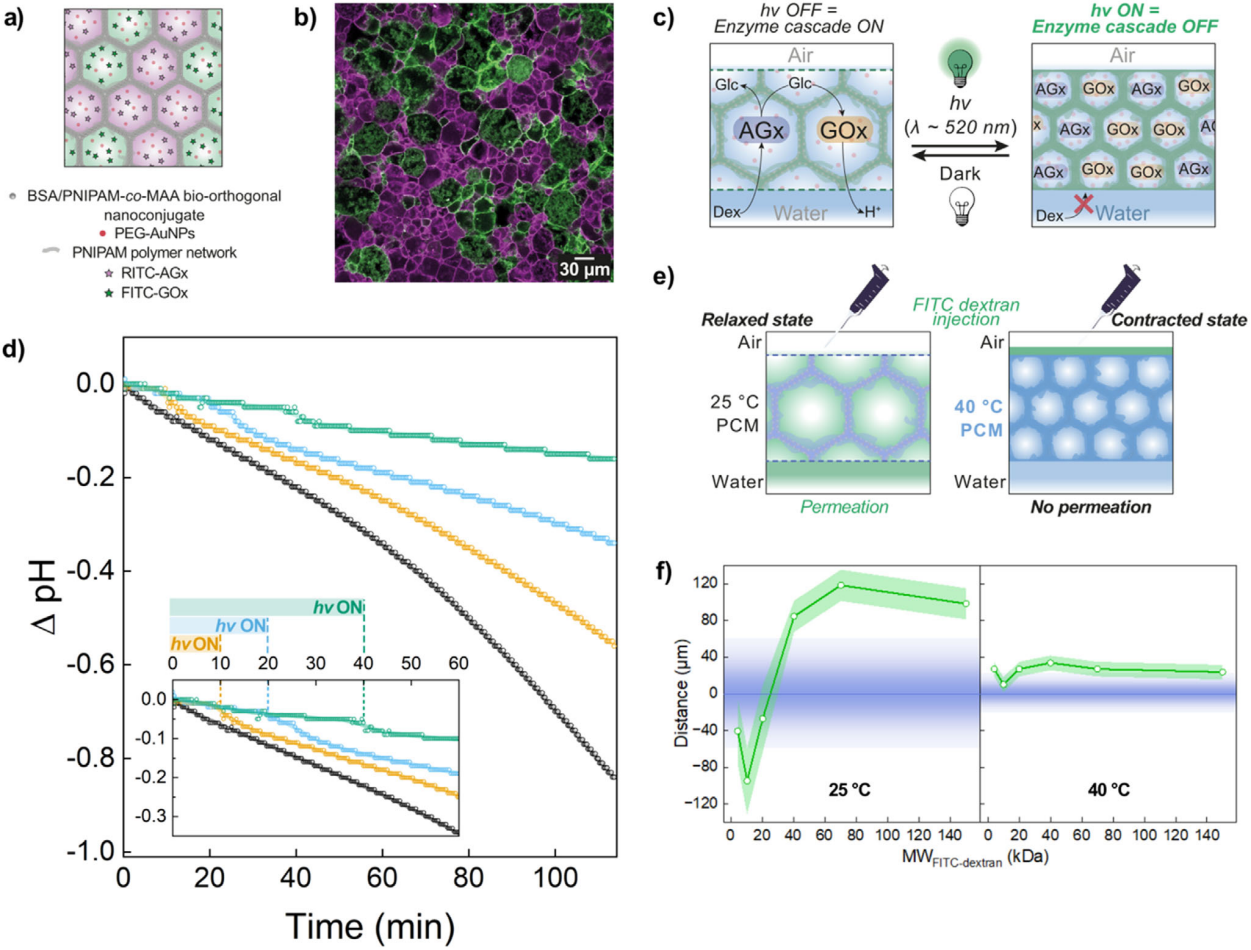
Prototissues capable of photo‐mechano‐chemical transduction. a) Scheme showing the structure of a prototissue which comprises interconnected protocells composed of a membrane of non‐tagged azide‐ or BCN‐functionalized BSA/PNIPAM‐*co*‐MAA nanoconjugate, a non‐tagged PNIPAM proto‐cortex, and PEG‐AuNPs photothermal transducing proto‐organelles. The azide‐functionalized protocells encapsulate RITC‐tagged AGx, whereas the BCN‐functionalized protocells encapsulate FITC‐tagged GOx. b) Confocal fluorescence microscopy image of the prototissue in (a), highlighting the compartmentalization of the two enzymes (RITC‐labeled AGx, purple fluorescence; FITC‐labeled GOx, green fluorescence). c) Scheme highlighting the working principles of the photo‐mechano‐chemical transduction mechanism: the light‐induced contraction of the prototissue causes the reversible impermeabilization of the membrane of the protocells that compose the prototissue, with a consequent switch‐off of the AGx/GOx enzyme cascade reaction hosted within the prototissue. d) Time‐dependent pH changes of a PBS buffer solution (1 mm, initial pH 6.2) containing 12.6 µL of 80 mg mL^−1^ solution of dextrin in PBS (2 mm, pH 6.2) and a prototissue assembled from a 1:1 binary population of photo‐contractile proteinosomes encapsulating AGx or GOx. The black plot represents the experiment performed in the absence of light irradiation. The yellow, light blue, and green plots represent kinetic experiments performed by pre‐irradiating the prototissue for 3 min in order to contract them completely before the addition of Dex, and then by irradiating the PCM for 10, 20, and 40 min, respectively (*λ_max_* = 520 nm, *Irr* = 1.35 W cm^−2^). The plots show that as soon as the light was switched off, the pH started to decrease promptly, indicating the start of the enzyme cascade reaction. e) Scheme explaining the characterization of the temperature‐dependent permeability of the prototissues. In these experiments, a prototissue was used to filter aqueous solutions of FITC‐labeled dextran of different molecular weights. If the molecular weight of the FITC‐labeled dextran was smaller than the MWCO of the PCM the polysaccharide diffused into the bulk water underneath the prototissue, otherwise it remained segregated above it. f) Reciprocal position of the maxima of fluorescence Z‐intensity profiles acquired for AMCA‐labeled prototissues and FITC‐labeled dextran as a function of the molecular weight of the dextran. Experiment performed at 25 °C (left) and 40 °C (right). The blue line represents the position of the prototissue (maximum of Z‐profile relative to blue fluorescence channel—Figure S53, Supporting Information), and the light blue bands represent the full width at half maximum (FWHM) of the blue distribution curve, providing an estimation of the thickness of the prototissue. The white points represent the position of the FITC‐labeled dextran with respect to the PCM (maximum of Z‐profile relative to green fluorescence signal—Figure S53, Supporting Information), the light green band represents the FWHM of the green distribution curve. The plots show that when the prototissue was in the relaxed state, its MWCO was *≈*25 kDa, whereas when it was in the contracted state (40 °C), the membrane became completely impermeable to hydrophilic solutes.

To do so, we prepared a 5 mm diameter circular prototissue from a binary population of photo‐contractile protocells containing either AGx or GOx enzymes (Section S3.2c, Supporting Information). Structural analysis by confocal fluorescence microscopy confirmed that the two differently tagged enzymes could be retained in separate proteinosomes populations within the same PCM (**Figure** **4**a,b). The PCM was suspended in 1 mm phosphate buffer solution (PBS) at pH 6.2 in a thermostated chamber at 25 °C (Section S1.8, Supporting Information). In the absence of light, injection of a solution of dextrin (12.6 µL, 80 mg mL^−1^) within the aqueous medium initiated the spatially coupled enzyme cascade reaction. Dextrin diffused within the AGx‐containing protocells, where it was catalytically hydrolyzed to glucose, which in turn acted as the signaling molecule diffusing into the protocells containing GOx. GOx subsequently converted glucose into D‐glucono‐1,5‐lactone (GDL), which spontaneously hydrolyzed to form gluconic acid. This caused a local decrease of the pH of the bulk aqueous solution (*ΔpH* ≈−1 after 120 min from substrate injection, Figure 4c,d—black plot). This mechanism was confirmed by a control experiment carried out under the same conditions but in the absence of AGx, which did not show any pH drop upon addition of dextrin (Figure S50, Supporting Information). This experiment therefore indicated that i) the presence of AGx was necessary to hydrolyze dextrin to glucose and initiate the spatially coupled AGx/GOx cascade reaction, and ii) that glucose was not present in sufficient concentration inside our substrate solution to induce a pH drop by a direct GOx‐catalyzed gluconic acid production.

In contrast, injection of dextrin under the same conditions with the only exception that the AGx/GOx‐containing PCM was in the contracted state (i.e., exposed to light *λ_max_* = 520 nm; *Irr* = 1.35 W cm^−2^) caused the complete inhibition of the enzyme cascade reaction. Importantly, as the photoinduced PCM contractility was fast and reversible, the spatially coupled AGx/GOx reaction could be switched on at any time by turning the light off and allowing the material to relax back to its original state (Figure 4d).

During irradiation and reaction inhibition, the trend follows a slight pH drift due to the instability of the buffer, but when the light was turned off there was always a clear pH drop due to a prompt production of gluconic acid. A decrease in the speed of pH variation with the increasing irradiation time was also noticed (Figure 4d). This was attributed to a decrease of the encapsulated enzyme activity following continued localized heating due to light irradiation. The key role of the photonastic tissue‐like material on the photo‐mechano‐chemical transduction mechanism was then confirmed by performing the same enzyme cascade kinetic experiment under the same experimental conditions but in the absence of the prototissue architecture, i.e., with the free enzymes in the bulk aqueous solution (Figure S51, Supporting Information). As expected, this control experiment showed no influence of the light irradiation on the enzyme‐mediated production of gluconic acid, and, consequently, on the decrease of pH.

The inhibition of the enzyme cascade reaction in the presence of light was ascribed to a marked change in the permeability of the protocells as the prototissue transitioned from the relaxed to the contracted state. Luminous energy converted to localized heat by the PEG‐AuNPs proto‐organelles triggered a material contraction. This behavior stemmed from the physical and chemical properties of PNIPAM which comprises both the protocell membrane and the proto‐cortex. The contraction reduced the polymer network mesh size of the proto‐cortex, shifting the material from a hydrophilic, hydrated state that allowed small hydrophilic substrate molecules to pass through, to a hydrophobic state that blocked their passage (Figure 4c). This was verified by studying the temperature‐dependent molecular weight cut‐off (MWCO) of the photo‐contractile PCMs. For this, the prototissues were utilized to filter, first at 25 °C and subsequently at 40 °C, a series of aqueous solutions of FITC‐labeled dextrans of molecular weights ranging from 4 to 150 kDa (Figure 4e,f; Section S1.9, Table S3, Figures S52 and S53, Supporting Information). Confocal fluorescence microscopy was used to determine whether the fluorescent polymers could flow through the PCM and diffuse into the underlying bulk aqueous phase or not. Figure 4f, the left plot shows that at 25 °C (i.e., PCM in a relaxed state) the MWCO associated to the PCMs with PNIPAM proto‐cortex was equivalent to ≈25 kDa, whereas Figure 4f, the right plot shows that at 40 °C (i.e., PCM in the contracted state) the PCMs were completely impermeable to the dextrans in the whole molecular weight range tested. These results are in agreement with the GPC characterization of the molecular weight of the dextrin used as a substrate for the AGx/GOx enzyme cascade reaction (Figure S54 and Table S4, Supporting Information). The dextrin substrate that was used (Figure 4c) had an *M_n_* = 11.7 ± 0.4 × 10^3^ g mol^−1^ and a dispersity *Ð* = 2.23 ± 0.08 and was therefore capable of permeating through the protocell membranes and cortex at 25 °C, but not with the PCM in the contracted state. This remarkable reduction of MWCO with respect to our previously reported prototissue spheroids (≈80 kDa at 25 °C, and ≈30 kDa at 47 °C)^[^ ^7^ ^]^ highlights the key role of the PNIPAM‐based proto‐cortex in the photo‐mechano‐chemical transduction mechanism.

## 3 Conclusion

As a step toward the bottom‐up chemical construction of fully autonomous tissue‐like materials, we combined in an original manner bio‐orthogonal chemistry,^[^ ^7^ ^]^ the floating mold technique,^[^ ^8^ ^]^ and our ability to chemically program the behavior of individual protocell units, to assemble prototissues capable of higher‐order photonastic and energy transduction behaviors. For this, we synergistically combined the bio‐orthogonal adhesion of the protocell membrane, the photothermal properties of 14 nm gold nanoparticle (PEG‐AuNP) proto‐organelles, and the thermoresponsive characteristics of the synthetic PNIPAM‐based protocell membrane and cortex. This approach enabled the assembly of prototissues capable of light‐induced, coordinated, and rapid reversible contractions. When irradiated with 520 nm light, the PEG‐AuNPs proto‐organelles within the protocell lumen generated localized heat through their photothermal effect, causing the thermoresponsive PNIPAM‐based proto‐cortex and membrane of all protocells composing the material to contract. When the light was turned off, rapid heat dissipation through the surrounding water medium enabled the prototissue to return to its original size and shape. The photonastic contractile behavior of our prototissues was governed by the following four key parameters: 1) the environmental temperature, which must be maintained slightly below the material VPTT to enable light‐triggered contraction when the photothermal effect raises the local temperature above this threshold; 2) the incident light intensity, which serves as a tunable parameter to modulate the contraction magnitude; 3) the concentration of PEG‐AuNPs photothermal transducing proto‐organelles, which directly influences the efficiency of light‐to‐heat conversion; finally, 4) the prototissue thickness, which affects both light penetration depth and heat dissipation, thereby impacting the spatial uniformity and kinetics of the contractile response. We then fabricated a stratified prototissue with the shape of a six‐armed starfish that comprised a top layer of photo‐contractile protocells, and a bottom layer of non‐contractile protocells. When irradiated with 520 nm light, the six arms of the prototissue started to close, and when the light was turned off the arms returned to their resting position. The reversible bending of the photonastic prototissue was governed by two synergistic mechanisms: the thermally induced variation in mechanical properties between the PNIPAM and PDMAM layers, and the structural continuity between the two prototissue layers achieved through I‐SPAAC adhesions. In particular, the latter element ensured efficient strain transfer, allowing localized photothermal contraction in the active layer to generate a propagating strain field that bent the entire structure while maintaining self‐equilibrium. Finally, we demonstrated that the photo‐mechanical transduction mechanism not only drives the conversion of light energy into a macroscopic material contraction but also reduces the polymer network mesh size in both the protocell membranes and proto‐cortexes. This reduction triggers a reversible shift in the material's physicochemical properties from a hydrated, hydrophilic state that is permeable to hydrophilic solutes, to a hydrophobic state that is impermeable to hydrophilic molecules. By exploiting this complex photoresponsive behavior, we engineered a prototissue capable of photo‐mechano‐chemical transduction. Controlled light irradiation maintained the material in an impermeable, metabolically inert state by excluding hydrophilic substrates. Subsequent removal of illumination restored permeability, dynamically activating a spatially coordinated AGx/GOx enzyme cascade within the protocell network. Both the photonastic and the photo‐mechano‐chemical transduction higher‐order behaviors emerged from the collective interactions and coordinated responses that were facilitated by the spatial integration of the protocell building blocks within the prototissue.

From a general perspective, our work shows that the rational engineering of protocell units and their programmed assembly into prototissues represents a valid approach to achieving advanced and effective energy transduction mechanisms within synthetic materials, exploitable for the engineering of higher‐order behaviors. A key factor in our approach was the ability to co‐assemble and spatially integrate different protocell units that synergistically combined their specialized components with the collective properties of the ensemble. As such, our general approach and results fill an important gap in bottom‐up synthetic biology and active matter research. Beyond advancing the fundamental understanding of biological behaviors based on physicochemical principles, our platform paves the way for the future development of synthetic multi‐compartmentalized materials with embedded feedback systems. The photonastic prototissues developed here, exhibiting photo‐mechano‐chemical transduction, hold immediate promise for cell‐protocell integration and tissue engineering. Specifically, they represent a novel class of chemically programmable substrates capable of delivering both chemical and mechanical cues to adherent living cells. For instance, the bottom‐up design of these materials allows precise tuning of their static mechanical properties. The PNIPAM‐ and PDMAM‐based prototissues studied here mimic the elastic moduli of, for example, brain and lung tissues (0.3–5 kPa, respectively).^[^ ^35^, ^36^ ^]^ However, by modulating crosslinking density^[^ ^24^ ^]^ or selecting alternative polymer systems, the elastic modulus can be systematically tuned to match that of stiffer tissue types. Our prototissue versatility is not limited to static mechanical properties. Our green‐light‐responsive system could be used to deliver dynamic mechanical stimuli (10–20% area contraction at biocompatible irradiance of 150–300 mW cm^−2^), offering a non‐invasive means to guide cell differentiation and spatial organization. Future developments could also exploit near‐infrared photothermal transducing proto‐organelles (e.g., gold nanorods, graphene oxide) for applications in deep‐tissue regenerative medicine. Finally, the applications of these dynamic prototissues extend beyond tissue engineering and medicine, with the possibility of using them as modular micro‐bioreactors that can be opened/closed with light stimuli or as soft actuators for biohybrid robotics.

## 4 Experimental Section

Detailed descriptions of the materials and instruments used, experimental procedures, and methods are provided in the Supporting Information.

### Synthesis of Azide‐ or BCN‐Functionalized BSA/PNIPAM‐*co*‐MAA Nanoconjugates

Aminomethyl coumarin (AMCA) NHS ester or BODIPY 650/665 X (BDP 650) NHS ester‐labeled, and non‐labeled azide‐ and BCN‐functionalized BSA/PNIPAM‐*co*‐MAA nanoconjugates were synthesized and characterized according to the general procedure established previously.^[^ ^7^ ^]^

### Synthesis of Copolymers (1) and (2) and Hydrogel Preparation

Copolymers (**1**) and (**2**) were synthesized via reversible addition‐fragmentation chain transfer (RAFT) polymerization Copolymer (**1**) had an *M_n_* of 125.2 ± 0.8 × 10^3^ g mol^−1^ (*Ð* = 1.05 ± 0.01) and a lower critical solution temperature (LCST) of 30 °C; whereas copolymer (**2**) had an *M_n_* of 65.3 ± 0.6 × 10^3^ g mol^−1^ (*Ð* = 1.14 ± 0.01) and an LCST of 32 °C (Sections S2.2, S2.3 and Figures S7–S10, S17a,b, Supporting Information). Copolymer (**2**) was also fluorescently labeled with either fluorescein isothiocyanate (FITC) or rhodamine B isothiocyanate (RITC) for structural characterization of PCMs via fluorescence imaging (Sections S2.3.1 and S2.3.2, Figure S11, Supporting Information). Copolymers (**1**) and (**2**) successfully formed a bulk covalent hydrogel via amide bond formation between the NHS ester of copolymer (**1**) and the amine of copolymer (**2**) when their solutions were mixed in Na_2_CO_3_ buffer (pH 8.5, 100 mm) at a final concentration of 30 mg mL^−1^ for ≈2 h (Figure S18a–f, Supporting Information). The hydrogel showed a reversible volume phase transition temperature (VPTT) of 30 °C (Figure S19a, Supporting Information), which was in line with the phase transition behavior shown by its polymeric components (Figure S17a,b, Supporting Information).

### Synthesis of Copolymers (3) and (4) and Hydrogel Preparation

Copolymers (**3**) and (**4**) were synthesized via free radical polymerization. Copolymer (**3**) had an *M_n_* of 37 ± 9 × 10^3^ g mol^−1^, (*Ð* = 2.5 ± 0.2), copolymer (**4**) had an *M_n_* of 340 ± 120 × 10^3^ g mol^−1^ (*Ð* = 2.8 ± 0.9), both copolymers did not show an LSCT in temperature range studied (Sections S2.5 and S2.6, and Figures S13–S16, S17c,d, Supporting Information). Copolymers (**3**) and (**4**) readily formed a bulk non‐thermoresponsive covalent hydrogel via amide bond formation between the NHS ester of copolymer (**3**) and the amine of copolymer (**4**) when their solutions were mixed in Na_2_CO_3_ buffer (pH 8.5, 100 mm) at a final concentration of 30 mg mL^−1^ for ≈2 h (Figures S18g–l and S19b, Supporting Information).

### Synthesis of Poly(ethylene glycol)‐Stabilized Gold Nanoparticles (PEG‐AuNPs)

PEG‐AuNPs were prepared using a classical Turkevich synthetic procedure (Section S2.4 and Figure S12, Supporting Information), and stabilized with a thiolated PEG derivative (Section S2.7 and Figures S20–S23, Supporting Information). The PEG‐AuNPs had a gold core diameter of 14 ± 1 nm, a hydrodynamic diameter of 23 ± 7 nm, and showed a surface plasmon resonance band with a maximum at 527 nm (Figure S24, Supporting Information).

### Preparation of Photonastic Prototissues

First, proteinosome in oil endowed with a PNIPAM‐based proto‐cortex and PEG‐AuNPs as photothermal transducing proto‐organelles were prepared. For this, in a 1.8 mL vial, 15 µL of an aqueous solution of AMCA‐ or BDP650‐fluorescently labeled azide‐ or BCN‐functionalized BSA/PNIPAM‐*co*‐MAA nanoconjugates (16 mg mL^−1^), 15 µL of fluorescently labeled or non‐labeled polymer (**2**) solution (120 mg mL^−1^) in Na_2_CO_3_ buffer (pH 8.5, 100 mm), 1.6 µL of an aqueous dispersion of PEG‐AuNPs (50 mg mL^−1^), and 13.4 µL of Milli‐Q water were mixed together. Subsequently, 15 µL of polymer (**1**) solution (120 mg mL^−1^) in Na_2_CO_3_ buffer (pH 8.5, 100 mm) were added and mixed thoroughly. Finally, 1 mL of 2‐ethyl‐1‐hexanol was gently added to the aqueous phase at an aqueous/oil volume fraction (*φ_w_*) of 0.06. The mixture was vigorously shaken manually for 15 s to produce a white turbid emulsion. The Pickering emulsion was readily transferred into an Eppendorf tube where it was left to crosslink and sediment for at least 16 h (Sections S3 , Supporting Information).

Photonastic prototissues were prepared using our previously established floating mold technique.^[^ ^8^ ^]^ Briefly, 5 mL of an aqueous solution of TWEEN 80 (5 wt.%) was added to a 47 mm Petri dish, and a PTFE mold was allowed to float at the air/water interface. Equal volumes of sedimented azide‐ and BCN‐functionalized proteinosome emulsions were then thoroughly mixed in an Eppendorf tube. A volume of the 1:1 binary proteinosome mixture was then drop‐casted into the floating PTFE mold to obtain a specific emulsion volume per unit area (see Table S2 , Supporting Information). The prototissue was then allowed to form and transfer into the aqueous solution overnight. Prior to use, the prototissue was detached from the mold, transferred to Milli‐Q water, and left for at least 2 h to wash off the surfactant (Section S4, Supporting Information).

For the preparation of non‐photocontractile prototissues, we used the same procedure with the only difference that we used proteinosomes in oil enclosing a PDMAM‐based proto‐cytoskeleton instead of proteinosomes in oil with a PNIPAM proto‐cortex and PEG‐AuNPs photothermally transducing proto‐organelles. To prepare proteinosomes in oil enclosing a PDMAM‐based proto‐cytoskeleton we used the Pickering emulsion technique and added in sequence to the water phase 15 µL of copolymer (**4**) solution (60 mg mL^−1^) in Na_2_CO_3_ buffer (pH 8.5, 100 mm) and 15 µL of copolymer (**3**) solution (60 mg mL^−1^) in Na_2_CO_3_ buffer (pH 8.5, 100 mm), instead of using copolymers (**1**) and (**2**).

For the enclosure of GOx or AGx enzymes within proteinosomes, we used the method to prepare proteinosomes in oil endowed with a PNIPAM‐based proto‐cortex and PEG‐AuNPs as photothermal transducing proto‐organelles, with the only difference that the water phase was composed by 15 µL of an aqueous solution of non‐labeled azide‐ or BCN‐functionalized BSA/PNIPAM‐*co*‐MAA nanoconjugates (16 mg mL^−1^), 15 µL of non‐labeled polymer (**2**) solution (120 mg mL^−1^) in Na_2_CO_3_ buffer (pH 8.5, 100 mm), 1.6 µL of an aqueous dispersion of PEG‐AuNPs (50 mg mL^−1^), 10 µL of AGx or GOx aqueous solution (50 mg mL^−1^), 3.4 µL of Milli‐Q water, and 15 µL of polymer (**1**) solution (120 mg mL^−1^) in Na_2_CO_3_ buffer (pH 8.5, 100 mm).

## Conflict of Interest

The authors declare no conflict of interest.

## Supporting information



Supporting Information

Supplemental Video 1

Supplemental Video 2

## Data Availability

The data that support the findings of this study are available from the corresponding author upon reasonable request.
