# Supplementary material for: A Photonastic Prototissue Capable of Photo‐Mechano‐Chemical Transduction
Source: Adv Mater. 2025 May 12;37(40):2502830. doi: 10.1002/adma.202502830 (PMC12510286; doi:10.1002/adma.202502830)
Supplement: Supplementary file 1 — Supporting Information [file ADMA-37-2502830-s003.pdf]

ADVANCED MATERIALS

Supporting Information

for *Adv. Mater.*, DOI 10.1002/adma.202502830

A Photonastic Prototissue Capable of Photo-Mechano-Chemical Transduction

*Agostino Galanti, Beatrice Rosetti, Stefano Valente, Nicoletta Braidotti, Maria Sbacchi, Silvia Todros, Piero Pavan and Pierangelo Gobbo**

Supporting information

A Photonastic Prototissue Capable Of Photo-Mechano-Chemical Transduction

*Agostino Galanti,†[a] Beatrice Rosetti, †[a] Stefano Valente,[a] Nicoletta Braidotti,[a] Maria Sbacchi,[a,d] Silvia Todros,[b] Piero Pavan[b,c], and Pierangelo Gobbo[a,d]**

†These Authors contributed equally to the work.

[a]Department of Chemical and Pharmaceutical Sciences, Università degli Studi di Trieste, Via L. Giorgieri, 1, 34127 Trieste, Italy.

[b]Department of Industrial Engineering, Università degli Studi di Padova, Padova, 35131, Italy.

[c]Tissue Engineering Lab, Fondazione Istituto di Ricerca Pediatrica Città della Speranza, Corso Stati Uniti, 4F, 35127 Padova, Italy.

[d]National Interuniversity Consortium of Materials Science and Technology, Unit of Trieste, Via G. Giusti 9, 50121, Firenze (Italy).

S1. Materials and methods **3**

S1.1. Materials 3

S1.2. General methods 3

S1.3. Mold fabrication for prototissues 4

S1.4. Optical microscopy 4

S1.5. Characterization of prototissue thermally- and photo-induced isotropic contraction 5

S1.6. Characterization of photonastic prototissue movement 6

S1.7. Characterization of prototissue mechanical properties 7

S1.7.1. Compression tests 7

S1.7.2. Force relaxation tests 7

S1.8. Activity characterization of enzymatically active prototissues 8

S1.9. Determination of molecular weight cut-off (MWCO) of prototissues 9

S2. Synthesis **10**

S2.1. Synthesis of bio-orthogonal BSA/PNIPAM-co-MAA nanoconjugate 10

S2.2. Synthesis of copolymer (1) 10

S2.3. Synthesis of copolymer (2) 12

S2.3.1. Copolymer (2) fluorescein isothiocyanate labeling 13

S2.3.2. Copolymer (2) rhodamine B isothiocyanate labeling 13

S2.4. AuNPs synthesis 14
S2.5. Synthesis of copolymer (3) 15
S2.6. Synthesis of copolymer (4) 16
S2.7. $PEG_{2000}SH$ synthesis 19
S2.7.1. Synthesis of tosylated PEG derivative (5) 20
S2.7.2. Synthesis of trityl PEG derivative (6) 20
S2.7.3. Synthesis of thiolated PEG derivative (7) 21
S2.8. PEG-AuNPs ligand exchange 22
S2.9. AGx labeling with RITC 23
S2.10. GOx labeling with FITC 23
S3. Proteinosome fabrication: 24
S3.1. General preparation of PEG-diNHS-crosslinked “empty” proteinosomes 24
S3.2. Preparation of proteinosomes enclosing a PNIPAM-based proto-cortex 24
S3.2.a. Preparation of proteinosomes enclosing a PNIPAM-based proto-cortex 24
S3.2.b. Preparation of proteinosomes enclosing a PNIPAM-based proto-cortex and PEG-AuNPs 24
S3.2.c. Preparation of proteinosomes enclosing a PNIPAM-based proto-cortex, PEG-AuNPs, and amyloglucosidase (AGx), or glucose oxidase (GOx) 25
S3.3. Preparation of proteinosomes enclosing a PDMAM-based proto-cytoskeleton 25
S4. Programmed assembly of prototissues 26
S4.1. Assembly of non-patterned and non-layered prototissues 26
S4.2. Programmed assembly of photonastic prototissues 26
S5. Characterization of prototissue mechanical properties 28
S5.1. Young’s modulus characterization discussion 28
S5.2. Discussion of force-relaxation tests on prototissues 28
S6. Numerical analyses on photonastic prototissue 30
S7. Supplementary figures 33
S8. Supplementary videos 46
S9. References 47

S1. Materials and methods

S1.1. Materials

All reagents were purchased from Merck – Sigma Aldrich except *N*-acryloxysuccinimide (Acros Organics), Methacryloxyethyl thiocarbamoyl rhodamine B (Polysciences), aminomethyl coumarin NHS ester (AMCA®) and BODIPY 650/665-X NHS ester (BDP650®) (Lumiprobe). Unless otherwise specified, all reagents were used without further purification. The enzymes used: amyloglucosidase (AGx – from Aspergillus niger, ~ 120 KU g^{-1}) and glucose oxidase (GOx – from Aspergillus niger, 100-150 KU g^{-1}) were purchased from Merck – Sigma Aldrich. Ultrapure water (resistivity > 18 MΩ cm) was produced using a Millipore water purification system. Dialysis tubing with MWCO ~ 12-14 kDa were purchased from Spectrum Labs. Centrifugal filter units with filter cartridge MWCO ~ 50 kDa were purchased from Merck – Sigma Aldrich.

S1.2. General methods

The NMR spectra were recorded on a Varian 400 spectrometer (^{1}H: 400 MHz, ^{13}C: 100.5 MHz). The chemical shift (δ) for ^{1}H and ^{13}C are given in ppm relative to signals of the residual solvents ($CHCl_3$ at 7.26 ppm ^{1}H-NMR, $CDCl_3$ at 77.16 ppm ^{13}C-NMR, or H_2O at 4.79 ppm ^{1}H-NMR).

Polymers were characterized by means of tetra-detection gel permeation chromatography (TD-GPC) using an Omnisec RESOLVE/REVEAL system (Malvern Panalytical) equipped with refractive index (RI), UV-vis, right angle light scattering (RALS)/low angle light scattering (LALS), and differential viscometer detectors. All data were collected and processed using Omnisec v12 software. The multi-detector system was calibrated using PolyCAL™ Pullulan (narrow distribution – calibration, 107 K) and dextran (broad distribution – verification, 68 K) standards (Malvern Panalytical). All the mobile phases used were pre-filtered through a bottle vacuum filter funnel with a 0.22 µm PES membrane (Steritop®, Merck). For chromatographic separation of the commercial polysaccharides FITC-dextrans and dextrin, two columns (A6000M and A2500, Malvern Panalytical) were employed with 0.1 M PBS: MeOH = 9.5:0.5 mobile phase at a 0.7 mL min^{-1} flow rate (column and detector oven kept at 20 °C, whereas autosampler at 8 °C). Samples were prepared at a concentration of *ca.* 1.5 mg mL^{-1}, dissolved in the eluent, and filtered through 0.22 µm Nylon syringe filters. For the analysis, a *dn/dC* of 0.148 mL g^{-1} was used. Results are the average of three separate runs.
On the other hand, for chromatographic separation of synthetic copolymers (1-4), two cationic columns (TSKgel G6000PWXL-CP + G3000PWXL-CP, Tosoh Bioscience) were employed with 0.1 M $NaNO_3$ at pH 2.6, acidified with acetic acid (*ca.* 0.5% v/v) mobile phase at a 0.5 mL min^{-1} flow rate (column and detector oven kept at 20 °C, whereas autosampler at 4 °C). Polymer samples were prepared at a concentration of *ca.* 3 mg mL^{-1}, dissolved in the eluent, and filtered through 0.45 µm regenerated cellulose syringe filters. For both P(NIPAM)-based copolymers (1) and (2), the *dn/dC* used was 0.149 ± 0.002 mL g^{-1}, calculated for a similar P(NIPAM)-based copolymer (poly(*N*-isopropylacrylamide-*co*-methacrylic acid) under the same chromatographic conditions. For P(DMAM)-based copolymers, the *dn/dC* was experimentally determined and corresponded to 0.158 ± 0.002 mL g^{-1} (3) and 0.142 ± 0.011 mL g^{-1} (4). Results were the average of three separate runs.
Transmission electron microscopy images were acquired using an EM 208 Electron microscope (Philips) operating at 100 kV and equipped with a 11 MP digital camera (Olympus Quemesa). The samples were prepared by depositing AuNP aqueous dispersions on continuous carbon-coated 200 mesh Cu grids (Electron Microscopy Supplies).

Dynamic light scattering and ζ-potential measurements were acquired using a Z Sizer Nano instrument in capillary electrode cells DTS1070 (Malvern) in buffered phosphate solution (PBS) 5 mM, pH 6.8. UV-vis absorption/transmittance spectra were measured using a double beam UV-vis-NIR spectrophotometer Cary 5000 (Agilent) inside matched Suprasil® quartz cuvettes with 1 cm optical path length. Transmittance spectra of PCMs were obtained by placing a PCM on a drop of water on a glass coverslip (thickness: 145 µm), and

subsequent PCM adhesion by water removal. Transmittance was measured against air (reference beam), by placing the as-prepared sample perpendicular to the analysis beam.

The thermoresponsive properties of polymers in solution were characterized by monitoring the transmittance of stirred 2 mg mL^{-1} polymer solutions in Milli-Q water using an optical fiber (400 µm diameter) UV-vis spectrophotometer Flame (Ocean Optics) equipped with a DH-Mini light source (Ocean Optics) and a 1 cm cuvette holder CUV (Ocean Optics) in-house modified to include a Peltier temperature control system (Thorlabs) in 1 cm Suprasil® quartz cuvettes (Hellma). The internal temperature was monitored with a K type thermocouple (RS Components) inside the cuvette and a temperature data logger (Lascar Electronics). LCST was determined by measuring the transmittance at 550 nm as the temperature at which transmittance corresponds to 50%. The thermoresponsive properties of hydrogels were characterized with the same setup, by immersing in a quartz cuvette filled with 3 mL of water a 2 mm-thick, 7x18 mm hydrogel sample prepared in a custom-made PMMA holder sandwiched between two glass coverslips (thickness: 0.17 mm). The sample holder was arranged in the cuvette in order to maintain the hydrogel perpendicular to the analysis beam.

S1.3. Mold fabrication for prototissues

The poly(tetrafluoroethylene) (PTFE) molds for PCM preparation were fabricated by cutting a 0.2 mm thick PTFE sheet using a numerical control (CNC) laser cutting machine equipped with a 60 W CO_2 laser (OMTech). The following mold geometries were made and used in this work:

- 4x4 circle array: circle diameter = 2 mm, distance between circles = 3 mm (Supplementary Figure S1a);
- 2x2 circle array: circle diameter = 5 mm, distance between circles = 7.5 mm (Supplementary Figure S1b);
- 2x2 rectangle array: 2.5 x 5 mm sides (Supplementary Figure S1c);
- “Starfish” mold (Supplementary Figure S1d).

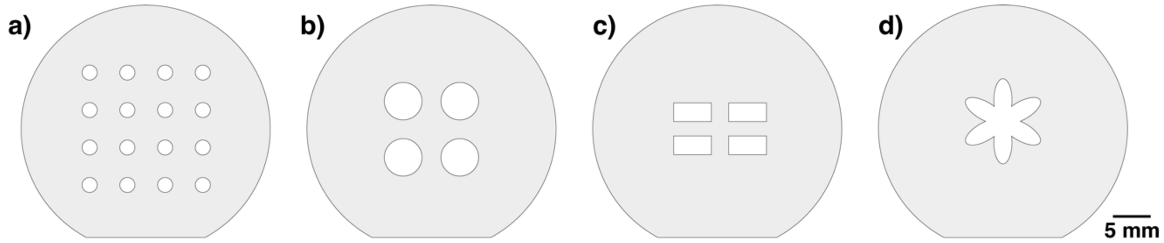

Figure S1. Scheme illustrating the mold geometries used in this study.

S1.4. Optical microscopy

Proteinosomes and PCMs were characterized by confocal fluorescence microscopy imaging using a FV3000 confocal laser scanning microscope (Evident). The system is equipped with 5 excitation laser lines: 375, 405, 488, 561 and 640 nm (Coherent), a galvanometric scanning head and 4 spectral detectors allowing simultaneous imaging of up to 4 fluorescence channels. Brightfield images were acquired using a scanning laser line and a transmitted light detector. The IX 83 inverted microscope body (Olympus) includes a motorized sample stage (Prior Scientific) for multi-area mosaic image acquisition. The objective lenses used were the following: UPLXAPO 4x/0.16 NA (Olympus), UPLXAPO 10x/0.4 NA (Olympus), UPLXAPO 20x/0.8 NA (Olympus), a silicone oil immersion UPLSAPOXS 30x/1.05 NA (Olympus) and an oil immersion lens UPLXAPOO 60x/1.42 NA (Olympus). Analysis of Pickering emulsions’ size distribution was performed by threshold-based

segmentation with the software suite Zen (Zeiss); equivalent diameter and roundness were extracted as parameters defining the emulsions' size and morphology. For Z stacks, deconvolution was performed by applying the Advanced nearest neighbor image deconvolution algorithm provided with the CellSens software suite (Olympus). Controlled temperature experiments were performed in a custom-built stage equipped with a Peltier temperature control system (Thorlabs – see Supplementary Figure S2) having a 0.17 mm-thick bottom glass window. The internal temperature was monitored with a K type thermocouple (RS) and a temperature data logger (Lascar Electronics).

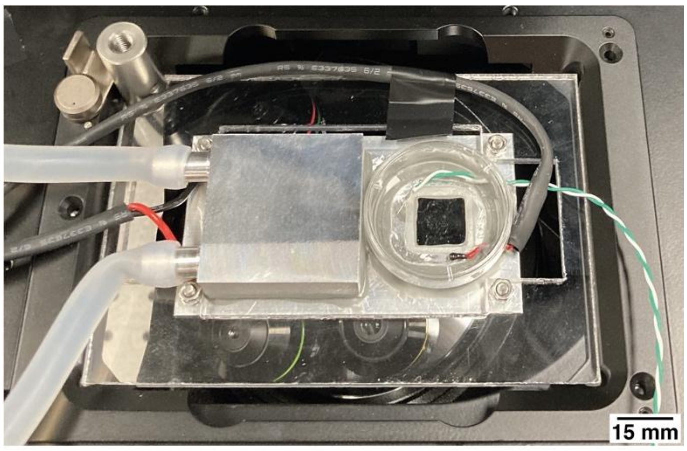

Figure S2. Photograph of the custom-built temperature-controlled stage for confocal fluorescence microscopy.

S1.5. Characterization of prototissue thermally- and photo-induced isotropic contraction

The thermoresponsive properties and light-induced contraction of isotropic PCMs were characterized using a setup consisting of a brightfield USB microscope AF7515MZT (DinoLite) and a home-made thermostated sample chamber (35.5 mm diameter) with a Peltier temperature control system (Thorlabs – see Supplementary Figure S3). The sample chamber was filled with 3 mL of Milli-Q water. The internal temperature was monitored with a K type thermocouple (RS) placed at the same height of the water level, on which a floating PCM was carefully laid. The temperature data was recorded using a data logger (Lascar Electronics). Light-induced PCM contraction was obtained by irradiating the samples with a high-intensity green LED (Solis 525C, Thorlabs – λ ≈ 520/20 nm, DC 2200 LED driver) coupled with a Ø2", f = 175 mm plano-convex lens. The lens-sample distance was set at 13 cm to obtain a homogeneous irradiation beam, perpendicular to the sample, having a circular section of 27 mm diameter. The LED irradiance was determined using a calibrated spectrometer equipped with a cosine corrector (Avantes – see Supplementary Figure S4). The PCM isotropic contraction was determined by brightfield microscopy imaging and image analysis to determine PCM area and it was defined as a fraction of the area of the contracted material (A_c) vs. material initial area (A_i) following relaxation (see Supplementary Equation S1). For thermally-induced contraction, the A_i value used corresponded to the PCM area at 18 °C. For photo-induced contraction, A_i value corresponded to the PCM area at 25 °C with light OFF. PCM area was determined either manually (ImageJ software), or by using an automated image analysis software, Python code available at: https://github.com/PierangeloGobbo1986/LAMPAD.

Equation S1.

$$Contraction\ (area\ \%) = \left(1 - \frac{A_c}{A_i}\right) * 100$$

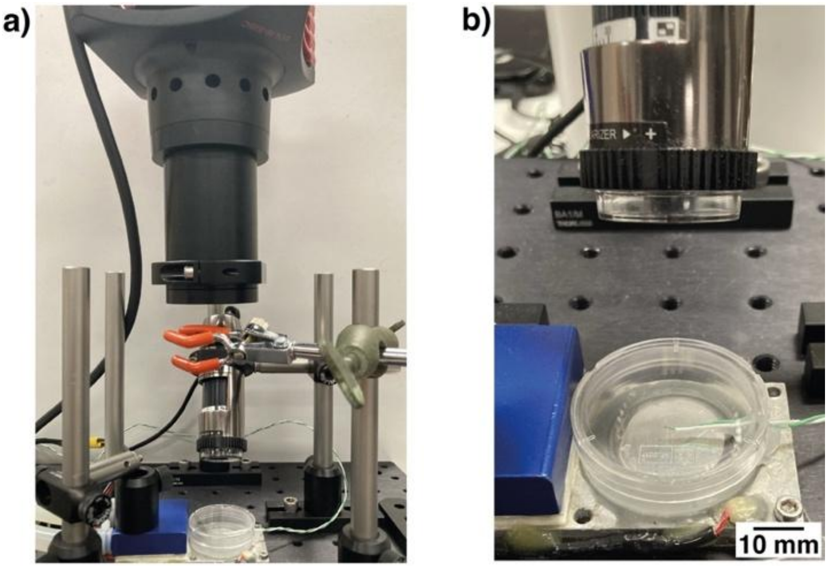

Figure S3. Photographs of the custom-built setup used for characterizing the thermoresponsive properties and light-induced contraction of isotropic PCMs. a) Photograph of the whole setup including high intensity LED (top). b) Photograph of the thermostated sample chamber and brightfield USB microscope.

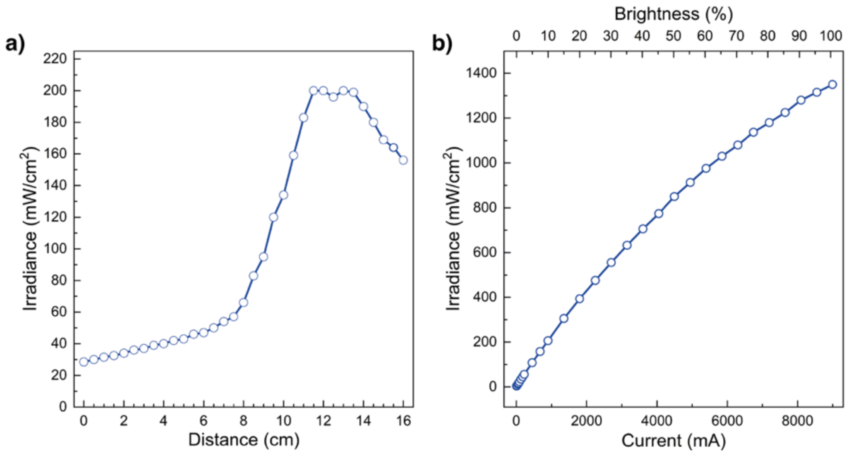

Figure S4. Characterization of the LED irradiance in the experimental setup. a) Variation of the irradiance of LED set at 10% brightness upon changing the LED-sample distance. b) Variation of the irradiance upon changing the LED brightness / drive current.

S1.6. Characterization of photonastic prototissue movement

Light-induced movements on photonastic six-armed starfish prototissues were studied at 25 °C with a custom-made setup consisting of a Peltier temperature controller and a sample chamber filled with 3 mL of Milli-Q water that allows for the observation of the material from top and side transparent windows. Imaging was performed with a brightfield USB microscope AF7515MZT (DinoLite) (see Supplementary Figure S5). In order to prevent the detachment of the prototissue from the base upon irradiation, the sample was fixed in its center with a needle. The sample was irradiated from the top window, perpendicularly to the sample, using the same high-intensity green LED described above, at an irradiance of 1.35 W cm^{-2}. The characterization of the PCM movement was done by taking a time-lapse video from the side window, and by determination of the curvature, defined as the reciprocal of the radius of the circumference circumscribed to the PCM. The analysis was performed on ImageJ software, using the *circumcircle* script (https://bitbucket.org/davemason/threepointcircumcircle/src/master/), finding the circumscribed circumference from a triangle which vertices were manually selected on each image.

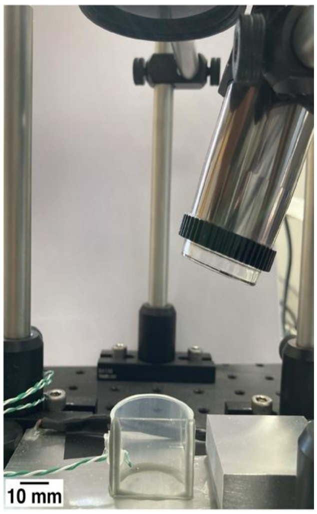

Figure S5. Photograph of a custom-built setup used for characterizing the photo-induced movements of anisotropic PCM samples.

S1.7. Characterization of prototissue mechanical properties

The mechanical properties of PCMs were characterized using the micro-/nano-indenter FT-MTA03 (Femto Tools AG – Buchs, Switzerland) already described previously.[1, 2] Glass microspheres (diameter 250-300 mm, BSGMS-2.2 from Cospheric) were glued to the silicon probe using Norland Optical Adhesive 81 (Norland Products) cured with a 365 nm UV LED (Thorlabs). Samples were immobilized on the surface of bi-adhesive tape at the bottom of a polystyrene petri dish filled with Milli-Q water.

The indenter was enclosed in a custom-built thermostated box (DELTA Instruments, range of temperatures supported: room temperature to 45°C) that allowed temperature-controlled experiments to be carried out.

Two different types of experiments were performed in triplicate on each sample: compression tests, for the evaluation of the Young's modulus (*E*), and force relaxation tests, for the characterization of the viscoelastic properties.

S1.7.1. Compression tests

Samples were indented at 10 μm s^{-1} using the stick-slip actuator with a step size of 2 μm. The maximal indentation force was adjusted based on sample stiffness in order to avoid over-indentation, and never exceeded 30 μN. Indentation depth was kept below 10% of the sample thickness. On each sample 9 curves were acquired by indenting in a 3 x 3 array (1000 μm x 1000 μm with 500 µm spacing). Force-displacement curves were processed for the Young's modulus evaluation using our automated indentation analysis software ALIAS (code available at: *https://github.com/PierangeloGobbo1986/ALIAS*).[1] Data were fitted using the Johnson-Kendall-Roberts (JKR) model.

S1.7.2. Force relaxation tests

Samples were indented of 40 μm at 25 μm s^{-1} speed, and the displacement was maintained for 10 s while continuously recording the force. Subsequently, the probe was retracted from the sample. On each sample 9 curves were acquired by indenting in a 3 x 3 array (1000 μm x 1000 μm with 500 µm spacing). Data was processed by using our ALIAS Viscoelasticity software (Python, https://github.com/ PierangeloGobbo1986/ALIAS-Viscoelasticity).[2] Firstly, the recorded force, *F*(*t*) was converted into the relaxation modulus, *E(t)*, by using Hertz's indentation theory[3] considering the Poisson's ratio (ν) to be 0.5. Subsequently, the Generalized Maxwell Model, consisting of three Maxwell elements in parallel with a single spring,[4] was used to fit the data (Supplementary Equation S2), where *R* is the radius of the spherical probe, δ is the indentation depth, E_∞ is the equilibrium modulus representing the elastic modulus of the linear spring and measuring the material response after complete viscous relaxation; E_i and τ_i are respectively the modulus of the spring and the characteristic relaxation time of the ith Maxwell element. In addition, *n* is the number of spring-dashpot series, which in our case is *n=3*.

Equation S2. $$E(t) = \frac{3}{4}\frac{(1-\nu^2)\,F(t)}{\delta^{\frac{3}{2}}\sqrt{R}},\ E(t) = E_\infty + \sum_{i=1}^{n} E_i e^{\frac{-t}{\tau_i}}$$

This analysis provided information on instantaneous (E_0) and equilibrium (E_∞) moduli relative to t = 0 and t = ∞ respectively, while by applying the Fourier Transform on time-dependent relaxation modulus,[5] the complex modulus, $E^*(\omega)$ (Supplementary Equation S3), was obtained providing information on storage, $E'(\omega)$ (Supplementary Equation S4), and loss modulus, $E''(\omega)$ (Supplementary Equation S5), as function of frequency (ω):

Equation S3. $$E^{*}(\omega) = E'(\omega) + iE''(\omega)$$

Equation S4. $$E'(\omega) = E_\infty + \sum_{i=1}^{n} E_i \frac{\omega^2\tau_i^2}{1+\omega^2\tau_i^2}$$

Equation S5. $$E''(\omega) = \sum_{i=1}^{n} E_i \frac{\omega\tau_i}{1+\omega^2\tau_i^2}$$

S1.8. Activity characterization of enzymatically active prototissues

Time-dependent pH measurements were performed at 25 °C under continuous stirring using a data logging Seven Compact digital pH-meter with an InLab Micro electrode (Mettler Toledo). In order to overcome pH drift due to temperature variations in the buffered solution following light irradiation, the reaction was performed in a chamber (acrylic, diameter: 13.5 mm, height: 18 mm) equipped with a Peltier control system (Thorlabs) capable of actively overcoming the temperature variations and to keep the solution at 25 °C regardless of light irradiation (see Supplementary Figure S6a). In order to prevent pH drift in the absence of enzymatic activity, but still to be able to appreciate a pH variation in the presence of the enzymatically active PCM, we filled the measurement chamber with 1.9 mL of a weak phosphate buffer (1 mM), with an initial pH ~ 6.2. The PCM was inserted and kept floating in the chamber, but separated from the glass electrode inside a custom-built well with a perforated base (acrylic, square section, side: 6.5 mm, height: 14 mm, see Supplementary Figure S6b) allowing for the exchange of liquid, but preventing the PCM from being dragged by the flow created by the stirring bar, or touching the electrode. After adding the PCM inside the measurement chamber, we waited 120 s for thermal equilibration, then added 12.6 µL of the substrate solution (dextrin, 80 mg mL^{-1} in phosphate buffer 3.5 mM). Data logging was started at the same moment as the substrate solution was added. For photo-mechano-chemical transduction experiments, the sample was irradiated for 180 s from the top using the same high-intensity green LED described above, at an irradiance of 1.35 W cm^{-2} to achieve the fully contracted state before adding the substrate for the enzyme cascade reaction.

Control experiments of AGx/GOx enzyme cascade reaction in absence of PCM structure were performed by dissolving the enzymes in bulk aqueous phosphate buffer (1 mM) and experiments were performed identically as on PCMs. AGx and GOx were dissolved in aqueous buffer each at a concentration of 17 µg mL^{-1}, which corresponded to adding 32 µg of each enzyme in solution. This quantity was approximately equal to the quantity of each enzyme encapsulated within a PCM (composition: Supplementary Table S2, Entry 5). We estimated the amount of enzyme within a PCM by calculating its volume assuming PCM thickness would be equal to 0.1 mm, which for a 5 mm diameter disk equals to 8 µL.

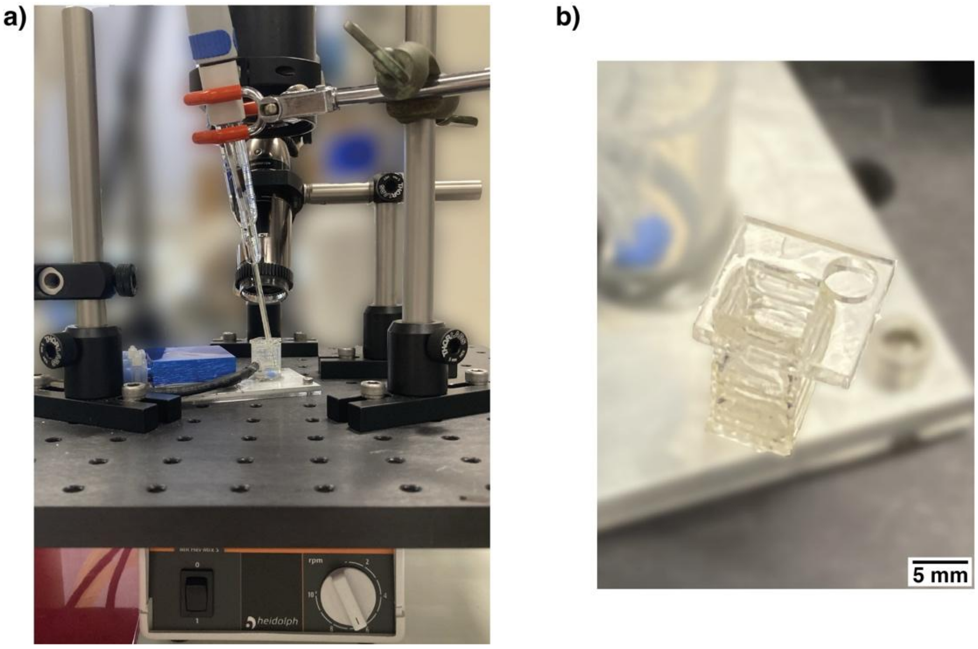

Figure S6. Photographs of the custom-built temperature-controlled setup to perform time-dependent pH measurements under high intensity light irradiation. a) Picture of the whole setup including a pH glass electrode inserted in the temperature-controlled chamber. b) Picture of the custom-built well used to separate the PCM from the glass electrode. To perform the measurement, the well was inserted in the temperature-controlled chamber in (a), the PCM was allowed to float in the square well, while the electrode was inserted in the round-shaped opening.

S1.9. Determination of molecular weight cut-off (MWCO) of prototissues

MWCO of prototissues was determined by performing permeation experiments of FITC-labeled dextran with different known molecular weights (4, 10, 20, 40, 70, and 150 x10^{-3} g mol^{-1} – characterization: Supplementary Table S3) through PCMs. The contractile PCMs used for these experiments were prepared without PEG-AuNPs in order to prevent FITC fluorescence absorption/quenching (See Supplementary Table S2 – Entry 2), but always using 0.64 µL mm^{-2} emulsion volume per unit area in order to obtain the same PCM thickness as the enzymatically active PCMs. Each experiment was performed on a freshly prepared circular prototissue with 2 mm diameter without detaching it from the PTFE mold. The microscope temperature-controlled chamber (see Supplementary Figure S2) was filled with 1 mL of Milli-Q water, then the PTFE mold with the PCM was allowed to float on the liquid and the sample was left for 5 minutes to thermally equilibrate. Subsequently, 2 µL of 1 mg mL^{-1} solutions of FITC-labeled dextrans with different molecular weights were drop-casted on top of the floating PCM. FITC-labeled dextran permeation was monitored by confocal fluorescence microscopy by Z-stack imaging of the whole depth of the sample (see Supplementary Figure S52). Image analysis was performed by measuring the Z-axis fluorescence intensity profile curves for the blue and green channels which correspond to the AMCA-labeled protocell membranes (representing therefore the prototissue), and the FITC-labeled dextran, respectively (see Supplementary Figure S53). The position of the FITC-labeled dextran with respect to the prototissue was defined as the difference between the maximum of the curve related to the FITC-dextran along the Z axis and the maximum of the curve related to the prototissue, which was set to 0 µm distance (main text Figure 4f). We assigned full permeation when the maximum of the fluorescence intensity curve relative to FITC-dextran had a negative value of distance compared to the position of the prototissue. This meant that FITC-dextran permeated through the PCM and diffused below it into the bulk aqueous solution. In contrast, no permeation was attributed when the position of the maximum of the FITC-dextran green fluorescence intensity curve had a positive value of distance compared to the position of the prototissue. This meant that FITC-dextran remained above the prototissue.

S2. Synthesis

S2.1. Synthesis of bio-orthogonal BSA/PNIPAM-co-MAA nanoconjugate

Aminomethyl coumarin (AMCA) NHS ester or BODIPY 650/665 X (BDP 650) NHS ester-labeled, and non-labeled azide- and BCN-functionalized BSA/PNIPAM-*co*-MAA nanoconjugates were synthesized and characterized according to the general procedure established previously.[6]

S2.2. Synthesis of copolymer (1)

Poly(*N*-isopropylacrylamide-*co*-*N*-acryloxy succinimide) (1) was synthesized using a RAFT polymerization technique, adapting a previously established procedure.[6] *N*-isopropylacrylamide (NIPAM) and α,α,'-azoisobutyronitrile (AIBN) were freshly recrystallized from hexane and methanol, respectively. NIPAM (927.1 mg, 8.2 mmol, χ = 0.95), *N*-acryloxy succinimide (AANHS, 72.9 mg, 0.43 mmol, χ = 0.05), AIBN (0.33 mg, 2.0 µmol) and 2-cyano-2-propyl dodecyl trithiocarbonate as RAFT agent (CPDTC, 3.5 mg, 10.0 µmol) were dissolved in acetonitrile (4 mL) in a Schlenk tube equipped with a stirrer bar, adding DMF (300 µL) as internal standard to assess the monomer conversion via ^{1}H-NMR. The solution was purged from oxygen using a freeze-pump-thaw technique and the Schlenk tube was filled with argon and sealed. The polymerization was carried out at 65 °C for 3.5 days under stirring. The polymer was isolated by precipitation from hexane/Et_2O (1:1, 80 mL) as a crystalline light-yellow powder (905.0 mg, 91%). M_n = 95,000 g mol^{-1} (^{1}H-NMR), Conversion = 96% (^{1}H-NMR), 3% mol AANHS (^{1}H-NMR).

^{1}H-NMR (Supplementary Figure S7, 400 MHz, $CDCl_3$): δ (ppm) = 7.13-5.72 (1H, -N**H**-CH(CH_3)$_2$, NIPAM), 4.11-3.87 (1H, -NH-C**H**(CH_3)$_2$, NIPAM), 2.93-2.80 (0.13H, -(C**H$_2$**-C**H$_2$**)-, AANHS), 2.72-2.54 (0.33H, -[C**H**-CH_2]$_n$-, AANHS), 2.46-1.94 (1H, -[C**H**-CH_2]$_n$-, NIPAM), 1.94-1.45 (2H, -[CH-C**H$_2$**]$_n$-, NIPAM + -[CH-C**H$_2$**]$_n$-, AANHS), 1.23-0.93 (6H, -NH-CH(C**H$_3$**)$_2$, NIPAM).

^{13}C-NMR (Supplementary Figure S8, 100.5 MHz, $CDCl_3$): δ (ppm) = 174.4 (**C**=O, NIPAM), 170.4 (N**C**=O, AANHS), 42.6 (-[**C**H-CH_2]$_n$-, NIPAM), 41.5 (-N**C**H(CH_3)$_2$, NIPAM), 36.6 (-[CH-C**H$_2$**]$_n$-, NIPAM + -[**C**H-CH_2]$_n$-, AANHS), 35.1 (-[CH-**C**H$_2$]$_n$-, AANHS), 25.9 (-**C**H$_2$-**C**H$_2$-, AANHS), 22.7 (-NCH(**C**H$_3$)$_2$, NIPAM).

TD-GPC: *dn/dC* = 0.149 ± 0.002 mL g^{-1}; M_n = 125.2 ± 0.8 x 10^3 g mol^{-1}, M_w = 132 ± 1 x 10^3 g mol^{-1}, $Đ$ = 1.05 ± 0.01. Values calculated by the average of three separate measurements, error is expressed as the standard deviation.

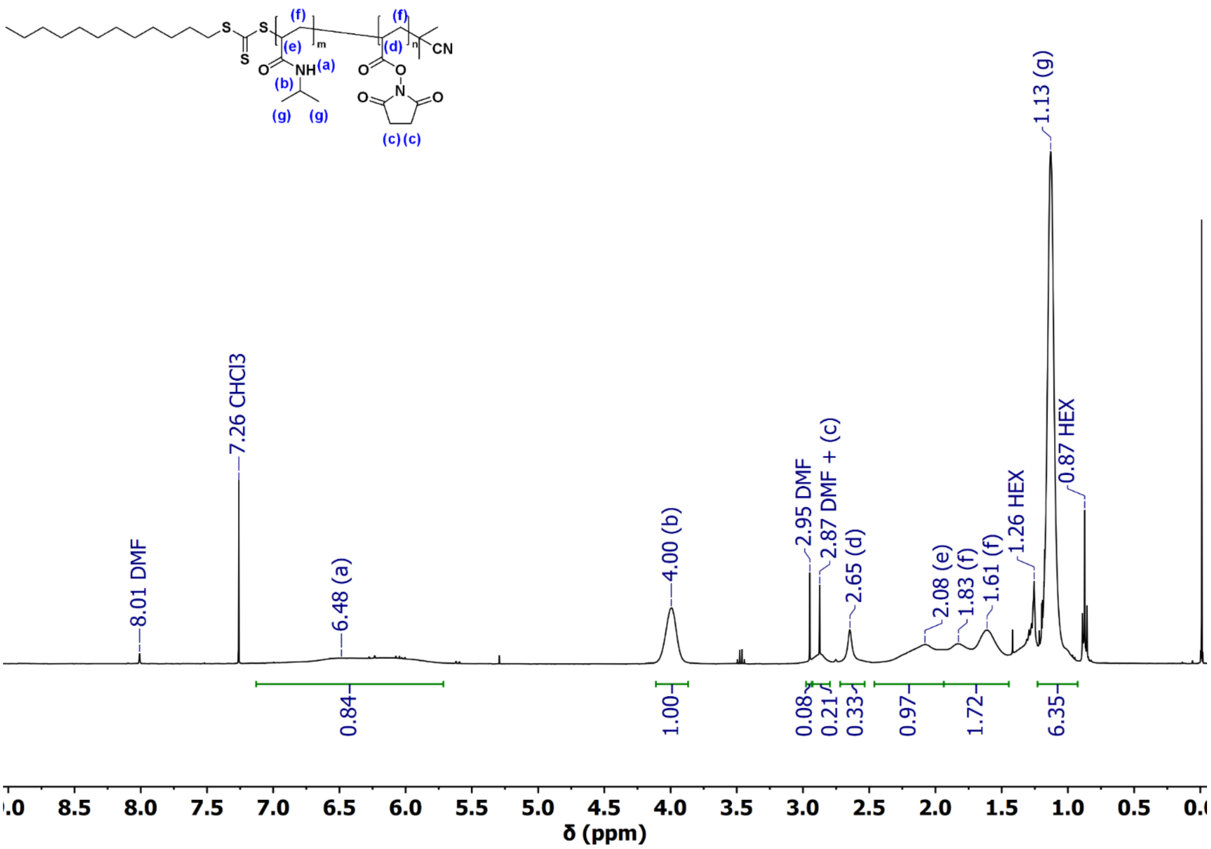

Figure S7. ^{1}H-NMR (400 MHz, $CDCl_3$) spectrum of copolymer (1), recorded in $CDCl_3$ and referenced against the peak of residual $CHCl_3$ at 7.26 ppm.

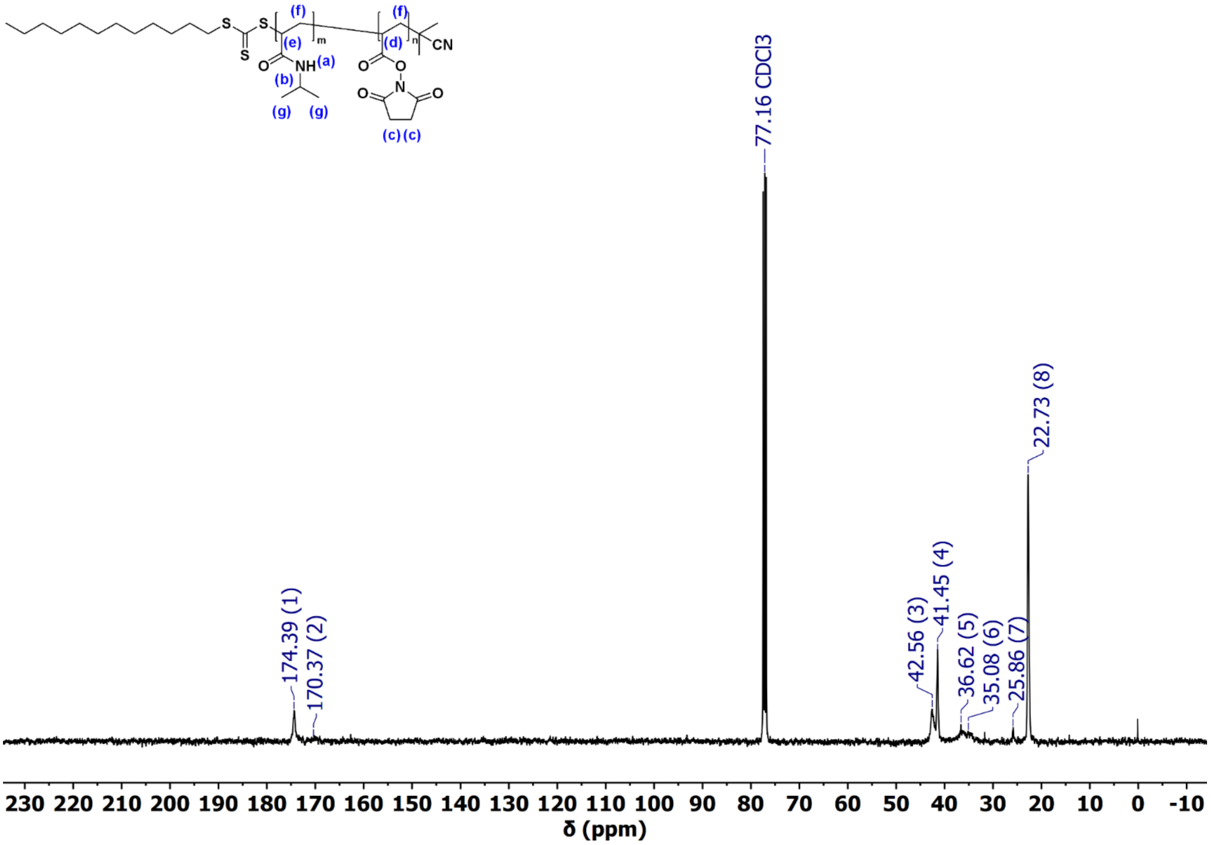

Figure S8. ^{13}C-NMR (100.5 MHz, $CDCl_3$) spectrum of copolymer (1), recorded in $CDCl_3$ and referenced against the peaks of $CDCl_3$ at 77.16 ppm.

S2.3. Synthesis of copolymer (2)

Poly(*N*-isopropylacrylamide-*co*-aminoethylmethacrylate) (2) was synthezised using a RAFT polymerization technique, adapting a previously established procedure.[6] *N*-isopropylacrylamide (NIPAM) and α,α,'-azoisobutyronitrile (AIBN) were freshly recrystallized from hexane and methanol, respectively. NIPAM (971.0 mg, 8.6 mmol, χ = 0.98), 2-Aminoethyl methacrylate hydrochloride (AEMA, 29.0 mg, 175 µmol, χ = 0.02), AIBN (0.33 mg, 2.0 µmol) and 2-cyano-2-propyl dodecyl trithiocarbonate as RAFT agent (CPDTC, 3.5 mg, 10.0 µmol) were dissolved in acetonitrile (4 mL) in a Schlenk tube equipped with a stirrer bar, adding DMF (300 µL) as internal standard to assess the monomer conversion via ^{1}H-NMR. The solution was purged from oxygen using a freeze-pump-thaw technique and the Schlenk tube was filled with argon and sealed. The polymerization was carried out at 65 °C for 5 days under stirring. The polymer was isolated by precipitation from hexane/Et_2O (1:1, 90 mL) as a crystalline light-yellow powder (439.8 mg, 44%). M_n = 37,500 g mol^{-1} (^{1}H-NMR), Conversion = 37.5% (^{1}H-NMR), 2% mol AEMA (^{1}H-NMR).

^{1}H-NMR (Supplementary Figure S9, 400 MHz, D_2O): δ (ppm) = 8.08-7.35 (0.43H, -N$\underline{\mathbf{H}}$, NIPAM), 4.39-4.11 (0.05H, -O-C$\underline{\mathbf{H_2}}$-CH_2-NH_2, AEMA), 4.10-3.66 (1H, -NH-C$\underline{\mathbf{H}}$(CH_3)$_2$, NIPAM), 3.44-3.30 (0.07H, -O-CH_2-C$\underline{\mathbf{H_2}}$-NH_2, AEMA), 2.36-1.85 (1H, -[C$\underline{\mathbf{H}}$-CH_2]$_n$-, NIPAM) , 1.85-1.31 (2H, -[CH-C$\underline{\mathbf{H_2}}$]$_n$-, NIPAM + -[C(CH_3)-C$\underline{\mathbf{H_2}}$]$_n$-, AEMA), 1.31-1.25 + 1.04-0.92 (0.16H + 0.17H, -[C(C$\underline{\mathbf{H_3}}$)-CH_2]$_n$-, AEMA), 1.25-1.04 (6H, -NH-CH(C$\underline{\mathbf{H_3}}$)$_2$, NIPAM).

^{13}C-NMR (Supplementary Figure S10, 100.5 MHz, D_2O): δ (ppm) = 175.0 ($\underline{\mathbf{C}}$=O), 41.7 (-NH-$\underline{\mathbf{C}}$H(CH_3)$_2$), 34.6 (-[$\underline{\mathbf{C}}$H-$\underline{\mathbf{C}}$H$_2$]$_n$-), 21.5 (-NH-CH($\underline{\mathbf{C}}$H$_3$)$_2$).

TD-GPC: *dn/dC* = 0.149 ± 0.002 mL g^{-1}; M_n = 65.3 ± 0.6 x 10^3 g mol^{-1}, M_w = 74.2 ± 0.3 x 10^3 g mol^{-1}, $Đ$ = 1.14 ± 0.01. Values calculated by the average of three separate measurements, error is expressed as the standard deviation.

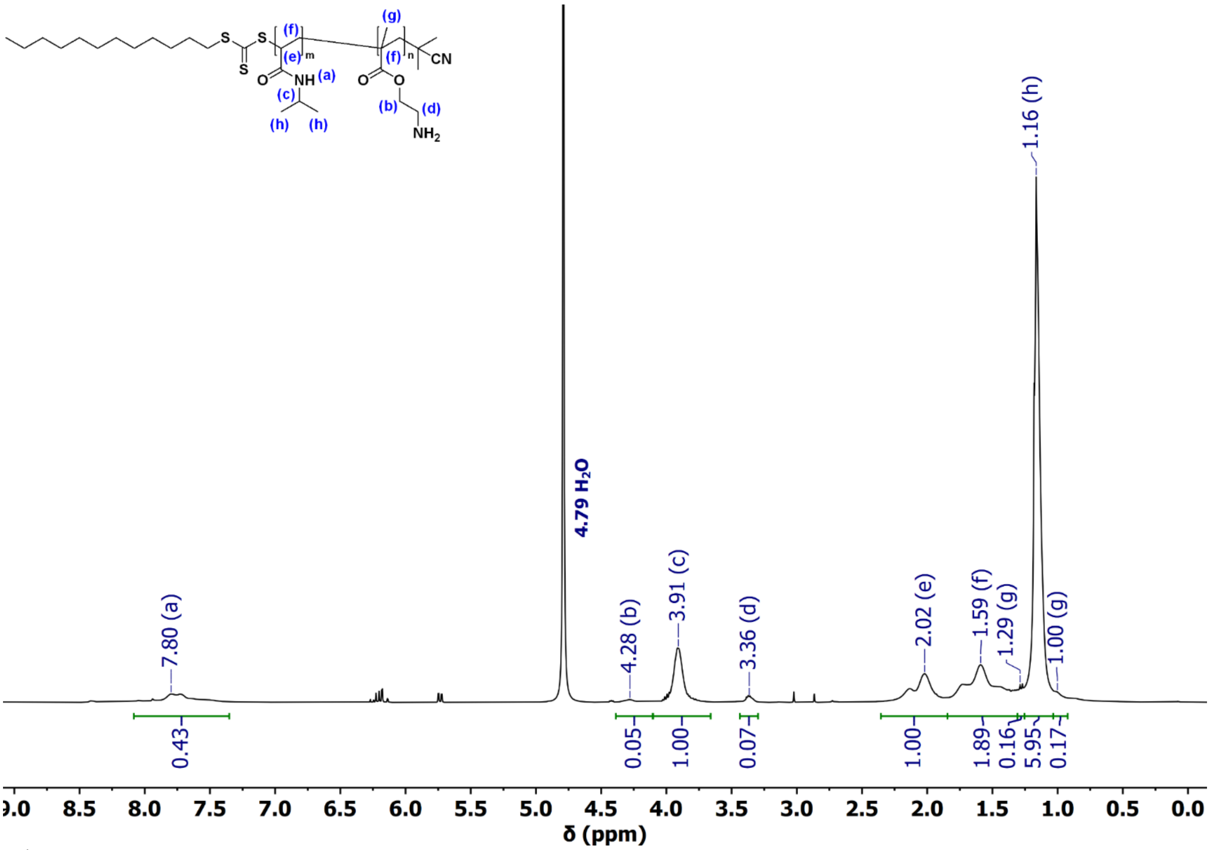

Figure S9. ^{1}H-NMR (400 MHz, D_2O) spectrum of copolymer (2), recorded in D_2O and referenced against the peak at 4.79 ppm of H_2O.

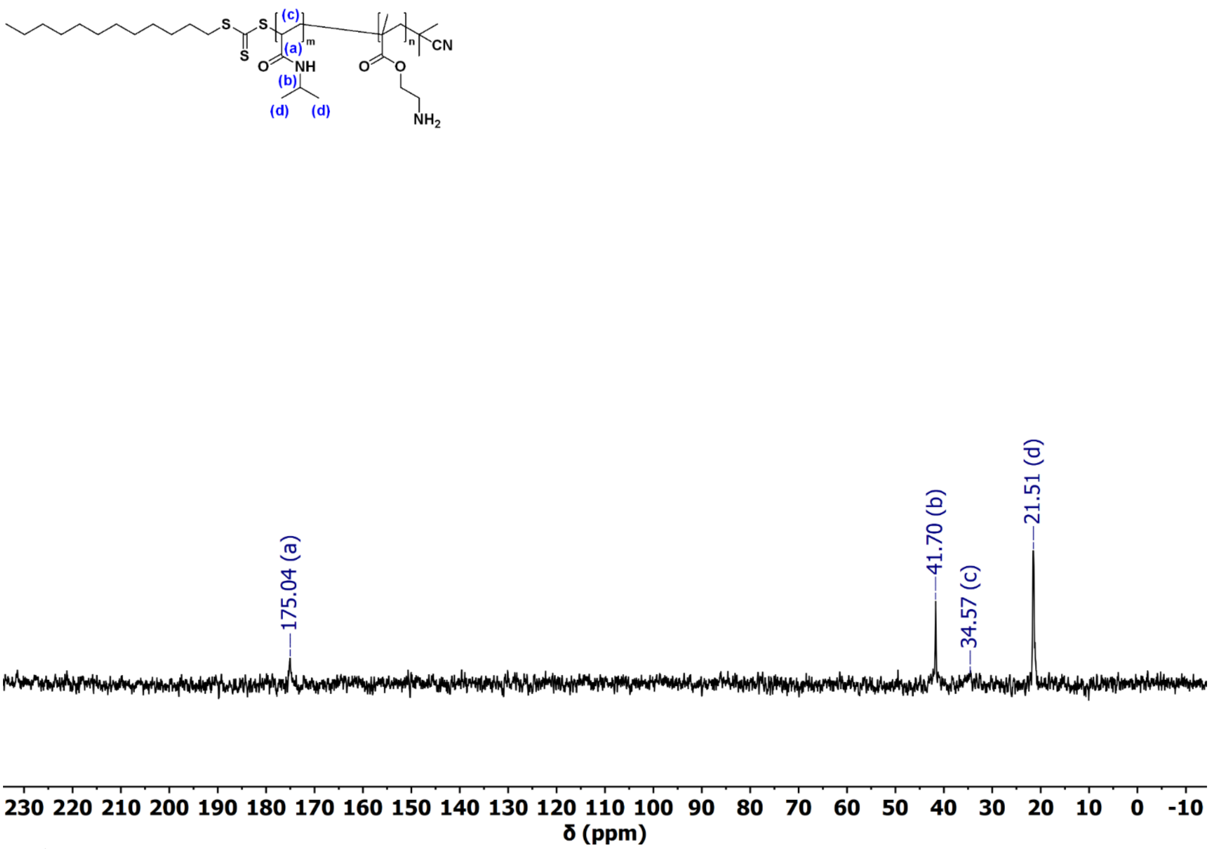

Figure S10. ^{13}C-NMR (100.5 MHz, D_2O) spectrum of copolymer (2), recorded in D_2O.

S2.3.1. Copolymer (2) fluorescein isothiocyanate labeling

Copolymer (2) was dissolved in $Na_2CO_3/NaHCO_3$ (0.1 M, pH ≈ 8.5) buffer (20 mg, 2.7 mg mL^{-1}). A fluorescein isothiocyanate (FITC, 1 mg mL^{-1} in DMSO) solution was added dropwise to the stirring polymer solution. The solution was stirred for 2 h at room temperature, then purified by dialysis (12-14 kDa MWCO membranes) for 24 h, and lyophilized. The degree of labeling was determined via UV-vis absorption spectroscopy to be equal to: 0.01 (Supplementary Figure S11a).

S2.3.2. Copolymer (2) rhodamine B isothiocyanate labeling

Copolymer (2) was dissolved in $Na_2CO_3/NaHCO_3$ (0.1 M, pH ≈ 8.5) buffer (20 mg, 2.7 mg mL^{-1}). A rhodamine B isothiocyanate (RITC, 1 mg mL^{-1} in DMSO) solution was added dropwise to the stirring polymer solution. The solution was stirred for 2 h at room temperature, then purified by dialysis (12-14 kDa MWCO membranes) for 24 h and lyophilized. The degree of labeling was determined via UV-vis absorption spectroscopy to be equal to: 0.002 (Supplementary Figure S11b).

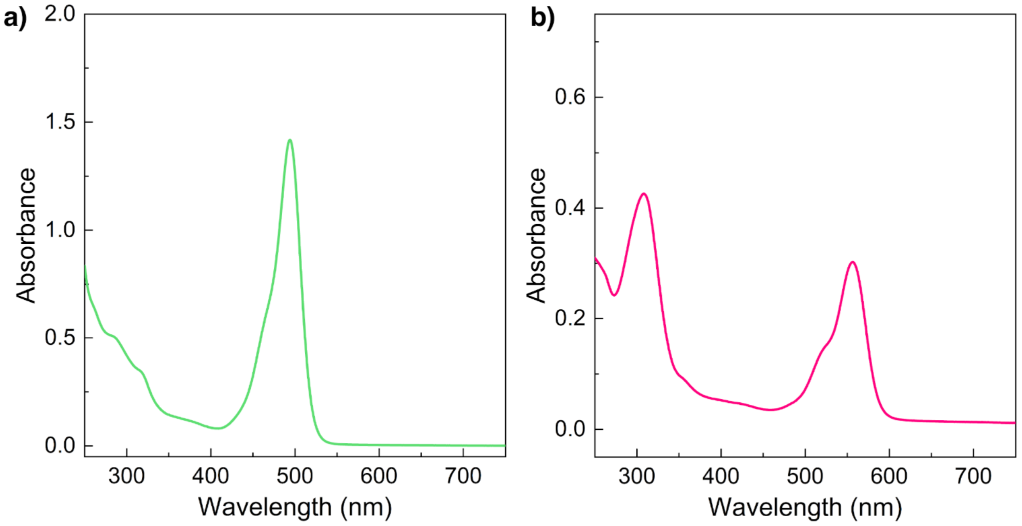

Figure S11. UV-vis absorption spectra of aqueous solutions (1 mg mL^{-1} in PBS 10 mM, pH ≈ 7.4) of copolymer (2) fluorescently labeled with FITC (a) and RITC (b).

S2.4. AuNPs synthesis

Citrate-stabilized gold nanoparticles (AuNPs) were synthezised according to a previously established procedure.[7] An aqueous solution of $HAuCl_4 \cdot 3H_2O$ (104.5 mg, 265 µmol, 0.01% w/v, 1045 mL) was heated to 82 °C upon stirring on a hotplate. To this, an aqueous solution of trisodium citrate (366.0 mg, 1.23 mmol, 1% w/v, 36.6 mL) heated at 60 °C was added. The yellow solution was stirred and after 5 minutes its color changed to pale yellow, grey, dark blue, dark wine and finally it turned red. The mixture was left for 15 minutes further at 82 °C and cooled down to room temperature. After overnight stirring at room temperature, the red dispersion was stored at 4 °C.
The aqueous dispersion of AuNPs (0.05 mg mL^{-1}, 3 mL) was analyzed by UV-vis absorption spectroscopy. The effective concentration of Au^0 for subsequent ligand exchange was calculated based on the absorbance at 400 nm ($\varepsilon_{400\,nm}$ = 2400 M^{-1} cm^{-1}), following a previously established method (c_{Au^o} = 0.25 mM).[8] The maximum absorption peak relative to the surface plasmon resonance (SPR) band was identified at 521 nm. The same dispersion was used for TEM and DLS analyses, obtaining a diameter of the gold core of d_{core} = 12 ± 1 nm and a hydrodynamic diameter of d_H = 12 ± 3 nm, respectively (Supplementary Figure S12).

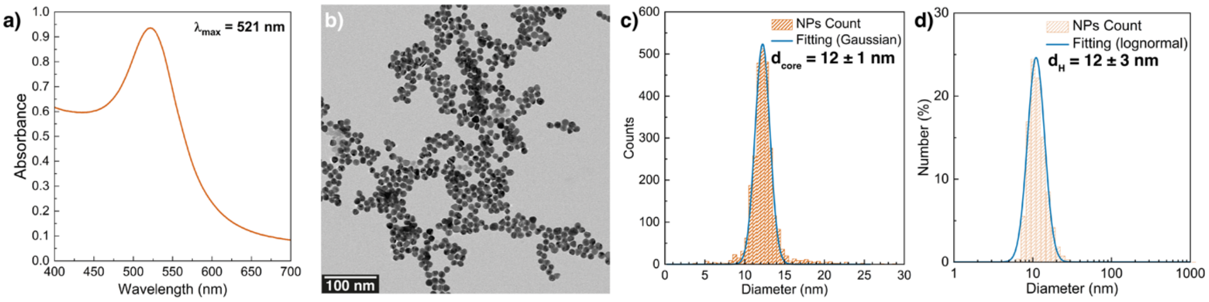

Figure S12. Characterization of citrate-stabilized AuNPs. a) UV-vis absorption spectrum acquired in Milli-Q water at a concentration of 0.05 mg mL^{-1}. b) Representative TEM image of citrate-stabilized AuNPs. c) Plot showing nanoparticle size distribution determined from TEM images similar to that in (b). d) Plot showing nanoparticle size analysis as determined by DLS from a 0.05 mg mL^{-1} dispersion in Milli-Q water.

S2.5. Synthesis of copolymer (3)

Poly(*N*,*N*-dimethylacrylamide-*co*-*N*-acryloxy succinimide-*co*-methacryloxyethyl thiocarbamoyl rhodamine B) (3) was synthesized by free radical polymerization. *N,N*-Dimethylacrylamide (DMAM) and α,α,'-Azoisobutyronitrile (AIBN) were used as purchased without recrystallization. DMAM (911.6 mg, 9.2 mmol, χ = 0.949), *N*-Acryloxy succinimide (AANHS, 82.0 mg, 0.5 mmol, *χ* = 0.05), methacryloxyethol thiocarbamoyl rhodamine B (RhB, 6.5 mg, 9.7 µmol, χ = 0.001) and AIBN (79.6 mg, 0.5 mmol, 5% mol) were dissolved in acetonitrile (3 mL) in a vial equipped with a stirrer bar, adding DMF (300 µL) as internal standard to assess the monomer conversion via ^{1}H-NMR. The vial was sealed, and the polymerization was carried out at 65 °C overnight under stirring. The polymer was isolated by precipitation from hexane/Et_2O (1:1, 80 mL) as a crystalline pink powder (883.4 mg, 88%). M_n = 2,300 g mol^{-1} (^{1}H-NMR), Conversion = 100% (^{1}H-NMR), 5% mol AANHS (^{1}H-NMR).

^{1}H-NMR (Supplementary Figure S13, 400 MHz, $CDCl_3$): δ (ppm) = 3.20-2.80 (6H, -N(C**H$_3$**)$_2$), 2.80-2.77 (0.20H, -(C**H**-C**H$_2$**)-, AANHS), 2.77-2.37 (1.17H, -(C**H**-CH$_2$)-, DMAM + -(C**H**-CH$_2$)-, AANHS), 2.01-1.40 (1.86H, -(CH-C**H$_2$**)-, DMAM + -(CH-C**H$_2$**)-, AANHS), 1.40-1.10 (0.61H, -C(C**H$_3$**)$_2$CN).

^{13}C-NMR (Supplementary Figure S14, 100.5 MHz, $CDCl_3$): δ (ppm) = 174.9 (**C**=O, DMAM), 37.2 + 35.9 (-N(**C**H$_3$)$_2$, DMAM), 36.4 (-[**C**H-CH$_2$]$_n$-), 34.7 (-[CH-**C**H$_2$]$_n$-), 28.9 (-C(**C**H$_3$)$_2$CN), 25.9 (-(**C**H$_2$-**C**H$_2$)-, AANHS).

TD-GPC: *dn/dC* = 0.158 ± 0.002 mL g^{-1}; M_n = 37 ± 9 x 10^3 g mol^{-1}, M_w = 93 ± 17 x 10^3 g mol^{-1}, *Đ* = 2.5 ± 0.2. Values calculated by the average of two separate measurements, error is expressed as the standard deviation.

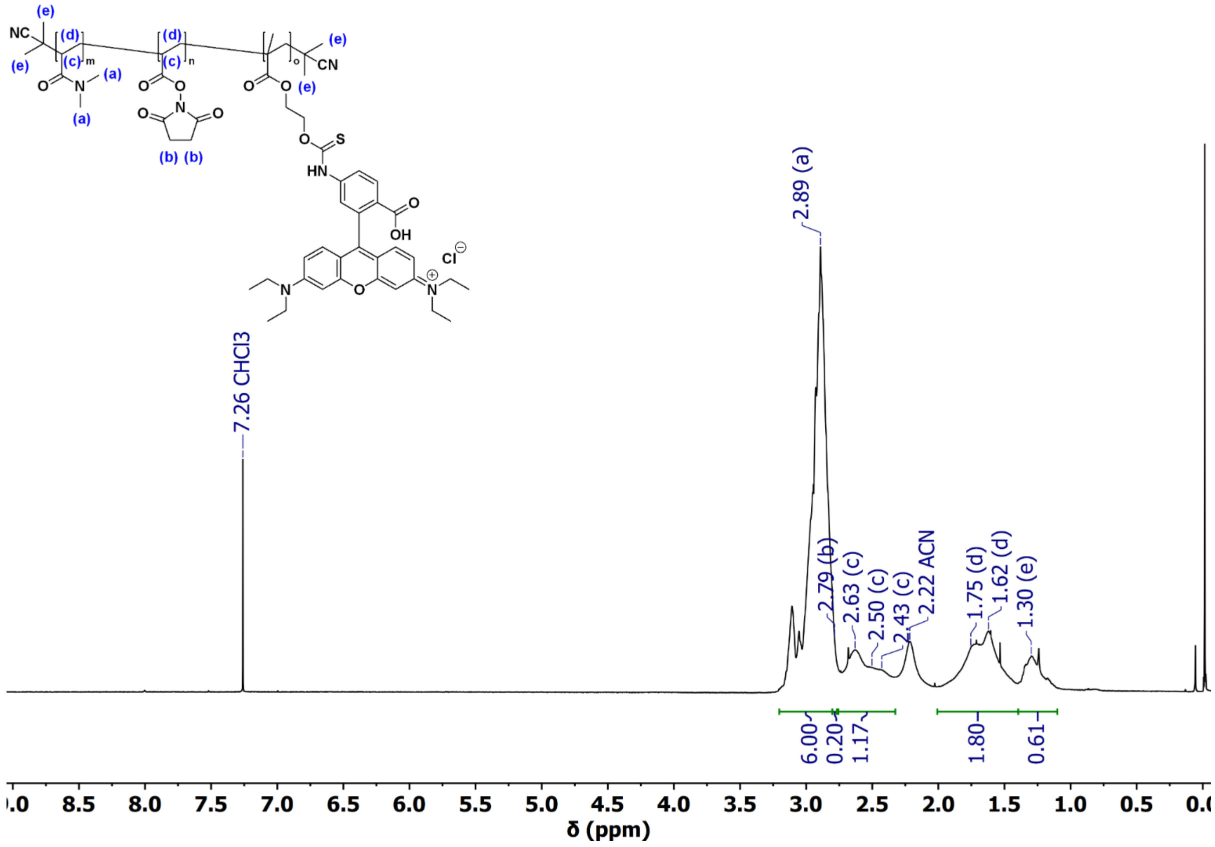

Figure S13. ^{1}H-NMR (400 MHz, $CDCl_3$) spectrum of copolymer (3), recorded in $CDCl_3$ and referenced against the peak of residual $CHCl_3$ at 7.26 ppm.

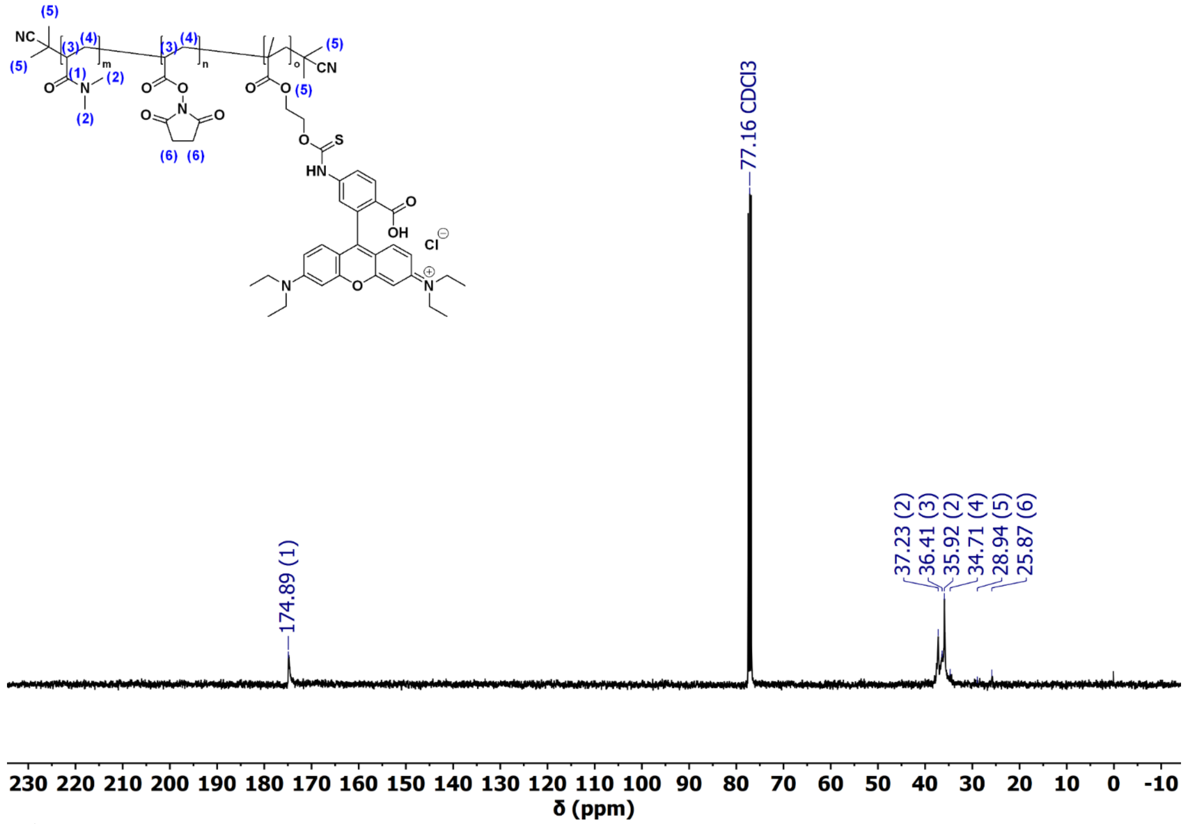

Figure S14. ^{13}C-NMR (100.5 MHz, $CDCl_3$) spectrum of copolymer (3), recorded in $CDCl_3$ and referenced against the peaks of $CDCl_3$ at 77.16 ppm.

S2.6. Synthesis of copolymer (4)

Poly(*N*,*N*-dimethylacrylamide-*co*-aminoethylmethacrylate) (4) was synthesized using a free radical polymerization technique. *N,N*-Dimethylacrylamide (DMAM), 2-aminoethyl methacrylate hydrochloride (AEMA) and α,α,'-Azoisobutyronitrile (AIBN) were used as purchased without recrystallization. DMAM (919.2 mg, 9.3 mmol, χ = 0.95), AEMA (80.8 mg, 0.5 mmol, χ = 0.05), and AIBN (80.1 mg, 0.5 mmol, 5% mol) were dissolved in DMSO (3 mL) in a vial equipped with a stirrer bar, adding DMF (300 µL) as internal standard to assess the monomer conversion via ^{1}H-NMR. The vial was sealed, and the polymerization was carried out at 65 °C overnight under stirring. The polymer was isolated by precipitation from toluene (80 mL) the first time and from Et_2O (160 mL) the second time to obtain the product as a crystalline light-yellow powder (596.1 mg, 60%). M_n = 2,200 g mol^{-1} (^{1}H-NMR), Conversion = 100% (^{1}H-NMR), 5% mol AEMA (^{1}H-NMR).
^{1}H-NMR (Supplementary Figure S15, 400 MHz, $CDCl_3$): δ (ppm) = 4.65-4.06 (0.10H, -O-C**H_2**-, AEMA), 3.45-3.24 (0.10H, -C**H_2**-NH_2, AEMA), 3.19-2.75 (6H, -N(C**H_3**)$_2$, DMAM), 2.74-2.41 (1H, -(C**H**-CH_2)-, DMAM), 2.10-1.87 (0.64H, -C**H_2**-, AEMA), 1.87-1.43 (2H, -(CH-C**H_2**)-, DMAM), 1.42-1.23 (0.46H, -C(C**H_3**)$_2$CN), 1.25 + 1.11-0.73 (0.17H, -C**H_3**, AEMA).
^{13}C-NMR (Supplementary Figure S16, 100.5 MHz, $CDCl_3$): δ (ppm) = 174.9 (**C**=O, DMAM), 37.3 + 35.9 (-N(**C**H_3)$_2$, DMAM), 36.6 (-[**C**H-**C**H_2]$_n$-).
TD-GPC: *dn/dC* = 0.142 ± 0.011 mL g^{-1}; M_n = 340 ± 120 x 10^3 g mol^{-1}, M_w = 920 ± 30 x 10^3 g mol^{-1}, $Đ$ = 2.8 ± 0.9. Values calculated by the average of two separate measurements, error is expressed as the standard deviation.

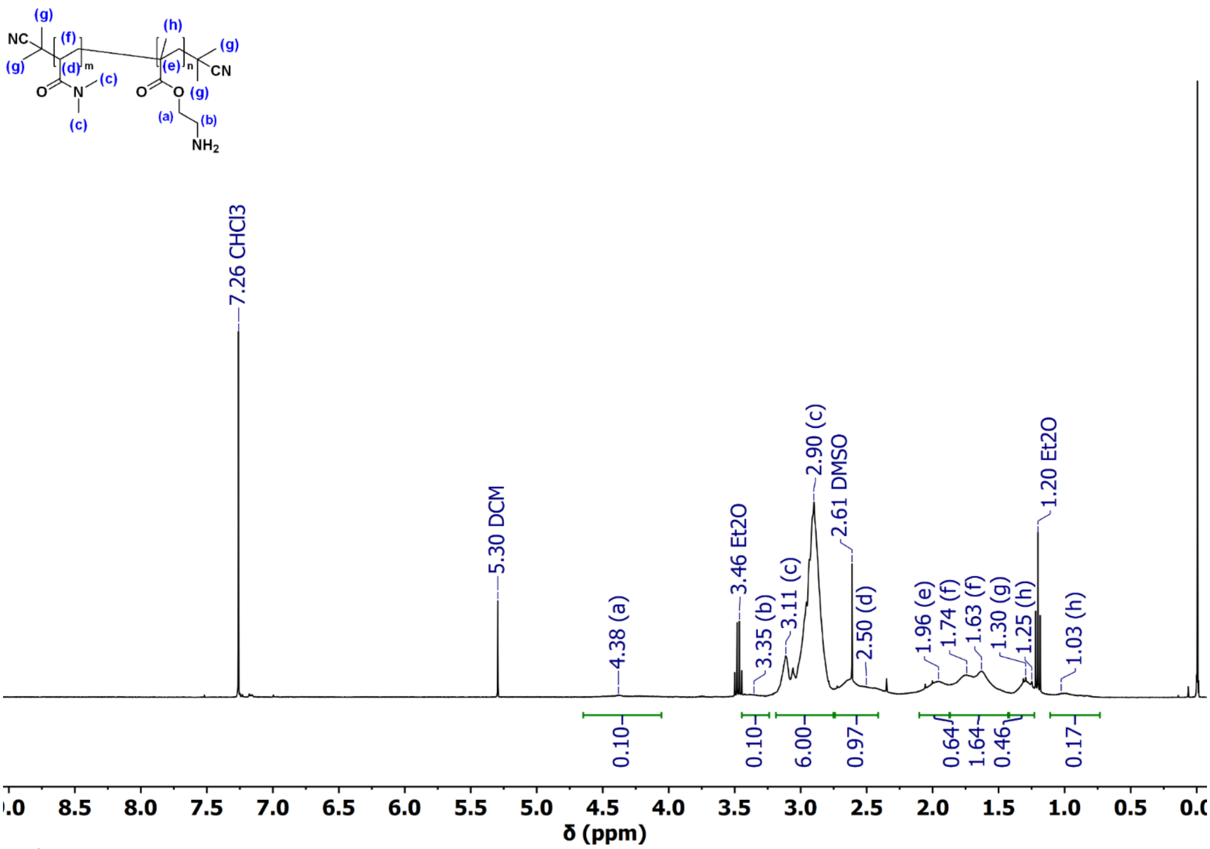

Figure S15. ^{1}H-NMR (400 MHz, $CDCl_3$) spectrum of copolymer (4), recorded in $CDCl_3$ and referenced against the peak of residual $CHCl_3$ at 7.26 ppm.

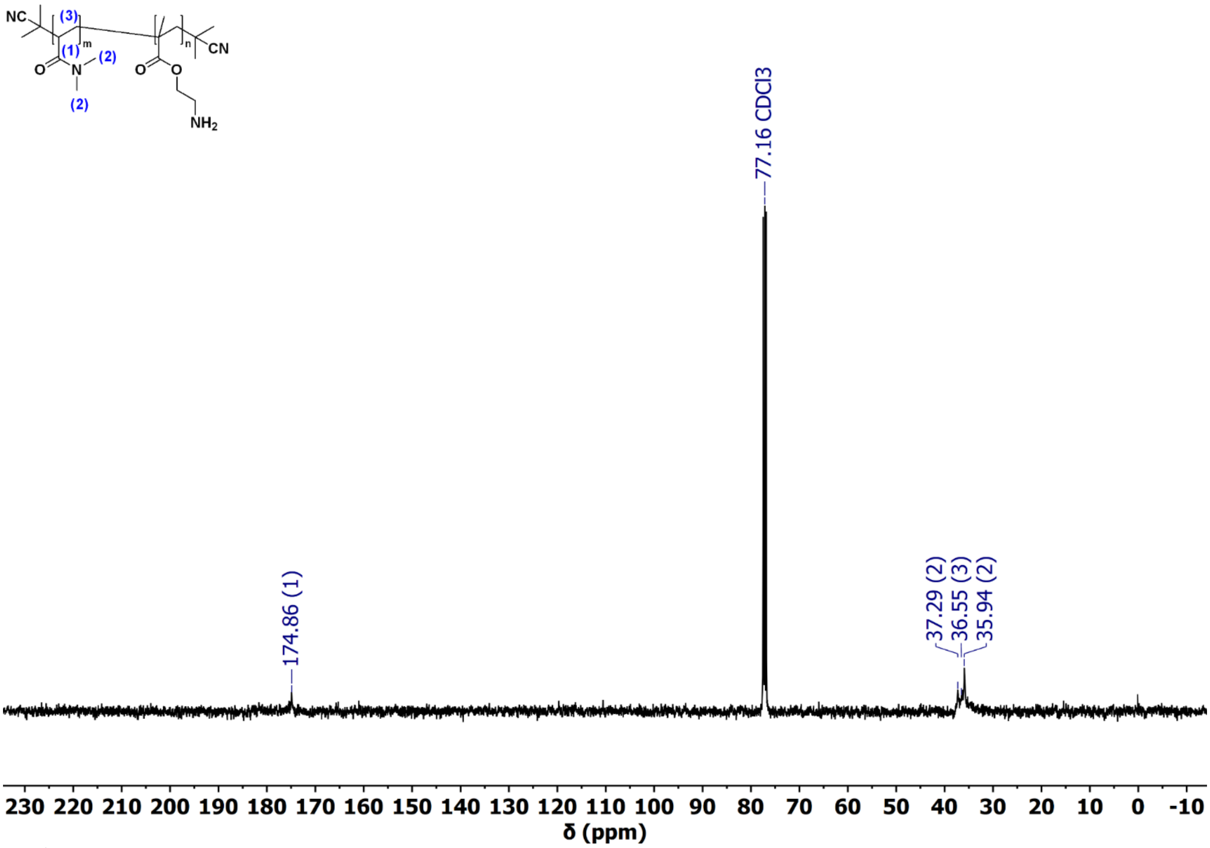

Figure S16. ^{13}C-NMR (100.5 MHz, $CDCl_3$) spectrum of copolymer (4), recorded in $CDCl_3$ and referenced against the peaks of $CDCl_3$ at 77.16 ppm.

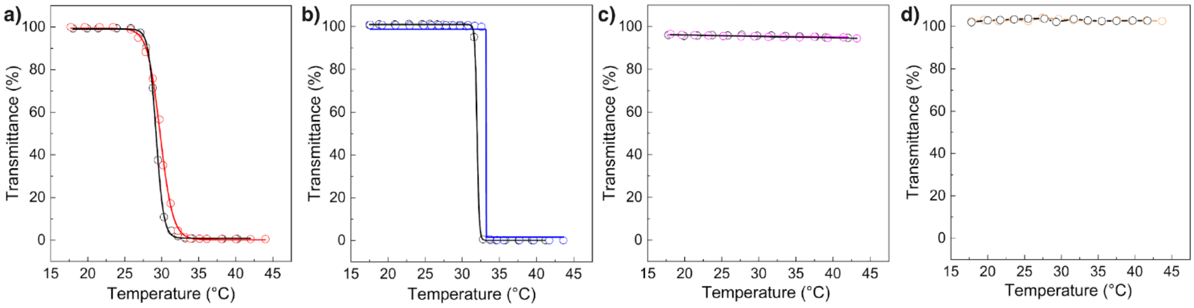

Figure S17. Characterization of polymers (1) – (4) thermoresponsive properties (2 mg mL^{-1} polymer solutions in Milli-Q water). The temperature-dependent changes in transmittance were measured at 550 nm using a UV-vis spectrophotometer. a) Copolymer (1), forward temperature scan (red points) and reverse temperature scan (black points). b) Copolymer (2), forward temperature scan (blue points) and reverse temperature scan (black points). c) Copolymer (3), forward temperature scan (purple points) and reverse temperature scan (black points). d) Copolymer (4), forward temperature scan (yellow points) and reverse temperature scan (black points).

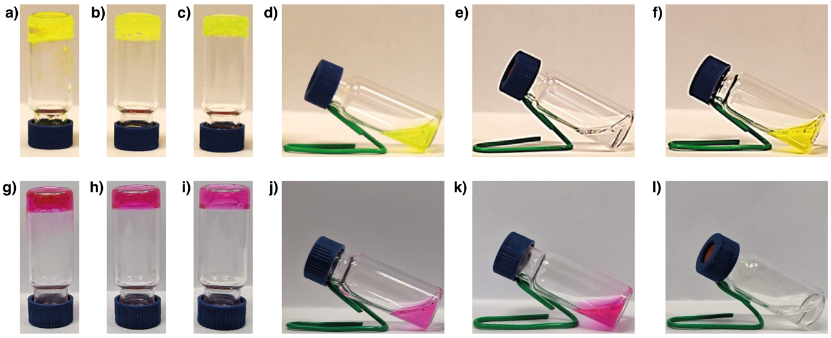

Figure S18. Inverted vial test demonstrating formation of hydrogels by mixing aqueous solutions of polymers (1) and (2), or polymers (3) and (4) at different concentrations in Na_2CO_3 buffer (100 mM, pH ~ 8.5). a) Polymer (1) and FITC-labeled polymer (2) mixed at a final concentration of 90 mg mL^{-1}; b) 60 mg mL^{-1}; c) 30 mg mL^{-1}; d) 15 mg mL^{-1}. e) Negative control sample containing only polymer (1) at a concentration of 30 mg mL^{-1}. f) Negative control sample containing only FITC-labeled (2) at a concentration of 30 mg mL^{-1}. g) Polymer (3) and (4) mixed at final concentration of 60 mg mL^{-1}; h) 30 mg mL^{-1}; i) 15 mg mL^{-1}; j) 7.5 mg mL^{-1}. k) Negative control sample containing only polymer (3) at a concentration of 15 mg mL^{-1}. l) Negative control sample containing only polymer (4) at a concentration of 15 mg mL^{-1}.

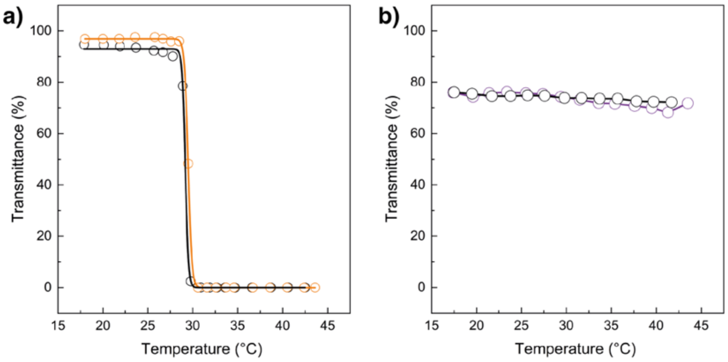

Figure S19. Characterization of the thermoresponsive properties of PNIPAM and PDMAM bulk hydrogels. The temperature-dependent changes in transmittance were measured at 550 nm using a UV-vis spectrophotometer. a) Plot showing the temperature-dependent changes in transmittance for a PNIPAM hydrogel formed by mixing copolymers (1) and (2) at a final concentration of 30 mg mL^{-1} in Na_2CO_3 buffer (100 mM, pH ~ 8.5). b) Plot showing the temperature-dependent changes in transmittance for a PDMAM hydrogel formed by mixing copolymers (3) and (4) at a final concentration of 30 mg mL^{-1} in Na_2CO_3 buffer (100 mM, pH ~ 8.5).

S2.7. PEG$_{2000}$SH synthesis

The thiolated PEG (7) was synthesized in three steps from commercially available poly(ethylene glycol) methyl ether (M_n = 2,000 g mol^{-1}) following the general synthetic scheme highlighted in Supplementary Figure S20.

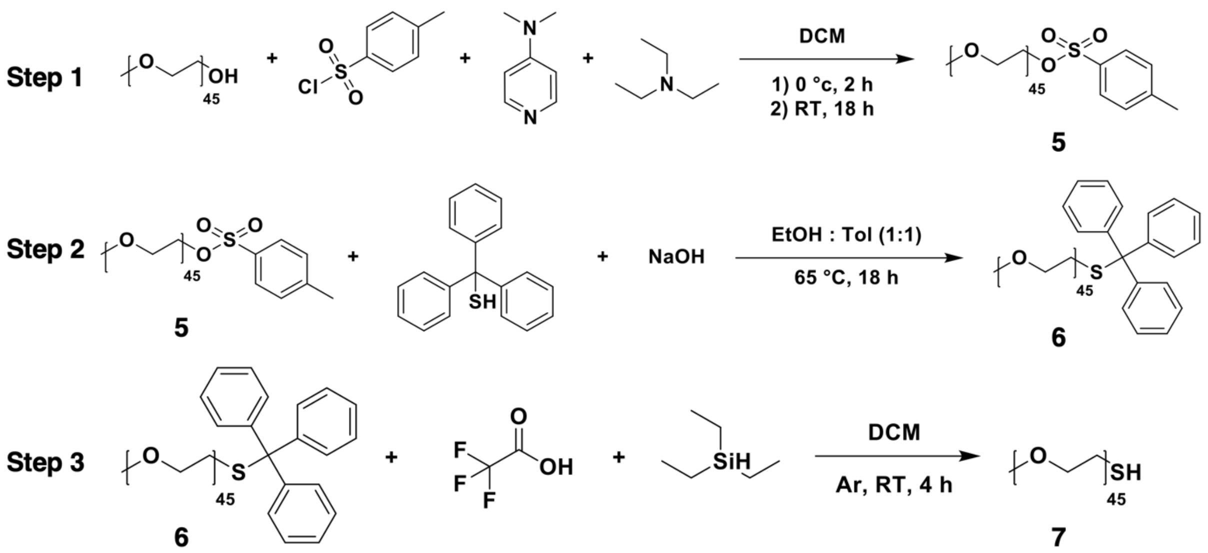

Figure S20. General scheme for the synthesis of thiolated PEG derivative (7).

S2.7.1. Synthesis of tosylated PEG derivative (5)

Compound (5) was synthesized adapting a previously established procedure.[9] Polyethylene glycol monomethyl ether (15 g, 7.45 mmol, 1 eq) was dissolved in DCM (75 mL) in a 250 mL round bottom flask. The solution was cooled down to 0 °C, and 4-dimethylaminopyridine (DMAP, 94.8 mg, 0.78 µmol, 0.1 eq) and triethylamine (TEA, 1.74 g, 17.2 mmol, 2.0 eq) were added. 4-toluenesulfonyl chloride (TsCl, 2.4 g, 12.6 mmol, 1.7 eq) was subsequently added dropwise to the solution and the reaction mixture was stirred at room temperature overnight. The reaction mixture was washed with brine three times and dried over $MgSO_4$. The filtered solution was purified by precipitation in Et_2O at 0°C upon vigorous stirring to obtain the product as a white solid (12.9 g, 80%).
^{1}H-NMR (Supplementary Figure S21, 400 MHz, $CDCl_3$): δ (ppm) = 7.80 (d, J = 8.4 Hz, 2H, o-C**H**, Ts), 7.34 (d, J = 8.5 Hz, 2H, m-C**H**, Ts), 4.15 (t, J = 4.7 Hz, 2H, -C**H**$_2$-OTs), 3.72 – 3.51 (m, 185H, -[O-C**H**$_2$-C**H**$_2$]$_n$-), 3.38 (s, 3H, C**H**$_3$-O-), 2.45 (s, 3H, -C-C**H**$_3$).

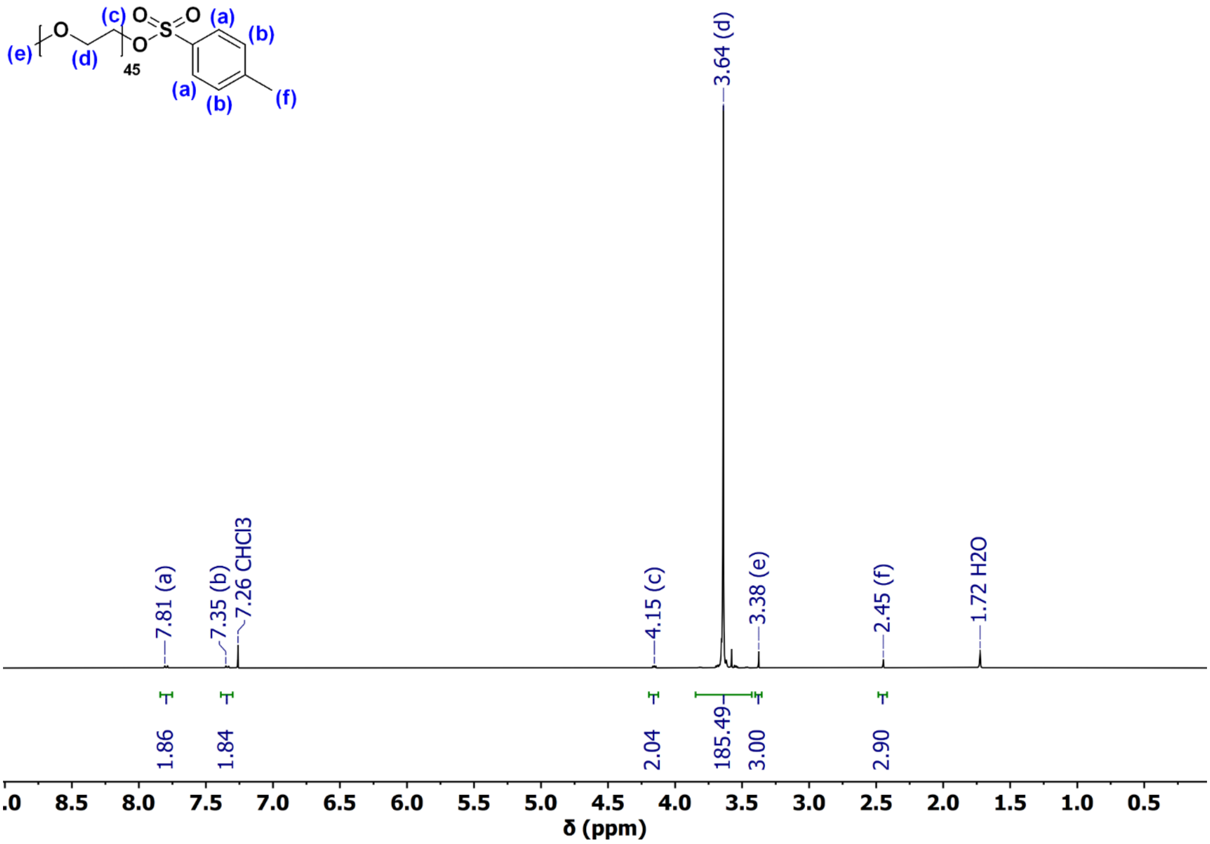

Figure S21. ^{1}H-NMR (400 MHz, $CDCl_3$) spectrum of (5), recorded in $CDCl_3$ and referenced against the peak of residual $CHCl_3$ at 7.26 ppm.

S2.7.2. Synthesis of trityl PEG derivative (6)

Compound (6) was synthesized adapting a previously established procedure.[10] Triphenylmethanethiol (TrtSH, 2.06 g, 7.45 mmol, 2.25 eq) was dissolved in a solution of EtOH/toluene (1:1, 120 mL), to which NaOH (331.3 mg, 8.28 mmol, 2.5 eq) in H_2O (12 mL) was added. To this mixture, a solution of (5) (7.19 g, 3.31 mmol, 1 eq) in EtOH/toluene (1:1, 20 mL) was added and the reaction mixture was stirred at 65 °C overnight. The solvent was removed under reduced pressure and the yellow solid was dissolved in DCM (25 mL) and washed

with saturated $NaHCO_3$ solution once and brine three times. The organic layer was dried over $MgSO_4$, and the filtered solution was concentrated under reduced pressure. The crude product was purified by precipitation in Et_2O at 0°C upon vigorous stirring to obtain the product (6) as a white-beige solid (4.1 g, 54%).

^{1}H-NMR (Supplementary Figure S22, 400 MHz, $CDCl_3$): δ = 7.40 (d, 6H, o-C**H**, Trt), 7.27 (d, 6H, m-C**H**, Trt + 8H $CHCl_3$), 7.20 (t, 3H, p-C**H**, Trt), 3.86 – 3.42 (m, 181H, -[O-C**H₂**-C**H₂**]n-), 3.38 (s, 3H, C**H₃**-O-), 3.30 (t, 2H, -O-C**H₂**-CH₂-S-), 2.42 (t, 2H, -O-CH₂-C**H₂**-S-).

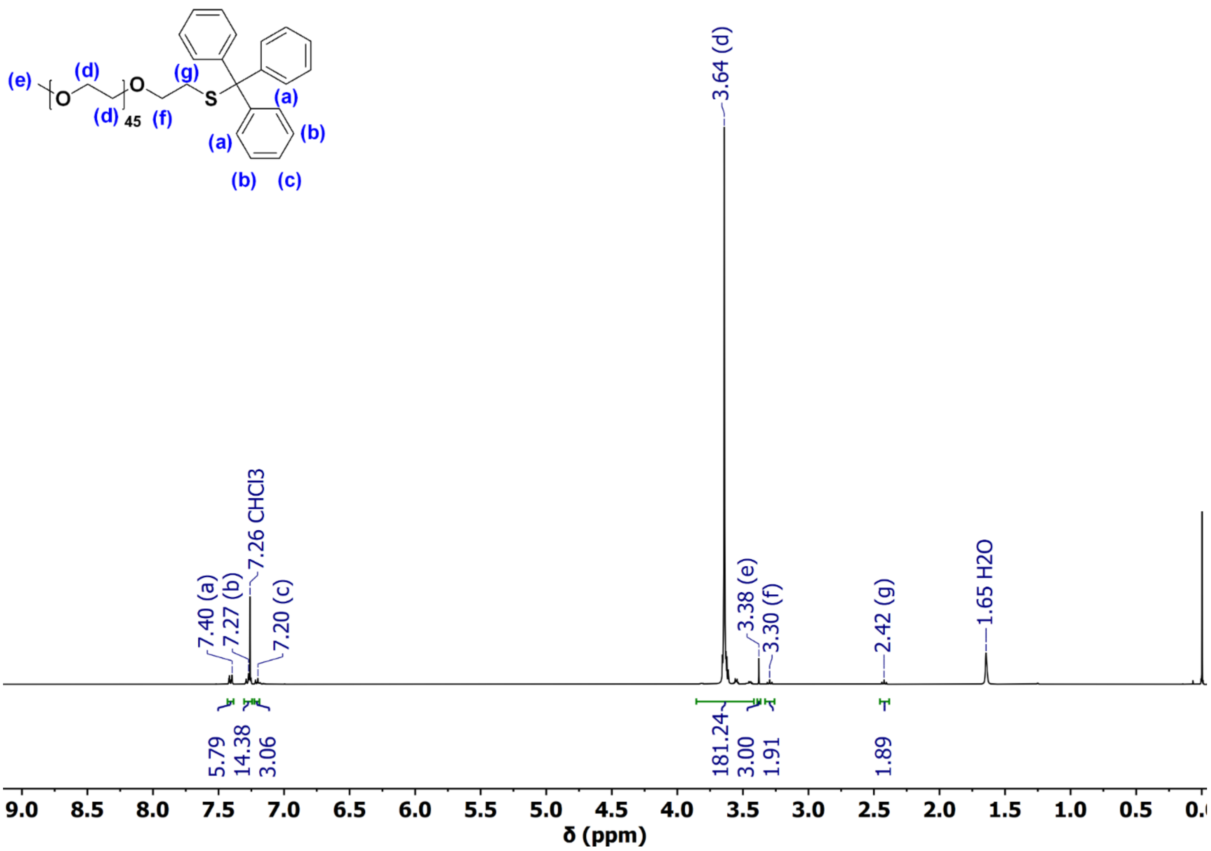

Figure S22. ^{1}H-NMR (400 MHz, $CDCl_3$) spectrum of (6), recorded in $CDCl_3$ and referenced against the peak of residual $CHCl_3$ at 7.26 ppm.

S2.7.3. Synthesis of thiolated PEG derivative (7)

Compound (7) was synthesized adapting a previously established procedure.[10] Compound (6) (1 g, 440 µmol, 1 eq) was dissolved in DCM (35 mL) in a 100 mL round bottom flask and argon was bubbled in it for 10 minutes. Trifluoroacetic acid (TFA, 1.68 mL, 22.0 mmol, 5% v/v) and triethylsilane (TES, 141 µL, 880 µmol, 2 eq) were added and argon was bubbled again for 10 minutes. The reaction mixture was stirred at room temperature for 4 h. Subsequently, the solvent and the TFA were removed under reduced pressure and the yellow solid was dissolved in DCM (10 mL) and washed with saturated $NaHCO_3$ solution once and brine three times. The organic layer was dried over $MgSO_4$, and the filtered solution was concentrated under reduced pressure. The crude product was purified by precipitation in petroleum ether at 0°C upon vigorous stirring to obtain the product as a yellow solid (425.0 mg, 48%).

^{1}H-NMR (Supplementary Figure S23, 400 MHz, $CDCl_3$): δ (ppm) = 3.87 – 3.52 (m, 169H, -[O-C**H₂**-C**H₂**]n-), 3.47 (t, 2H, -O-C**H₂**-CH₂-SH), 3.37 (s, 3H, C**H₃**-O-), 2.70 (m, 2H, -O-CH₂-C**H₂**-SH), 1.59 (t, 1H, -CH₂-S**H**).

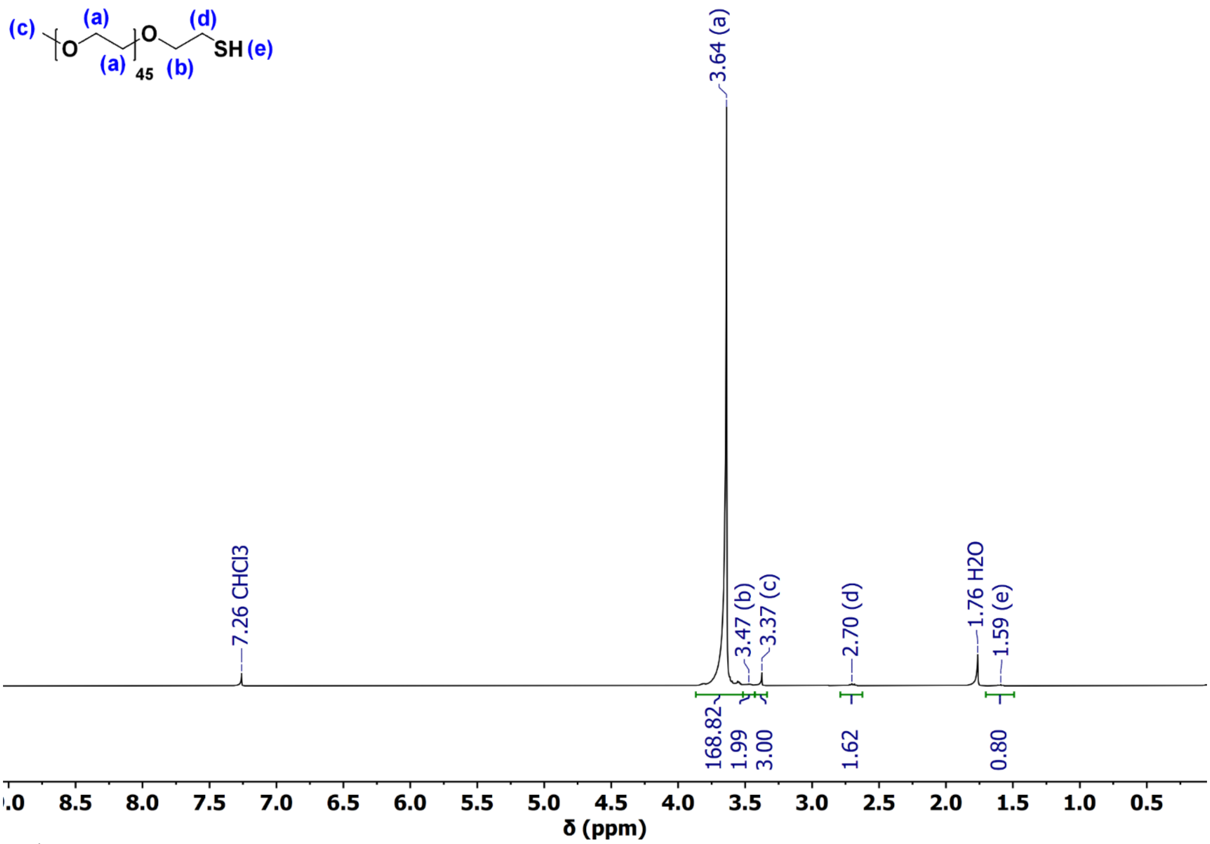

Figure S23. ^{1}H-NMR (400 MHz, $CDCl_3$) spectrum of (7), recorded in $CDCl_3$ and referenced against the peak of residual $CHCl_3$ at 7.26 ppm.

S2.8. PEG-AuNPs ligand exchange

PEG-protected AuNPs were prepared by adding an aqueous solution of compound (7) (243.1 mg, 200 mg mL^{-1}, 1.215 mL) to 150 mg of the aqueous dispersion of citrate-stabilized AuNPs (150 mL). The red dispersion was left overnight without stirring at room temperature. Subsequently, the now darker solution was concentrated *in vacuo* and the excess of compound (7) was removed by centrifugation (4 cycles, 14,600 rpm for 30 minutes). The pellet was redispersed in Milli-Q H_2O, it was centrifuged one last time (14,600 rpm, 1 h) and the resulting pellet was freeze-dried to obtain 9.2 mg of Me-PEG_{2000}SH-stabilized AuNPs (PEG-AuNPs) as a dark red solid. The concentration of PEG-AuNPs aqueous dispersions was calculated in mg mL^{-1} based on the weight of the lyophilized PEG-AuNPs powder.

An aqueous dispersion of PEG-AuNPs (0.05 mg mL^{-1}, 3 mL) was analyzed by UV-vis absorption spectroscopy. The maximum absorption peak relative to the surface plasmon resonance band was identified at 527 nm, which was red-shifted with respect to the SPR maximum of citrate-stabilized AuNPs (Supplementary Figure S12), indicating successful ligand exchange. The same dispersion was used for TEM and for DLS analyses, obtaining a substantially unchanged diameter of the gold core (d_{core} = 14 ± 1 nm) and a hydrodynamic diameter of d_H = 23 ± 7 nm, respectively (Supplementary Figure S24). The increase of d_H indicates successful functionalization of the gold nanomaterial upon formation of a thiolated PEG shell around it.

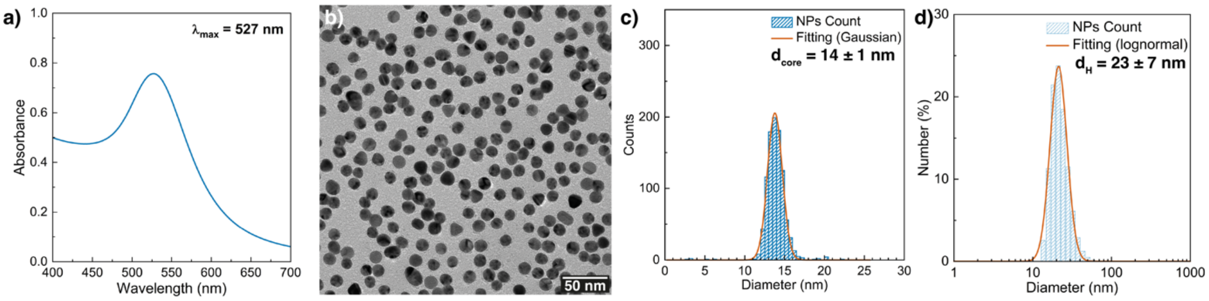

Figure S24. Characterization of PEG thiol-stabilized AuNPs. a) UV-vis absorption spectrum acquired in Milli-Q water at a concentration of 0.05 mg mL^{-1}. b) representative TEM image of PEG thiol-stabilized AuNPs. c) Plot showing nanoparticle size distribution determined from TEM images similar to that in (c). d) Plot showing nanoparticle size analysis as determined by DLS from a 0.05 mg mL^{-1} dispersion in Milli-Q water.

S2.9. AGx labeling with RITC

AGx (20 mg, 2.7 mg mL^{-1}) was dissolved in $Na_2CO_3/NaHCO_3$ (0.1 M, pH ≈ 8.5) buffer. A RITC (1 mg mL^{-1} in DMSO) solution was added dropwise to the stirring enzyme solution. The solution was stirred for 16 h at 4 °C, then purified by dialysis (12-14 kDa MWCO membranes) for 24 h, and lyophilized. The degree of labeling was determined via UV-vis absorption spectroscopy to be equal to: 0.5 (Supplementary Figure S25a).

S2.10. GOx labeling with FITC

GOx (20 mg, 2.7 mg mL^{-1}) was dissolved in $Na_2CO_3/NaHCO_3$ (0.1 M, pH ≈ 8.5) buffer. A FITC (1 mg mL^{-1} in DMSO) solution was added dropwise to the stirring enzyme solution. The solution was stirred for 16 h at 4 °C, then purified by dialysis (12-14 kDa MWCO membranes) for 24 h, and lyophilized. The degree of labeling was determined via UV-vis absorption spectroscopy to be equal to: 2.4 (Supplementary Figure S25b).

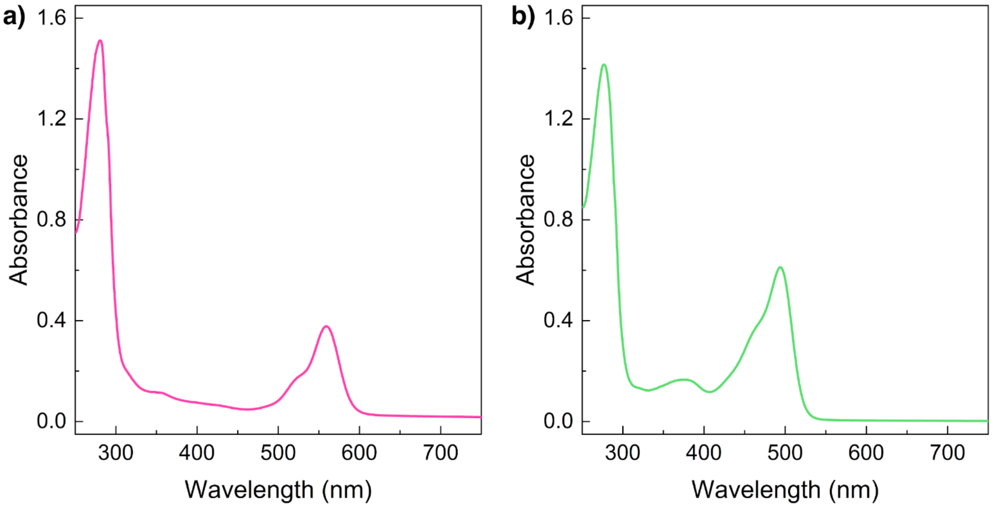

Figure S25. UV-vis absorption spectra of aqueous solutions of RITC-tagged AGx (a) and FITC-tagged GOx (b) in PBS (1 mg mL^{-1}, 10 mM, pH ≈ 7.4).

S3. Proteinosome fabrication:

S3.1. General preparation of PEG-diNHS-crosslinked "empty" proteinosomes

Proteinosome samples were prepared following our published general procedure.[11] In a 1.8 mL vial, 30 µL of an aqueous solution of AMCA- or BDP650-fluorescently labeled azide- or BCN-functionalized BSA/PNIPAM-*co*-MAA nanoconjugates (8 mg mL^{-1}) and 30 µL of PEG-diNHS solution (67 mg mL^{-1}) in Na_2CO_3 buffer (pH 8.5, 100 mM) were mixed together. Subsequently, 1 mL of 2-ethyl-1-hexanol was gently added to the aqueous phase at an aqueous/oil volume fraction (φ_w) of 0.06. The mixture was vigorously shaken manually for 15 s to produce a white turbid dispersion. The Pickering emulsion was readily transferred into an Eppendorf tube, where it was left to crosslink and sediment for at least 16 h.

S3.2. Preparation of proteinosomes enclosing a PNIPAM-based proto-cortex

S3.2.a. Preparation of proteinosomes enclosing a PNIPAM-based proto-cortex

In a 1.8 mL vial, 15 µL of an aqueous solution of AMCA- or BDP650-fluorescently labeled azide- or BCN-functionalized BSA/PNIPAM-*co*-MAA nanoconjugates (16 mg mL^{-1}), 15 µL of non-labeled or FITC-labeled polymer (2) solution (120 mg mL^{-1}) in Na_2CO_3 buffer (pH 8.5, 100 mM), and 15 µL of Milli-Q water were mixed together. Subsequently, 15 µL of polymer (1) solution (120 mg mL^{-1}) in Na_2CO_3 buffer (pH 8.5, 100 mM) were added and mixed thoroughly. Unless otherwise stated, the PNIPAM-based polymers were added in order to obtain a final concentration of each of 30 mg mL^{-1} in the aqueous phase of the emulsion. Finally, 1 mL of 2-ethyl-1-hexanol was gently added to the aqueous phase at an aqueous/oil volume fraction (φ_w) of 0.06. The mixture was vigorously shaken manually for 15 s to produce a white turbid emulsion. The Pickering emulsion was readily transferred into an Eppendorf tube where it was left to crosslink and sediment for at least 16 h.

S3.2.b. Preparation of proteinosomes enclosing a PNIPAM-based proto-cortex and PEG-AuNPs

In a 1.8 ml vial, 15 µL of an aqueous solution of AMCA- or BDP650-fluorescently labeled, or non-labeled azide- or BCN-functionalized BSA/PNIPAM-*co*-MAA nanoconjugates (16 mg mL^{-1}), 15 µL of FITC- or RITC-, or non-fluorescently labeled polymer (2) solution (120 mg mL^{-1}) in Na_2CO_3 buffer (pH 8.5, 100 mM), variable volumes of an aqueous dispersion of PEG-AuNPs (50 mg mL^{-1}) and of Milli-Q water were mixed together to achieve the desired concentration of PEG-AuNPs (see Supplementary Table S1). 15 µL of polymer (1) solution (120 mg mL^{-1}) in Na_2CO_3 buffer (pH 8.5, 100 mM) were then added and mixed thoroughly. Finally, 1 mL of 2-ethyl-1-hexanol was gently added to the aqueous phase at an aqueous/oil volume fraction (φ_w) of 0.06. The mixture was vigorously shaken manually for 15 s to produce a dark red turbid emulsion. The Pickering emulsion was readily transferred into an Eppendorf tube where it was left to crosslink and sediment for at least 16 h.

Table S1. Table summarizing the different volumes and final concentrations (C_{AuNP}) of colloid solutions used to compose the aqueous phase of the Pickering emulsions. The stock aqueous dispersion of PEG-AuNPs had a concentration of 50 mg mL^{-1}. The final volume was 15 µL. V_{AuNP} represents the volume in microliters taken from the stock solution, m_{AuNP} the corresponding mass of PEG-AuNPs. V_{H2O} represents the volume of Milli-Q water used to dilute the volume of PEG-AuNPs stock solution.

Entry	V_{AuNP} (µL)	m_{AuNP} (mg)	V_{H2O} (µL)	C_{AuNP} (mg mL^{-1})
1	0.2	0.01	14.8	0.17
2	0.4	0.02	14.6	0.33
3	0.8	0.04	14.2	0.67
4	1.6	0.08	13.4	1.33
5	3.2	0.16	11.8	2.66
6	6.4	0.32	8.6	5.33
7	12.8	0.64	2.2	10.67

S3.2.c. Preparation of proteinosomes enclosing a PNIPAM-based proto-cortex, PEG-AuNPs, and amyloglucosidase (AGx), or glucose oxidase (GOx)

In a 1.8 mL vial, 15 µL of an aqueous solution of non-labeled azide- or BCN-functionalized BSA/PNIPAM-*co*-MAA nanoconjugates (16 mg mL^{-1}), 15 µL of non-labeled polymer (2) solution (120 mg mL^{-1}) in Na_2CO_3 buffer (pH 8.5, 100 mM), 1.6 µL of an aqueous dispersion of PEG-AuNPs (50 mg mL^{-1}), 10 µL of AGx, or GOx aqueous solution (50 mg mL^{-1}), and 3.4 µL of Milli-Q water were mixed together. 15 µL of polymer (1) solution (120 mg mL^{-1}) in Na_2CO_3 buffer (pH 8.5, 100 mM) were then added and mixed thoroughly. Subsequently, 1 mL of 2-ethyl-1-hexanol was gently added to the aqueous phase at an aqueous/oil volume fraction (φ_w) of 0.06. The mixture was vigorously shaken manually for 15 s to produce a dark red turbid emulsion. The Pickering emulsion was readily transferred into an Eppendorf tube where it was left to crosslink and sediment for at least 16 h.

S3.3. Preparation of proteinosomes enclosing a PDMAM-based proto-cytoskeleton

In a 1.8 ml vial, 15 µL of an aqueous solution of AMCA- or BDP650-fluorescently labeled azide- or BCN-functionalized BSA/PNIPAM-*co*-MAA nanoconjugates (16 mg mL^{-1}), 15 µL of polymer (4) solution (60 mg mL^{-1}) in Na_2CO_3 buffer (pH 8.5, 100 mM), and 15 µL of Milli-Q water were mixed together. 15 µL of polymer (3) solution (60 mg mL^{-1}) in Na_2CO_3 buffer (pH 8.5, 100 mM) were then added and mixed thoroughly. The PDMAM-based polymers were added in order to obtain a final concentration of each of 15 mg mL^{-1} in the aqueous phase of the emulsion. Finally, 1 mL of 2-ethyl-1-hexanol was gently added to the aqueous phase at an aqueous/oil volume fraction (φ_w) of 0.06. The mixture was vigorously shaken manually for 15 s to produce a pink turbid emulsion. The Pickering emulsion was readily transferred into an Eppendorf tube where it was left to crosslink and sediment for at least 16 h.

S4. Programmed assembly of prototissues

S4.1. Assembly of non-patterned and non-layered prototissues

In order to prepare PCMs, we followed the floating mold technique reported in our previous work.[11] Briefly, in a 47 mm Petri dish, 5 mL of an aqueous solution of TWEEN 80 (5 wt.%) was added and a PTFE mold was allowed to float at the air/water interface. Subsequently, equal volumes of azide- and BCN-functionalized sedimented proteinosome emulsions (see Supplementary Table S2) were thoroughly mixed in an Eppendorf tube. A volume of the 1:1 binary proteinosome mixture was then drop-casted in the floating PTFE mold to obtain a specific emulsion volume per unit area (see Supplementary Table S2). The proteinosomes were then left overnight to transfer into the aqueous solution forming a prototissue (also termed protocellular material, PCM). Before use, the prototissue was detached from the mold, transferred into Milli-Q water, where it was left for at least 2 h in order to wash the excess of surfactant.

Table S2. Table summarizing all fluorescent labels and proteinosome compositions used to fabricate all non-patterned and non-stratified prototissues.

	PCM type	Binary emulsion composition: Azide-functionalized proteinosomes	Binary emulsion composition: BCN-functionalized proteinosomes	Emulsion volume per unit area (μL mm^{-2})
**1**	No polymer network	Section S3.1<br>AMCA-nanoconjugate	Section S3.1<br>BDP650-nanoconjugate	0.6
**2**	Contractile PCM	Section S3.2.a<br>AMCA- or BDP 650 nanoconjugate<br>FITC-labeled **2**	Section S3.2.a<br>AMCA- or BDP 650 nanoconjugate<br>FITC-labeled **2**	0.4
**3**	Photonastic PCM	Section S3.2.b<br>AMCA-nanoconjugate<br>RITC-labeled **2**	Section S3.2.b<br>BDP650-nanoconjugate<br>FITC-labeled **2**	0.4
**4**	Enzymatically active photonastic PCM (labeled enzymes)	Section S3.2.c<br>FITC-labeled GOx	Section S3.2.c<br>RITC-labeled AGx	0.6
**5**	Enzymatically active photonastic PCM (non-labeled enzymes)	Section S3.2.c<br>non-labeled GOx	Section S3.2.c<br>non-labeled AGx	0.6
**6**	Non-contractile PCM	Section S3.3<br>AMCA- or BDP 650 nanoconjugate	Section S3.3<br>AMCA- or BDP 650 nanoconjugate	0.4
**7**	Mixed contractile/ non-contractile PCM	Section S3.2.b<br>BDP650-nanoconjugate<br>non-labeled **2**	Section S3.2.a<br>AMCA-nanoconjugate	0.4
**8**	Mixed contractile/ non-contractile PCM	Section S3.2.b, BDP650-nanoconjugate, FITC-labeled **2**<br>+<br>Section S3.3, AMCA-nanoconjugate		0.4

S4.2. Programmed assembly of photonastic prototissues

Equal volumes of BDP650-labeled azide- and BCN-functionalized proteinosome emulsions containing both a PNIPAM-based polymer network and PEG-AuNPs (see Supplementary Section S3.2.b) were mixed thoroughly in an Eppendorf tube. In another Eppendorf tube, equal volumes of AMCA-labeled azide- and BCN-functionalized proteinosome emulsions containing a PDMAM-based polymer network (see Supplementary Section S3.3) were mixed.

In order to make a stratified PCM having a non-contractile and a photo-contractile layer, in a 47 mm Petri dish, 5 mL of Milli-Q water were added and a PTFE mold with a “six-armed starfish” shape (see Supplementary Figure S1d) was allowed to float at the air/water interface. Subsequently, 23.1 µL of the 1:1 PDMAM-containing binary proteinosome emulsion was drop-casted in the mold and left to transfer for 24 h. Subsequently, 23.1 µL of the 1:1 binary proteinosome emulsion containing PNIPAM-based polymer network and PEG-AuNPs was casted on top of the first PCM layer. The six-armed starfish-shaped PCM was left for 48 h in water, then 1 mL of an aqueous solution of TWEEN 80 (5 wt. %) was added into the Petri dish, left for 5 h, and the PCM was transferred to Milli-Q water, where it was left for 24 h before use.

S5. Characterization of prototissue mechanical properties

S5.1. Young's modulus characterization discussion

Indentation experiments (Supplementary Section S1.7.1) performed at 25 °C on the prototissues showed a higher rigidity of the samples containing protocells endowed with polymeric PNIPAM proto-cortex or PDMAM proto-cytoskeleton compared to prototissues formed from protocells without polymer network (Empty), see Supplementary Figure S46. Specifically, at 25 °C prototissues made from protocells endowed with a PDMAM-based proto-cytoskeleton displayed the highest stiffness with a Young's modulus of 1.3 ± 0.5 kPa, followed by prototissues made from protocells endowed with PNIPAM-based proto-cortex, and prototissues made from "empty" protocells, which were characterized by Young's moduli of 0.4 ± 0.2 kPa and 0.17 ± 0.04 kPa, respectively.

When mechanical measurements were carried out at 40 °C, a slight softening of the prototissues made from protocells endowed with a PDMAM-based proto-cytoskeleton (Young's modulus = 0.7 ± 0.1 kPa), and a strong stiffening of the prototissues made from protocells endowed with a PNIPAM-based proto-cortex (Young's modulus = 1.4 ± 0.2 kPa) were observed. Prototissues made from empty protocells displayed a negligible increase in stiffness instead (from 0.17 ± 0.04 kPa at 25 °C to 0.21 ± 0.02 kPa at 40 °C).

While there is a general lack in the literature of works reporting on temperature-dependent changes of the mechanical properties of PDMAM-based hydrogels, the stiffening of PNIPAM-based hydrogel with the increasing temperature is well reported, and our data align well with the literature.[12, 13]

S5.2. Discussion of force-relaxation tests on prototissues

Viscoelastic characterization of prototissues was carried out by force relaxation testing at 25 and 40 °C (Supplementary Section S1.7.2, Supplementary Figures S42, S47 and S48). From these experiments we could calculate instantaneous (E_0) and equilibrium (E_∞) moduli, which give indication on the instantaneous material response and on the material response after complete viscous relaxation, respectively. At 25 °C, instantaneous and equilibrium moduli for a prototissue composed of protocells endowed with a PDMAM proto-cytoskeleton were 2.7 ± 0.3 kPa and 2.4 ± 0.3 kPa, respectively. For a prototissue composed of protocells endowed with a PNIPAM proto-cortex, instantaneous and equilibrium moduli were 1.5 ± 0.5 kPa and 0.9 ± 0.3 kPa, respectively. For a prototissue composed of protocells without a proto-cortex or proto-cytoskeleton instead, instantaneous and equilibrium moduli were 1.0 ± 0.1 kPa and 0.70 ± 0.05 kPa, respectively. These data show that the polymer networks effectively reinforce the structure of the protocell units and consequently provides more robust protocellular materials. This was in line with the results obtained for the Young's modulus, see Supplementary Section S5.1.

At 40 °C, instantaneous and equilibrium moduli for a prototissue composed of protocells endowed with a PDMAM proto-cytoskeleton were 2.1 ± 0.3 kPa and 1.7 ± 0.2 kPa, respectively. For a prototissue composed of protocells endowed with a PNIPAM proto-cortex, instantaneous and equilibrium moduli were 4.5 ± 0.3 kPa and 2.8 ± 1.0 kPa, respectively. For a prototissue composed of protocells without a proto-cortex or proto-cytoskeleton instead, instantaneous and equilibrium moduli were 1.2 ± 0.1 kPa and 1.0 ± 0.1 kPa, respectively. The temperature-dependent changes in the moduli confirm that PDMAM-containing prototissues became slightly softer at higher temperature, whereas PNIPAM-containing prototissues became much stiffer. For those prototissues composed of protocells without a polymer network, the variation of instantaneous and equilibrium moduli with temperature was negligible. This reflects the same temperature-dependent variations observed for the Young's modulus, see Supplementary Section S5.1.

Furthermore, by applying the Fourier transform to $E(t)$ (see Supplementary Section S1.7.2) we could calculate the complex modulus. This provides values of storage ($E'(\omega)$) and loss ($E''(\omega)$) moduli, which characterize the elastic and viscous component of the mechanical response of the material, respectively (Supplementary Figures S42 and S48). For all the prototissue types, the storage ($E'(\omega)$) and loss modulus ($E''(\omega)$) showed only a slight dependence on the frequency in the 0.01-2 Hz range. At 2 Hz the signals were

almost stable, showing no further changes for $E'(\omega)$ and a very slow tendency to approach zero for $E''(\omega)$. Moreover, $E'(\omega)$ was larger than $E''(\omega)$, meaning that such materials can be considered as gels.[14]

At 25 °C, the prototissue containing protocells endowed with the PDMAM proto-cytoskeleton exhibited the highest storage modulus (2.6 ± 0.4 kPa) at 2 Hz, whereas *E'* for the prototissue containing protocells endowed with the PNIPAM proto-cortex was 1.5 ± 0.5 kPa and for prototissue made from empty protocells it was 1.0 ± 0.1 kPa. Also in this case, this trend matches the values of Young's modulus reported above (Supplementary Section S5.1 and Supplementary Figure S46), showing a higher elastic character for prototissues containing protocells endowed with the polymeric network compared to the prototissue made from empty protocells.

Moreover, the loss modulus for the prototissue containing protocells endowed with the PDMAM proto-cytoskeleton was 0.018 ± 0.005 kPa. *E''* for the prototissue containing both protocells endowed with the PNIPAM proto-cortex or empty protocells was 0.03 ± 0.02 kPa. These results indicate that prototissues containing protocells endowed with the PDMAM proto-cytoskeleton exhibit a lower dissipative behavior compared to the other two prototissue types.

Also, at 40 °C $E'(\omega)$ displays a larger value than $E''(\omega)$ for all the prototissue types, indicating that they still behave like gels. Nevertheless, noticeable mechanical property changes were appreciated compared to 25 °C. At 2 Hz the storage modulus of the prototissue containing protocells endowed with the PDMAM proto-cytoskeleton was 2.1 ± 0.4 kPa, whereas *E'* for the prototissue containing protocells endowed with the PNIPAM proto-cortex was 4.8 ± 1.1 kPa and for the prototissue containing empty protocells was 1.1 ± 0.1 kPa. The loss modulus of the prototissue containing protocells endowed with the PDMAM proto-cytoskeleton was 0.016 ± 0.004 kPa, while *E''* for the prototissue containing protocells endowed with the PNIPAM proto-cortex was 0.15 ± 0.07 kPa, and for the prototissue containing empty protocells was 0.008 ± 0.001 kPa. These results show that at 40 °C the prototissue containing protocells endowed with the PDMAM proto-cytoskeleton experienced a slight reduction in the elastic character, while the viscous behavior remained almost unchanged. Prototissue containing protocells endowed with the PNIPAM proto-cortex exhibited instead a consistent increment in the elastic character and a slight increment in the viscous one. Finally, for the prototissue containing empty protocells a slight increment in the elastic character and a decrease in the viscous character were observed.

S6. Numerical analyses on photonastic prototissue

In order to investigate the mechanical interaction between a photo-contractile layer of PNIPAM-based protocells and a non-contractile layer of PDMAM-based protocells within the photonastic prototissue in the shape of a six-armed starfish, we simulated its deformation under light irradiation using FEM analysis (ABAQUS Standard, SIMULIA™, Dassault Systèmes®). The geometry of the structure was reconstructed from photographic images of the six-armed starfish prototissue and is shown in Supplementary Figure S26. The thickness of the PNIPAM- and PDMAM-based protocell layers corresponded to those determined by confocal fluorescence microscopy (see Supplementary Figure S49). In order to simplify the geometry, we assumed that the layers had constant values of 176 mm and 480 mm, respectively. The geometry of the six-armed starfish prototissue was meshed with 105,827 tetrahedral linear elements. In the simulation, the layers were considered in structural continuity by defining suitable contact conditions (tie contact) between the adjacent faces of the two layers. In order to account for the boundary conditions adopted in the experimental test (Supplementary Section S1.6), a cylindrical central region of the structure was considered fixed.

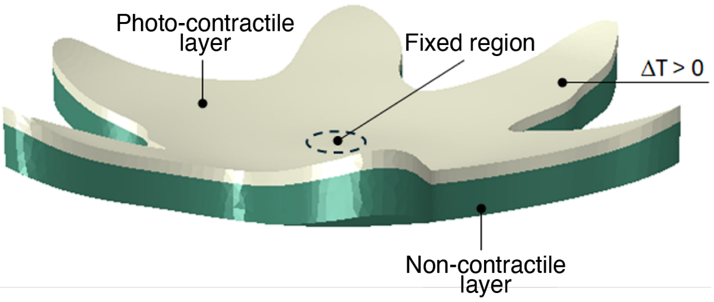

Figure S26. 3D drawing highlighting the geometry of the FEM model of the six-armed starfish prototissue with indication of the boundary and thermal loading conditions.

The contraction of the photo-contractile layer was simulated by applying a temperature variation (*ΔT*) of + 15 °C in a time interval of 50 s, corresponding to the difference between the final (40 °C) and initial (25 °C) temperature of the light irradiation cycles of the experiment, followed by a negative temperature variation resulting from thermal equilibration in a time interval of 50 s upon light switch off. The temperature *vs.* time profile was defined using tabular values according to the experimental data (Figure 2c). We assigned a negative thermal expansion coefficient to the photo-contractile layer with a non-linear profile according to experimental data (Figure 2c), while a null thermal expansion coefficient was assumed for the non-contractile layer.

We adopted an isotropic linear viscoelastic constitutive model for both the photo-contractile and the non-contractile layer. The stiffness of the materials at equilibrium, that is reached at the total development of the viscous phenomena, is defined by the equilibrium longitudinal modulus E_∞. An almost-incompressible behavior was assumed for both the layers, setting a Poisson's ratio $\nu = 0.49$. A Prony series with three pairs of terms was assumed to describe the time-dependent behavior.

We assumed a linear relationship for the equilibrium modulus E_∞ of both the photo-contractile and the non-contractile layers *vs.* temperature starting from their experimental values at 25 °C and 40 °C. In order to account for the variance shown by the indentation experiments, we deduced the upper and lower bounds of the confidence interval at 95% (Supplementary Figure S27). Since the effect as actuator of the photo-contractile layer on the non-contractile layer depends on the ratio of the stiffness of the two layers, we considered three combinations of values of their equilibrium modulus E_∞: *i)* combination of the mean values of E_∞ of photo-contractile and non-contractile layers (results shown in the Main Text); *ii)* combination of the upper bound values of the photo-contractile layer and the lower bound values of E_∞ of the non-contractile layer, indicated in the following as *maxDeflection*; *iii)* combination of the lower bound values of E_∞ of the photo-contractile layer and the upper bound values of E_∞ of the non-contractile layer, indicated in the following as *minDeflection*. In the *maxDeflection* condition, the maximum bending of the non-contractile

layer was obtained, while the opposite occurs in the condition *minDeflection*. This simplified approach to account for the variance of the mechanical properties of the materials could be improved by adopting a Monte Carlo simulation, which, however, would be much more computationally expensive.

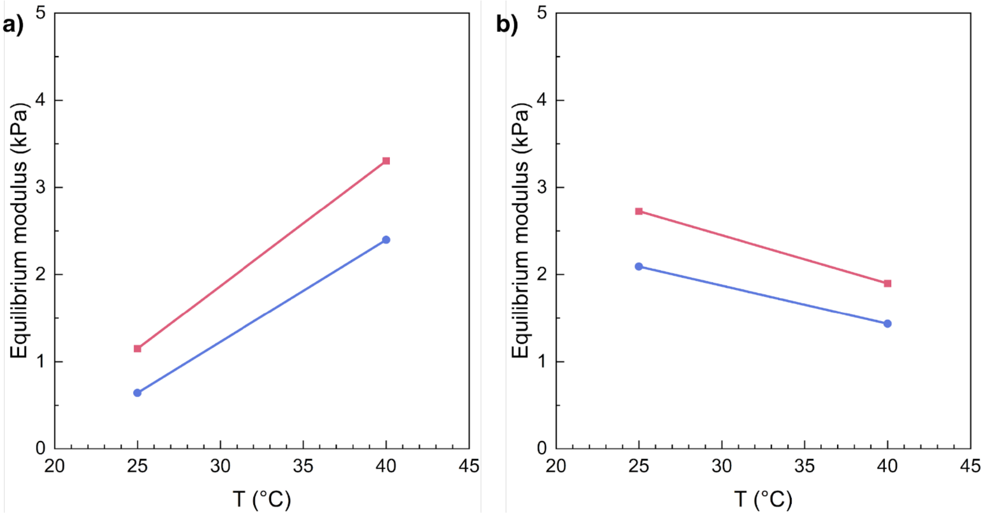

Figure S27. Plots showing the upper (red line) and lower (blue line) bounds for the equilibrium modulus E_∞ of: a) photo-contractile and b) non-contractile layers. E_∞ were determined experimentally at 25 °C and 40 °C (Supplementary Section S5.2), upper and lower values were obtained by adding or subtracting the standard deviation, respectively. Bounds were determined by assuming a linear relationship of E_∞ with temperature.

Non-linear quasi-static analyses were carried out to simulate the deformation path of the structure under a light irradiation half-cycle followed by a light off half-cycle. In order to evaluate the curvature of the structure during the contraction-relaxation cycle, we selected three generic points at each intersection between the external surface of the non-contractile layer and six radial planes (Supplementary Figure S28). For each plane, we calculated the curvature $1/R$, where R is the radius of the circumference defined by the three points; we finally obtained the mean and standard deviation of the curvature.

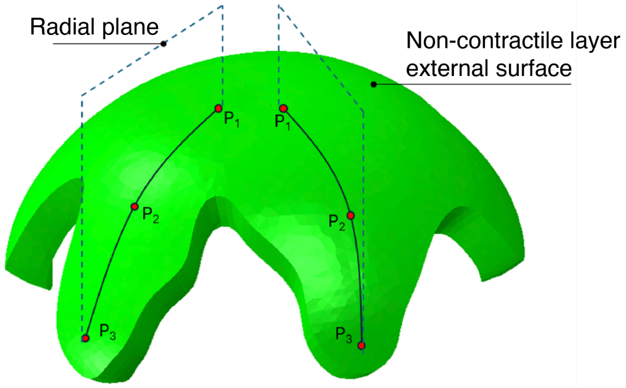

Figure S28. 3D model highlighting the method for the evaluation of the curvature, shown on the deformed configuration of the six-armed starfish prototissue structure in a time instant of the contraction cycle.

Supplementary Figure S29 shows the magnitude displacement fields in the deformed configurations of the photonastic six-armed starfish prototissue for the *maxDeflection* and *minDeflection* conditions, at time t = 50 s (corresponding to the maximum contraction of the photo-contractile layer). In the *maxDeflection* condition, a larger bending of the prototissue was obtained in comparison with the *minDeflection* condition, as expected. Supplementary Figure S30 shows the curvature of the photonastic starfish prototissue *vs.* time for the *maxDeflection* and *minDeflection* conditions, together with the experimental data. Finally, Supplementary Figure S31 shows the stored strain energy and the dissipated viscous energy estimated in the photonastic prototissue during a contraction-relaxation cycle. The stored strain energy and the dissipated viscous energy refer to the whole structure (photo-contractile layer + non-contractile layer).

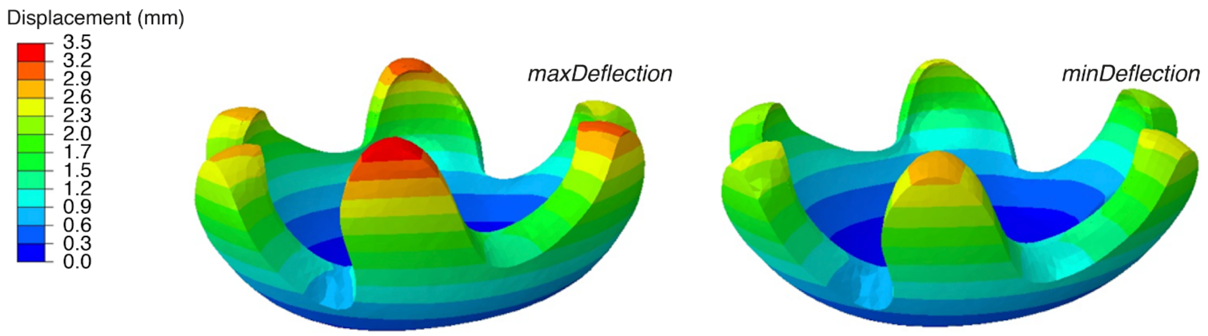

Figure S29. 3D models showing the magnitude displacement of the photonastic starfish prototissue at the maximum contraction under light irradiation (time t = 50 s) of the photo-contractile layer for the *maxDeflection* and *minDeflection* conditions.

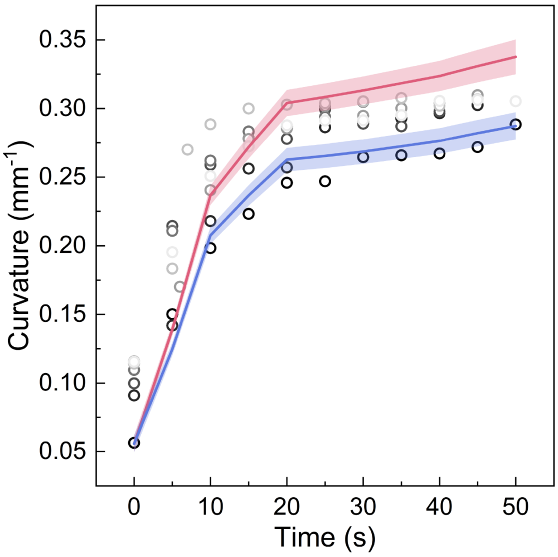

Figure S30. Plot comparing the experimental values of curvature *vs.* time of the photonastic prototissue under light irradiation (grey points – from darker to lighter color corresponding to 1st to 7th cycle) with the curvature simulated by the FEM model for the *maxDeflection* (red line) and *minDeflection* (purple line) conditions. The curvature from the numerical model was calculated along six radial directions and reported as mean ± standard deviation (light colored bands).

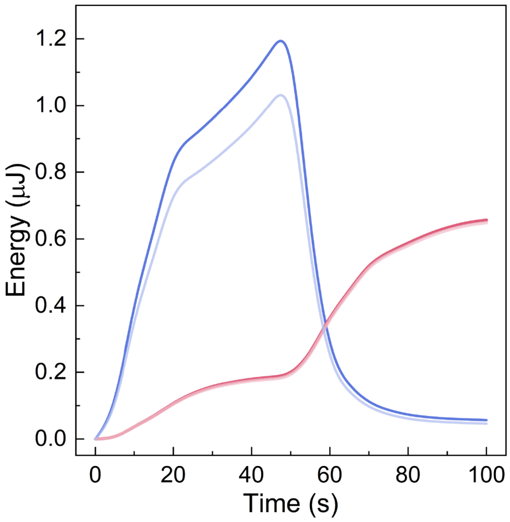

Figure S31. Plot showing stored strain energy (blue lines) and dissipated viscous energy (red lines) *vs.* time evaluated in the FEM model for a light irradiation (0 – 50 s) – light off (51 – 100 s) cycle for the *maxDeflection* (darker colors) and *minDeflection* (lighter colors) conditions. The curves of the dissipated viscous energy for the *maxDeflection* and *minDeflection* conditions are almost coincident.

S7. Supplementary figures

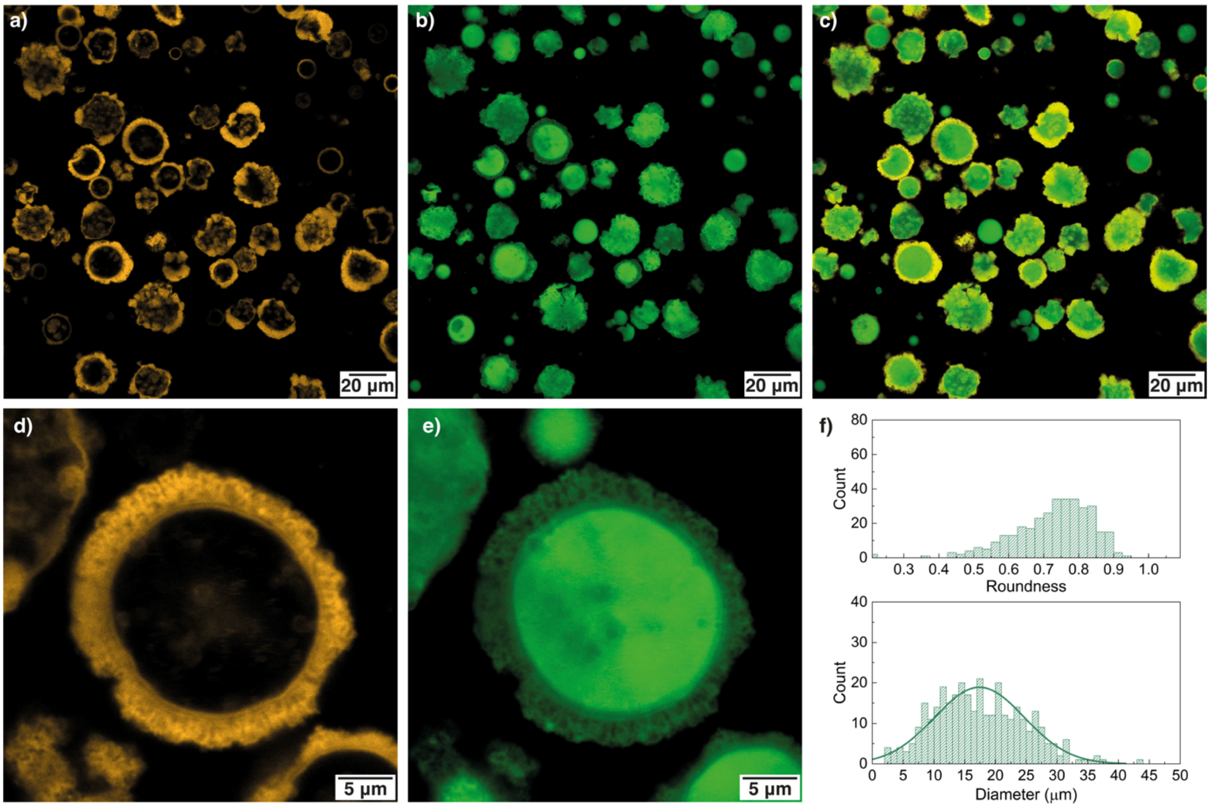

Figure S32. Confocal fluorescence micrograph showing a population of bio-orthogonally reactive proteinosomes in oil containing a polymer network composed of copolymers (1) and (2) (composition: Supplementary Section S3.2.a). a) Fluorescence channel corresponding to the BDP650-labeled bio-orthogonal BSA/PNIPAM-*co*-MAA nanoconjugates (orange fluorescence). b) Fluorescence channel corresponding to the polymer network formed by (1) and FITC-labeled (2) (green fluorescence). c) Merged image of (a) and (b). d-e) Magnification of (a) and (b), respectively. f) Distribution plots showing a mean roundness of 0.75 ± 0.15 equivalent diameter of 17 ± 7 μm, the green solid line represents gaussian fitting of distribution data.

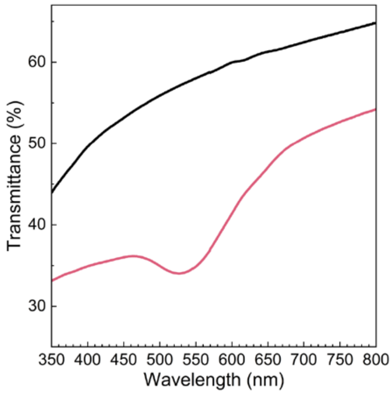

Figure S33. UV-vis transmittance spectra of a prototissue composed of protocells endowed with a PNIPAM proto-cortex and encapsulating PEG-AuNPs (red plot, see composition in Supplementary Section S3.2.b and Table S2, Entry 3), and of a prototissue endowed with a PNIPAM proto-cortex, but not containing AuNPs (black plot, see composition in Supplementary Section S3.2.a and Table S2, Entry 2). Both PCMs were adhered on a glass coverslip and immersed in Milli-Q water.

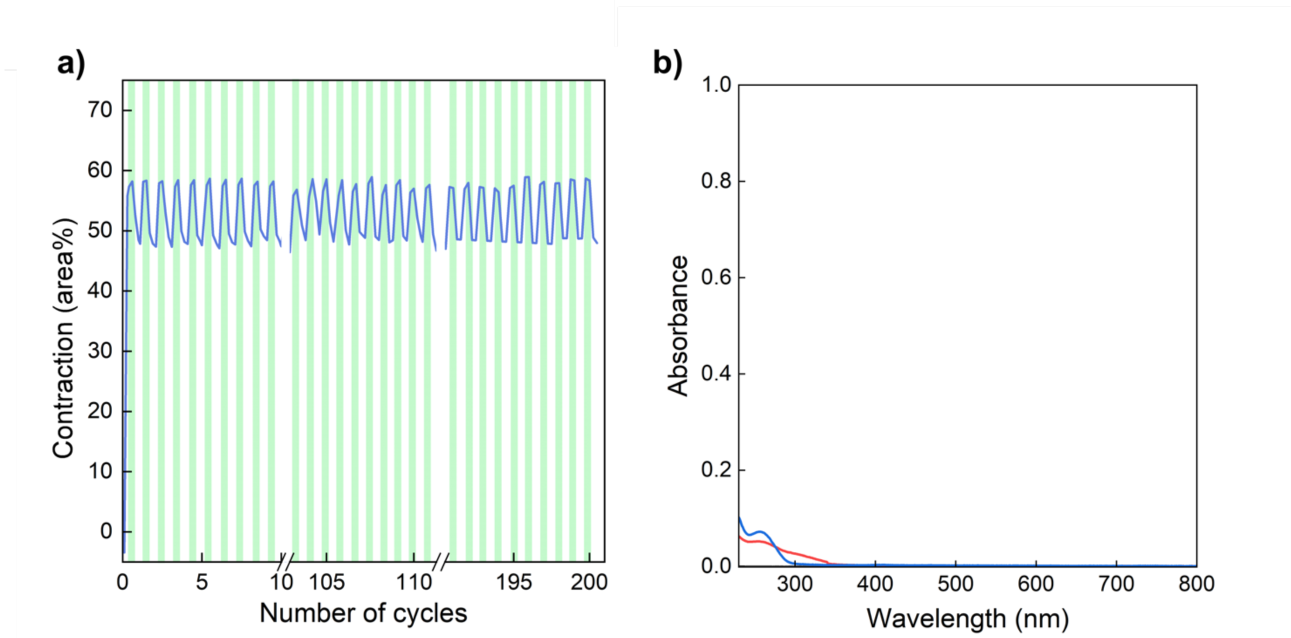

Figure S34. a) Plot showing the light-induced reversible contractions over 200 light-dark cycles (10s light on and 10s off, green areas: light on, *Irr* = 1.35 W cm^{-2}) for a photonastic prototissue 5 mm diameter. b) UV-vis absorption spectra of the aqueous solution in which the prototissue was placed for light-induced reversible contraction experiment. Comparison of the spectra acquired before the experiment (grey plot, basically baseline), after 10 full 0-57% contractions (red plot), and after 200 contraction/relaxation cycles between 47-57 area% (blue plot) showed no evidence of AuNPs leakage (would show presence of the plasmon resonance band at 527 nm) and minimal to no loss of polymeric material.

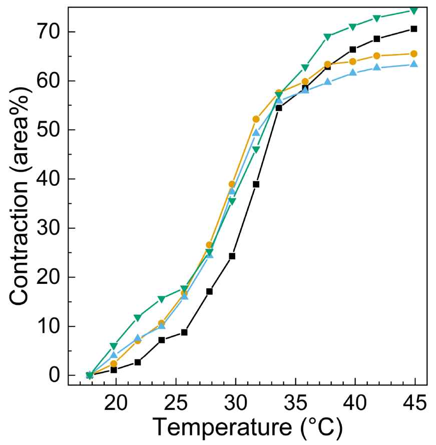

Figure S35. Graph showing the temperature-dependent contraction of a prototissue composed of protocells containing a PNIPAM proto-cortex of different composition. The composition of the proto-cortex was systematically changed by varying the concentration of the stock solutions of copolymer (1) and (2) used to make the protocells. 80 mg mL^{-1} black plot, 60 mg mL^{-1} yellow plot, 30 mg mL^{-1} light blue plot, and 15 mg mL^{-1} green plot, see composition in Supplementary Section S3.2.a and Table S2, Entry 2.

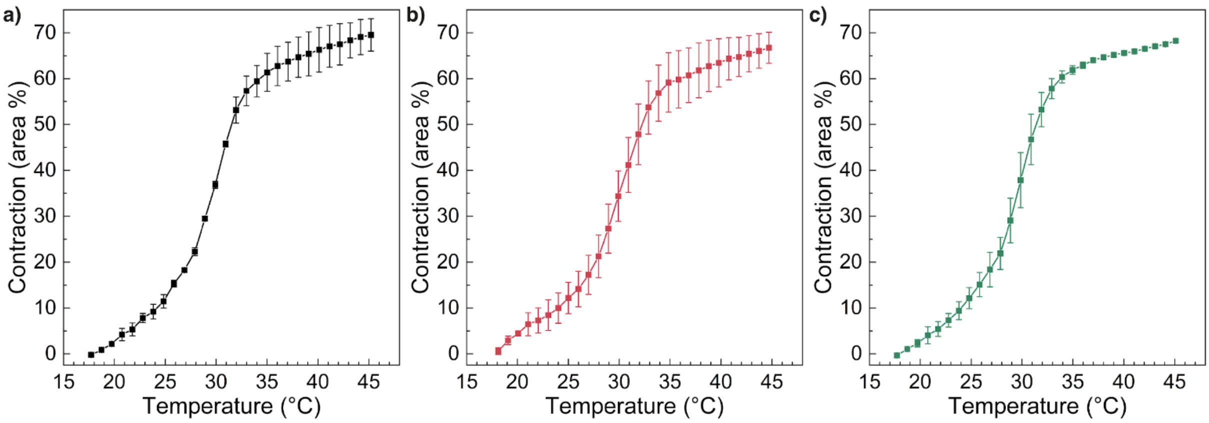

Figure S36. Plots showing the temperature dependent area contraction of prototissues with PNIPAM proto-cortex, upon varying the amount of encapsulated PEG-AuNPs. All measurements were performed on prototissues floating in Milli-Q water, laid on a thermocouple (see Supplementary Section S1.5). a) Prototissue composed by protocells endowed with PNIPAM proto-cortex (for detailed composition see Supplementary Section S3.2.a and Table S2, Entry 2). b) Prototissue structured as in (a) comprising PEG-AuNPs at a concentration of 1.33 mg mL^{-1} (for detailed composition see Supplementary Section S3.2.b, Table S1 Entry 4 and Table S2, Entry 3). c) Prototissue structured as in (a) comprising PEG-AuNPs at a concentration of 10.7 mg mL^{-1} (for detailed composition see Supplementary Section S3.2.b, Table S1 Entry 7 and Table S2, Entry 3). Error bars correspond to standard error, calculated on three measurements repeated on three freshly prepared samples.

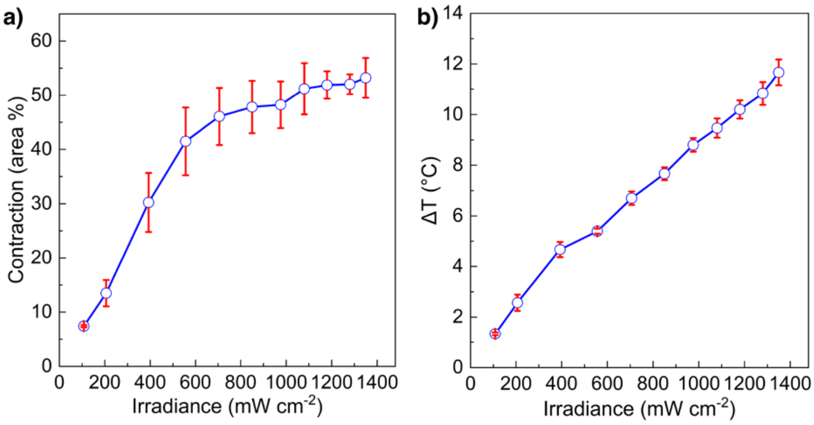

Figure S37. Photo-induced prototissue contraction and temperature variation as a function of LED irradiance. All measurements were performed on prototissues floating in Milli-Q water, laid on a thermocouple (see Supplementary Section S1.5), at 25 °C. a) Plot showing the light-induced area contraction of 5 mm diameter circular prototissues composed of proteinosomes encapsulating PNIPAM proto-cortex and PEG-AuNPs (1.33 mg mL^{-1}) as a function of the irradiance power, which was systematically varied between 100 and 1400 mW cm^{-2}. For the detailed composition of the prototissue see Supplementary Section S3.2.b, Table S1 Entry 4 and Table S2, Entry 3. b) Plot showing the changes in temperature of the prototissue in (a) measured by the thermocouple as a function of the LED irradiance. Error bars correspond to standard error, calculated on three measurements repeated on three freshly prepared samples.

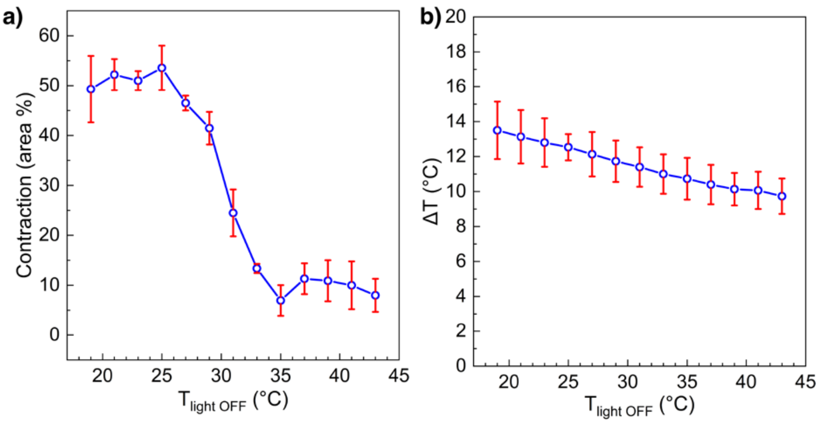

Figure S38. Photo-induced prototissue contraction and temperature variation as a function of the starting temperature. All measurements were performed on prototissues floating in Milli-Q water, laid on a thermocouple (see Supplementary Section S1.5). a) Photo-induced contraction and b) localized temperature variation after 120 s irradiation (irradiance: 1.35 W cm^{-2}) of 5 mm diameter circular prototissues composed of proteinosomes encapsulating PNIPAM proto-cortex and PEG-AuNPs 1.33 mg mL^{-1} (see composition in Supplementary Section S3.2.b, Table S1 Entry 4 and Table S2, Entry 3) upon variation of starting temperature (temperature at which light was OFF). Error bars correspond to standard error, calculated on three measurements repeated on independently prepared samples.

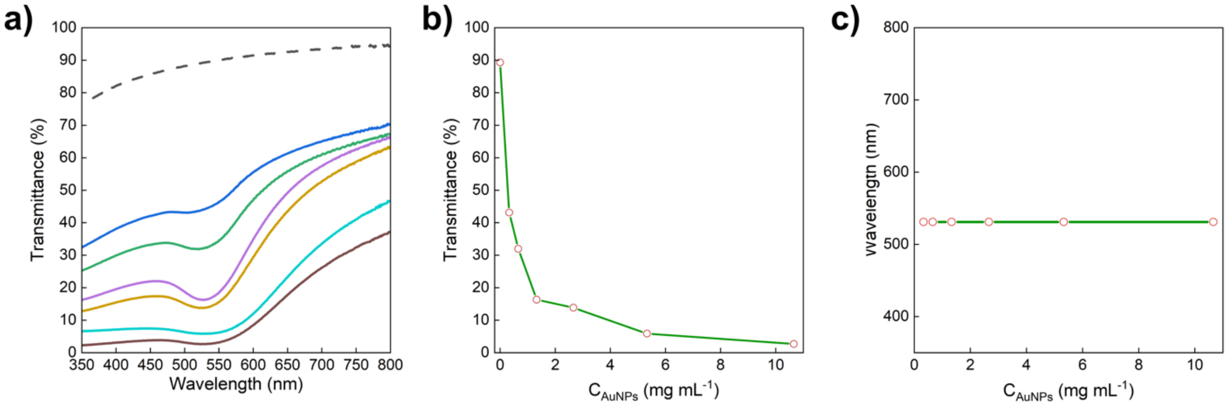

Figure S39. a) UV-vis transmittance spectra of prototissues composed of protocells with a PNIPAM-based proto-cortex, encapsulating varying concentrations of PEG-AuNPs (black dashed line: no PEG-AuNPs; blue: 0.33 mg mL^{-1}, green: 0.66 mg mL^{-1}, purple: 1.33 mg mL^{-1}, light brown: 2.66 mg mL^{-1}, cyan: 5.33 mg mL^{-1}, and brown: 10.63 mg mL^{-1}; see Supplementary Section S3.2.b and Table S2, entries 2–3 for details). b) Plot showing transmittance values at the minimum of the plasmon resonance band (from panel (a), 527 nm) plotted as a function of PEG-AuNPs concentration encapsulated in the protocells that compose the prototissues. c) Plot showing wavelength values at the minimum of the plasmon resonance band (from panel (a)) plotted as a function of PEG-AuNPs concentration encapsulated in the protocells that compose the prototissues. The plot shows that the plasmon resonance band remains constant at 527 nm, indicating no aggregation of the PEG-AuNPs.

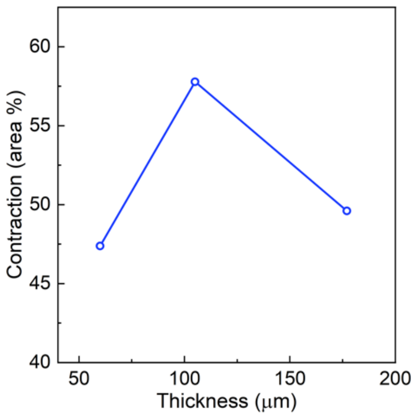

Figure S40. Photo-induced contraction as a function of the prototissue thickness. All measurements were performed on prototissues floating in Milli-Q water, laid on a thermocouple, and thermostated at 25 °C (see Supplementary Section S1.5). Prototissues were formed using increasing volumes of the 1:1 binary proteinosome emulsion per unit area of mold. Specifically, 0.2, 0.4, and 0.6 mm^{-2} were injected into the PTFE mold to assemble the prototissues. The higher the emulsion volume per unit area of the mold used, the thicker the prototissue, as showed in our previous work.[11]

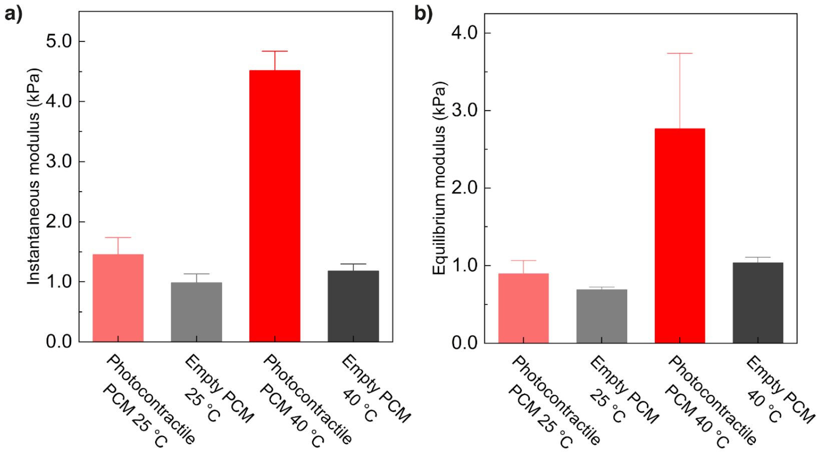

Figure S41. Histograms reporting instantaneous and equilibrium moduli obtained from force relaxation tests. Measurements were performed on prototissues containing protocells endowed with the PNIPAM proto-cortex (red) and prototissues containing empty protocells (grey) at 25 °C (light colored) and 40 °C (dark colored). a) Instantaneous moduli at 25 °C were equal to 1.5 ± 0.5 kPa and 1.0 ± 0.1 kPa for prototissues containing protocells endowed with the PNIPAM proto-cortex and prototissues containing empty protocells, respectively. At 40 °C the moduli changed to: 4.5 ± 0.3 kPa and 1.2 ± 0.1 kPa. b) Equilibrium moduli at 25 °C were 0.9 ± 0.3 kPa and 0.70 ± 0.05 kPa for prototissues containing protocells endowed with the PNIPAM proto-cortex and prototissues containing empty protocells, respectively. At 40 °C they changed to: 2.8 ± 1.0 kPa and 1.0 ± 0.1 kPa. Error bars indicate standard error calculated upon repeating the measurements on at least three independently prepared samples, on nine different areas per sample.

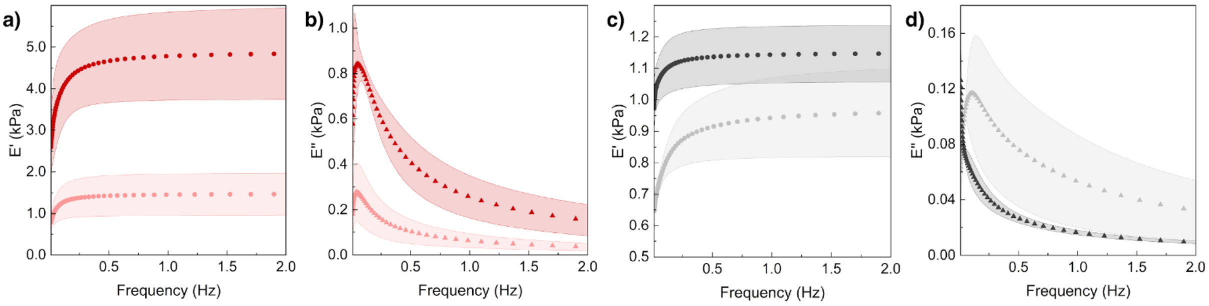

Figure S42. Plots reporting storage (*E'*) and loss (*E''*) moduli vs. frequency (ω) calculated by applying Fourier Transform to *E(t)*. Prototissues containing protocells endowed with the PNIPAM proto-cortex (red: a, b) and prototissues containing empty protocells (grey: c, d) were tested at 25 °C (light colored) and 40 °C (dark colored). Plots report mean and relative standard deviation values for storage (a, c) and loss (b, d) moduli. Colored bands indicate standard error calculated upon repeating the measurements on at least three independently prepared samples, on nine different areas per sample.

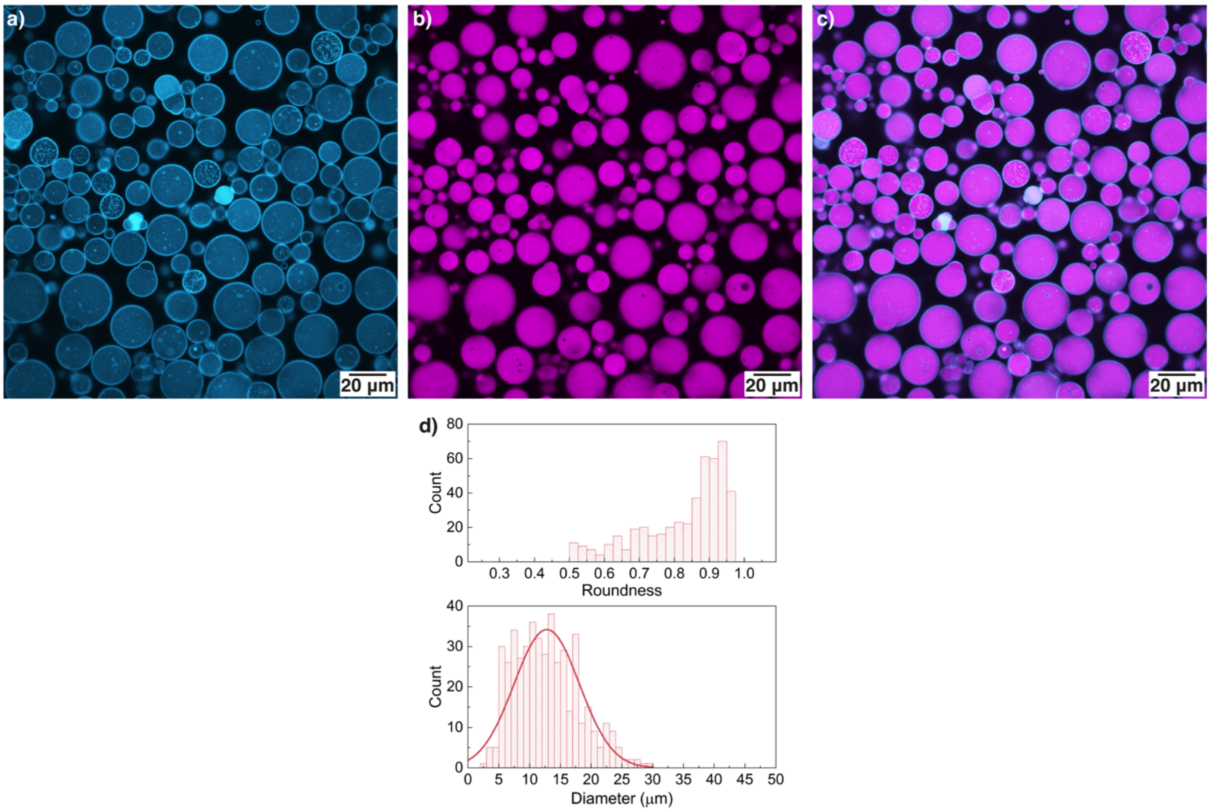

Figure S43. Confocal fluorescence micrograph showing a population of bio-orthogonally reactive proteinosomes in oil containing a polymer network composed of copolymers (3) and (4) (composition: Supplementary Table S2, Entry 6). a) Fluorescence channel corresponding to the AMCA-labeled bio-orthogonal BSA/PNIPAM-*co*-MAA nanoconjugates (blue fluorescence). b) Fluorescence channel corresponding to the polymer network formed by copolymer (3) (purple fluorescence) and copolymer (4). c) Merged image of (a) and (b). d) Distribution plots highlighting mean equivalent diameter of 13 ± 5 µm and roundness peaking at 0.9 ± 0.05, red curve indicates gaussian fitting of distribution data.

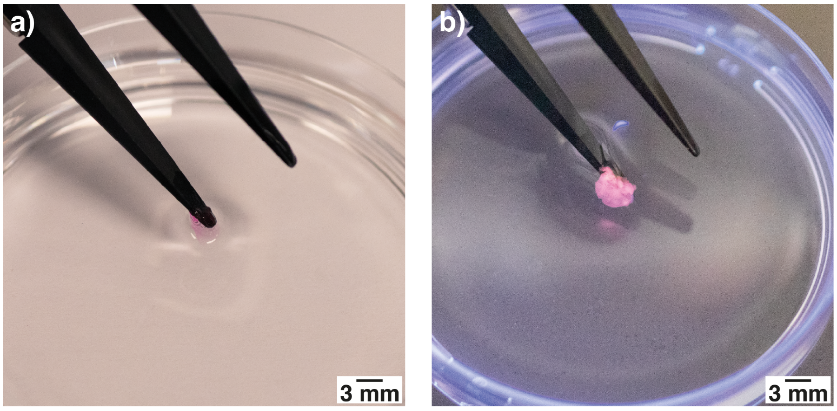

Figure S44. Pictures of PCMs with PDMAM proto-cytoskeleton (composition: Supplementary Table S2, Entry 6). Picture in (a) was taken under environmental light. Picture in (b) was taken under UV illumination.

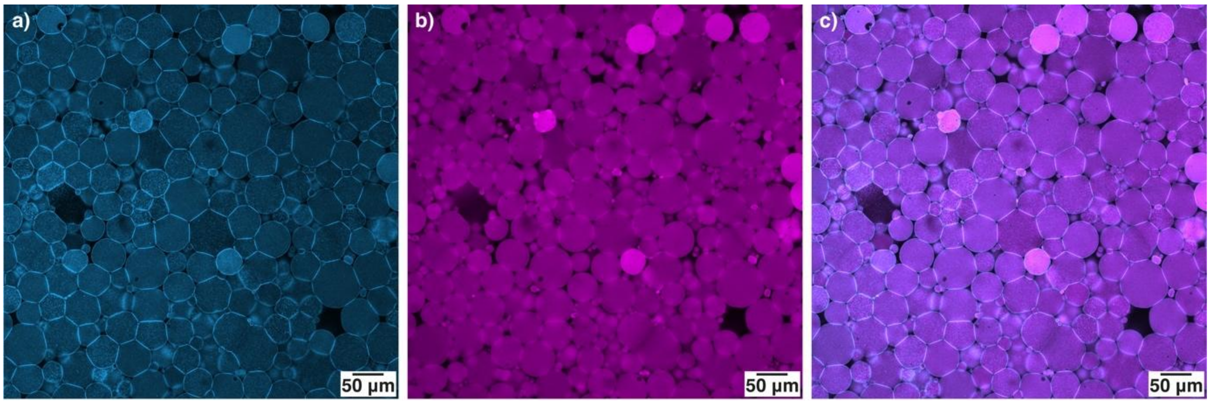

Figure S45. Confocal fluorescence microscopy image showing a prototissue composed of proteinosomes endowed with a PDMAM proto-cytoskeleton. The PCM was formed from a binary emulsion of bio-orthogonal proteinosomes each containing copolymers (3) and (4) (see composition in Supplementary Table S2, Entry 6). a) Fluorescence channel corresponding to the AMCA-labeled BCN- and azide-functionalized BSA/PNIPAM-*co*-MAA nanoconjugates (blue fluorescence). b) Fluorescence channel corresponding to the PDMAM polymer network formed by copolymers (3) (purple fluorescence) and (4). c) Merged image of (a) and (b).

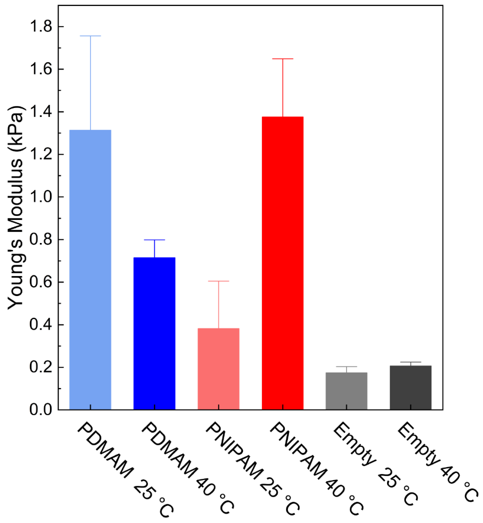

Figure S46. Histograms reporting Young's modulus values obtained from compression tests. Prototissues composed of protocells endowed with the PDMAM (blue) proto-cytoskeleton and the PNIPAM (red) proto-cortex, and prototissues composed of empty protocells (grey) were tested at 25 °C (light colored) and 40 °C (dark colored). Young's moduli mean values and standard deviation at 25 °C are: 1.3 ± 0.5 kPa, 0.4 ± 0.2 kPa and 0.17 ± 0.04 kPa for prototissues comprising protocells endowed with the PDMAM proto-cytoskeleton, PNIPAM proto-cortex, and prototissues composed of empty protocells, respectively. At 40 °C Young's moduli shift to: 0.7 ± 0.1 kPa, 1.4 ± 0.2 kPa and 0.21 ± 0.02 kPa.

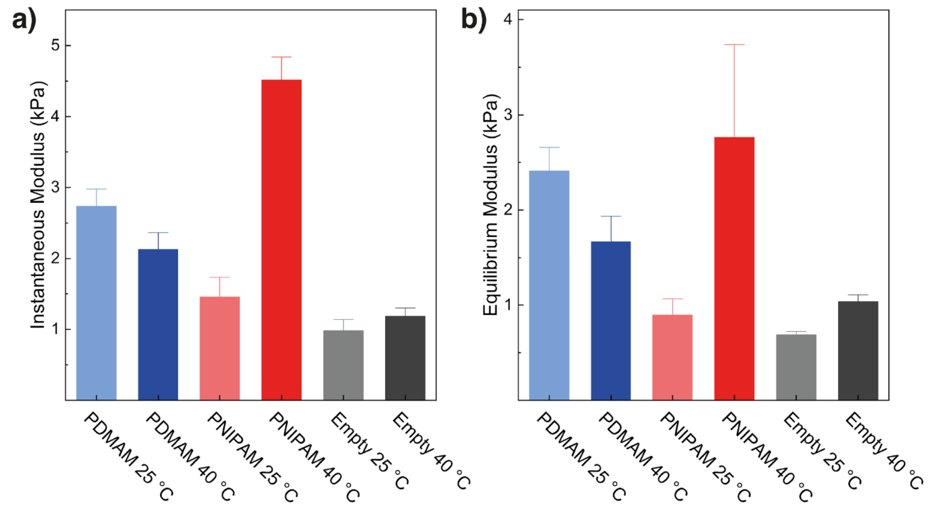

Figure S47. Histograms reporting the comparison between instantaneous and equilibrium moduli obtained from force relaxation tests. Measurements were performed on prototissues containing protocells endowed with the PDMAM (blue) proto-cytoskeleton and the PNIPAM (red) proto-cortex, and prototissues containing empty protocells (grey) at 25 °C (light colored) and 40 °C (dark colored). a) Instantaneous moduli at 25 °C were equal to 2.7 ± 0.3 kPa, 1.5 ± 0.5 kPa, 1.0 ± 0.1 kPa for prototissues composed of protocells endowed with the PDMAM proto-cytoskeleton, PNIPAM proto-cortex, and empty protocells, respectively. At 40 °C they changed to: 2.1 ± 0.3 kPa, 4.5 ± 0.3 kPa, 1.2 ± 0.1 kPa. b) Equilibrium moduli at 25 °C were 2.4 ± 0.3 kPa, 0.9 ± 0.3 kPa and 0.70 ± 0.05 kPa for prototissues composed of protocells endowed with the PDMAM proto-cytoskeleton, PNIPAM proto-cortex, and empty protocells, respectively. At 40 °C they changed to: 1.7 ± 0.2 kPa, 2.8 ± 1.0 kPa, 1.0 ± 0.1 kPa.

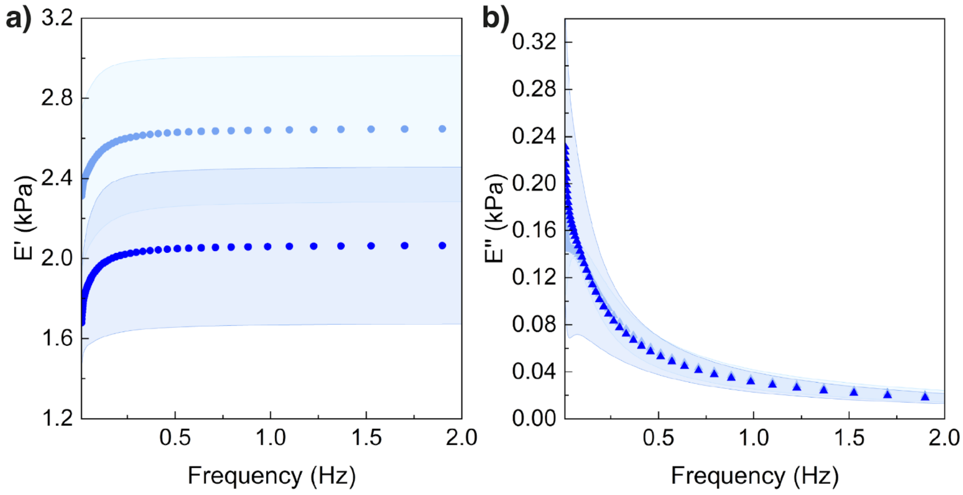

Figure S48. Plots reporting storage (*E'*) and loss (*E''*) moduli *vs.* frequency (ω) calculated by applying Fourier Transform to *E(t)*. Prototissues composed of protocells endowed with the PDMAM proto-cytoskeleton were tested at 25 °C (light colored) and 40 °C (dark colored). The plots report mean and relative standard deviation values for storage (a) and loss (b) moduli. Colored bands indicate standard error calculated upon repeating the measurements on at least three independently prepared samples, on nine different areas per sample.

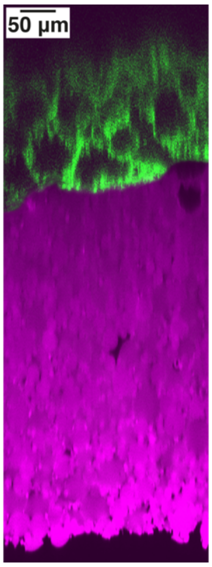

Figure S49. PCM bilayer thickness estimation. Representative XZ orthogonal plane of a confocal fluorescence Z-stack obtained by imaging the starfish-shaped bilayer PCM in Figure 3b-f throughout its whole thickness. Fluorescence channels corresponding to the proteinosomes encapsulating PEG-AuNPs and FITC-labeled PNIPAM (green fluorescence) and RITC-labeled PDMAM (purple fluorescence). PNIPAM layer thickness was estimated as 176 ± 9 µm, PDMAM layer thickness was estimated as 480 ± 30 µm. Thickness was determined manually using image analysis software (ImageJ) over 2 images taken in different PCM areas, 6 XZ planes each, 3 measurements on each XZ plane. Averaged data, error: standard deviation.

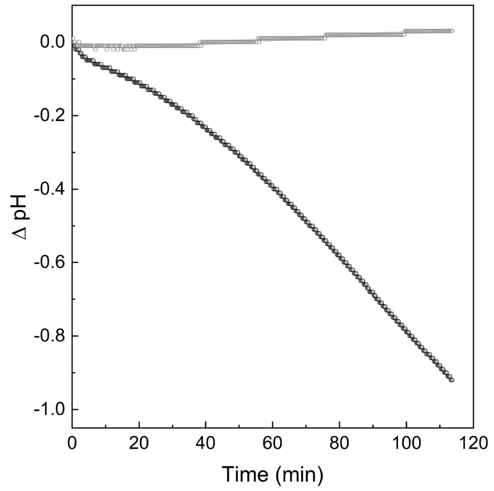

Figure S50. Negative control experiment performed as in **Figure 4d**, with a PCM encapsulating only GOx (no AGx) – grey points, as opposed to a PCM encapsulating both AGx and GOx – black points.

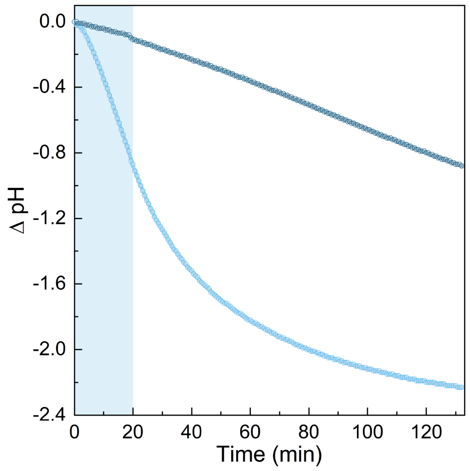

Figure S51. Negative control experiment performed as in **Figure 4d**, with AGx and GOx enzymes dissolved in the bulk solution (no PCM) – light blue points, same experiment without AGx – dark blue points. Light blue area represents 20 min of light irradiation (λ_{max} = 520 nm, irradiance: 1.35 W cm^{-2}).

Table S3. Table summarizing the characterization of the molecular weight and molecular weight distribution of all the FITC-dextran polymers employed to determine the MWCO of the prototissues. The characterization was carried out via triple detector size exclusion chromatography, experimental conditions are reported below the table.

FITC-dextran Nominal MW x 10^{-3} (g mol^{-1})	Retention volume (mL)[a]	M_n x 10^{-3} (g mol^{-1})	M_w x 10^{-3} (g mol^{-1})	M_w/M_n
4	16.29 ± 0.01	2.9 ± 0.1	4.1 ± 0.1	1.38 ± 0.03
10	15.36 ± 0.03	8.8 ± 0.1	12.47 ± 0.06	1.41 ± 0.01
20	14.56 ± 0.02	14.8 ± 0.1	20.30 ± 0.06	1.37 ± 0.01
40	14.31 ± 0.01	27.2 ± 0.2	39.45 ± 0.05	1.45 ± 0.01
70	14.09 ± 0.03	63.12 ± 0.01	75.79 ± 0.02	1.21 ± 0.01
150	14.31 ± 0.02	160 ± 3	213 ± 2	1.31± 0.04

[a]Measurement conditions: 1.5 mg mL^{-1} concentration, 100 µL injection volume, A6000+A2500 columns, 0.1 M PBS : MeOH = 9.5 : 0.5 mobile phase, 0.7 mL min^{-1} flow rate. Value calculated by the average of three separate measurements, error is expressed as the standard deviation.

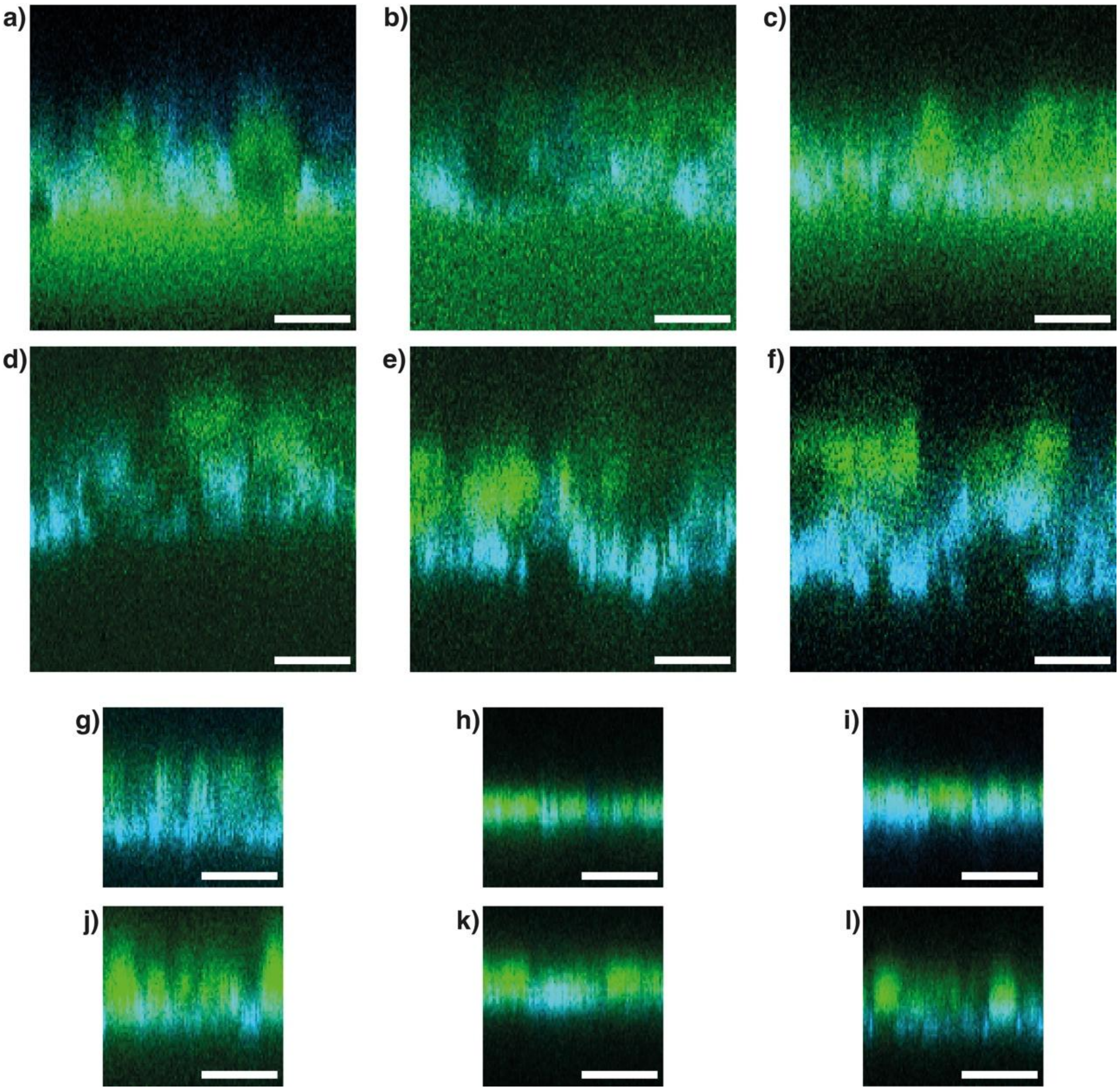

Figure S52. Characterization of the molecular weight cut-off (MWCO) of prototissues in the *relaxed* state and in the *contracted* state. The images show XZ orthogonal projections of Z-stacks obtained by confocal fluorescence microscopy of AMCA-labeled (blue channel) contractile PCMs at 25 °C (relaxed state, (a–f)), and at 40 °C (contracted state, (g–l)). In these experiments, 2 µL of an aqueous solution of FITC-labeled dextran in water (1 mg mL^{-1}, green channel) with different molecular weight (4 kDa (a, g), 10 kDa (b, h), 20 kDa (c, i), 40 kDa (d, j), 70 kDa (e, k) and 150 kDa (f, l)) was placed on top of the PCM, and its diffusion through the PCM was monitored. The images clearly show that at 25 °C (relaxed state), the PCM membrane was permeable to the FITC-dextrans with a molecular weight lower than 40 kDa. On the contrary, at 40 °C (contracted state) the PCM membrane was impermeable to all the solutes. Scale bars: 100 µm.

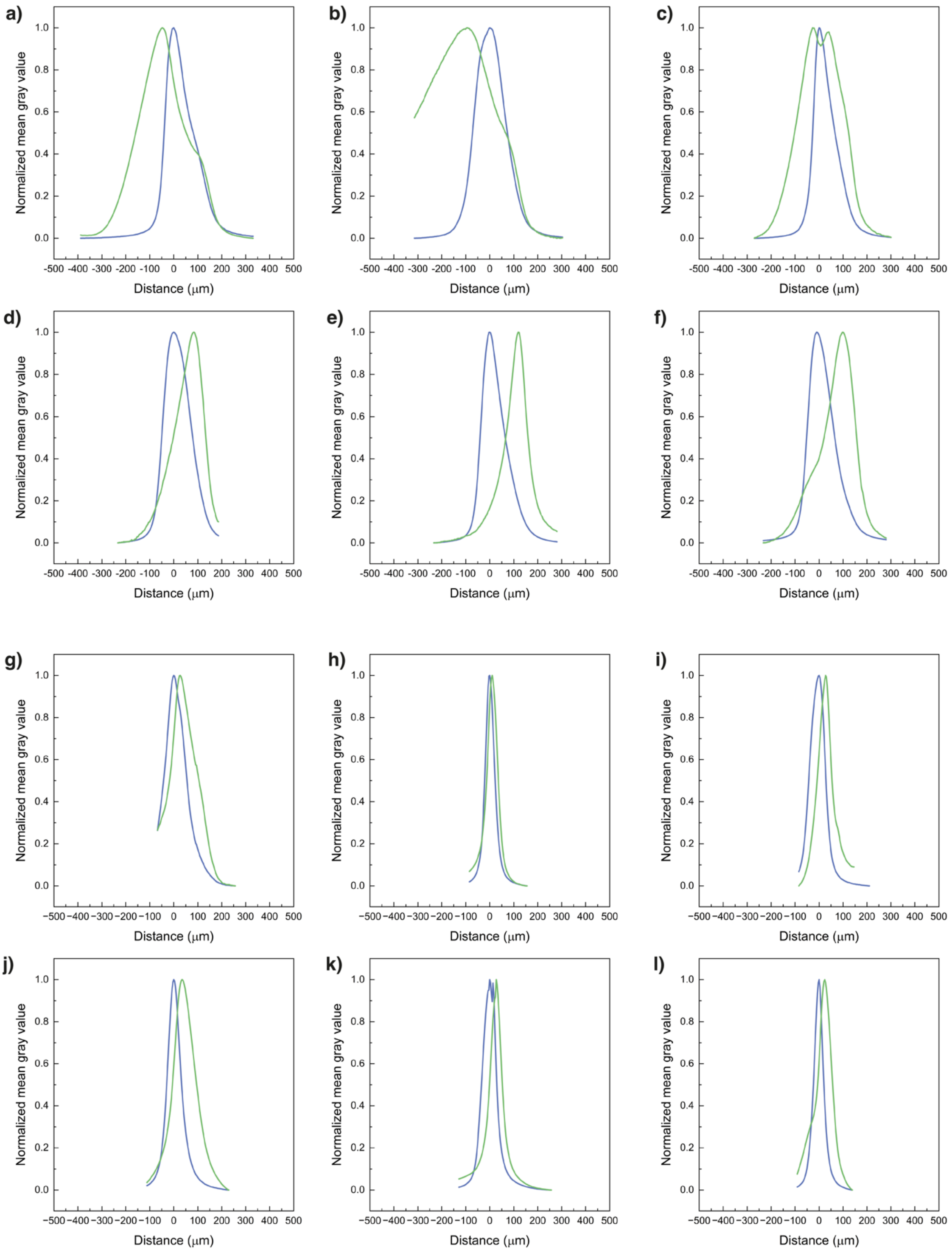

Figure S53. Molecular weight cut-off (MWCO) characterization of prototissues in the relaxed state and in the contracted state. All plots report the fluorescence intensity profiles along the XZ orthogonal axis calculated from the Z-stacks reported in Supplementary Figure S52. for the signals of AMCA-labeled contractile prototissues (blue curves) and FITC-labeled dextrans (green curves) at different temperatures (25 °C = *relaxed state*, (a–f)); 40 °C = *contracted state*, (g–l)), and for dextrans of different molecular weights: 4 kDa (a, g), 10 kDa (b, h), 20 kDa (c, i), 40 kDa (d, j), 70 kDa (e, k) and 150 kDa (f, l). The plots clearly show that at 25 °C (relaxed state), the prototissue membrane was permeable to the FITC-dextrans with a molecular weight lower than 40 kDa, as the maximum of the green curve was on the right of the maximum of the blue curve (a-c). In contrast, the prototissue was impermeable to all the solutes when the maximum of the green curve was located on the right of the maximum of the blue curves (d-l).

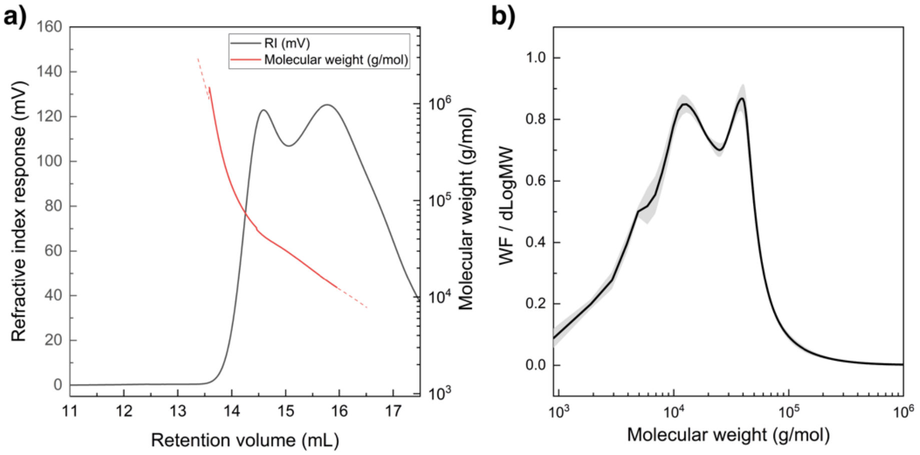

Figure S54. Molecular weight characterization of the dextrin used in this work, which was carried out by triple detector size exclusion chromatography. a) Plot showing an overlay of the refractive index chromatogram (black plot) and of the molecular weight (red plot) against the polymer retention volume. The plot shows that under the RI polymer peak the molecular weight of the dextrin diminishes with the retention volume, which is typical of a highly polydisperse polysaccharide. b) Plot showing the differential weight fraction against the dextrin molecular weight. Values calculated by the average of three separate measurements (black line), error is expressed as the standard deviation (grey band). The plot shows that there are at least two main dextrin distributions, the largest at 13,000 g mol^{-1}, and the other at 39,000 g mol^{-1}.

Table S4. Table summarizing the molecular weight and molecular weight distribution characterization of the dextrin used in this work. The characterization was carried out via triple detector size exclusion chromatography, experimental conditions are reported below the table.

Retention volume (mL)[a]	M_n x 10^{-3} ($gmol^{-1}$)[a]	M_w x 10^{-3} (g mol^{-1})[a]	M_w/M_n[a]	$MW_{component\ 1}$ x 10^{-3} (g mol^{-1})[b]	$MW_{component\ 2}$ x 10^{-3} (g mol^{-1})[b]
15.79 ± 0.01	11.7 ± 0.4	26.1 ± 0.1	2.23 ± 0.08	13	39

[a]Measurement conditions: 1.5 mg mL^{-1} concentration, 150 µL injection volume, A6000+A2500 columns, 0.1 M PBS : MeOH = 9.5 : 0.5 mobile phase, 0.7 mL min^{-1} flow rate. Value calculated by the average of three separate measurements, error is expressed as the standard deviation. [b]Determined as the local maxima of the differential fraction vs. molecular weight plot.

S8. Supplementary videos

Supplementary Video 1. Time lapse of the reversible photo-contractions of the prototissue shown in Figure 2 (irradiation: λ_{max} = 520 nm, Irr: 1.35 W cm^{-2}). The video is shown at 10x real time speed.

Supplementary Video 2. Time lapse of the bending/relaxation behavior of the six-armed starfish prototissue shown in Figure 3 (irradiation: λ_{max} = 520 nm, Irr: 1.35 W cm^{-2}, see Supplementary Section S1.6). The video is shown at 10x real time speed.

S9. References

(1) Symons, H. E.; Galanti, A.; Surmon, J. C.; Trask, R. S.; Rochat, S.; Gobbo, P. Automated analysis of soft material microindentation. *Soft Matter* **2022**, *18* (43), 8302-8314. DOI: 10.1039/d2sm00857b From NLM PubMed-not-MEDLINE.

(2) Park, J. H.; Grimes, P. J.; Symons, H. E.; Braidotti, N.; Rochat, S.; Workentin, M. S.; Gobbo, P. Photochemical Patterning and Characterization of Mechanical Properties on Soft Materials. *Adv. Funct. Mat.* **2024**. DOI: 10.1002/adfm.202416095.

(3) Efremov, Y. M.; Okajima, T.; Raman, A. Measuring viscoelasticity of soft biological samples using atomic force microscopy. *Soft Matter* **2020**, *16* (1), 64-81. DOI: 10.1039/c9sm01020c From NLM Medline.

(4) Islam, M. R.; Virag, J.; Oyen, M. L. Micromechanical poroelastic and viscoelastic properties of ex-vivo soft tissues. *J. Biomech.* **2020**, *113*, 110090. DOI: 10.1016/j.jbiomech.2020.110090 From NLM Medline.

(5) Kraus, M. A.; Schuster, M.; Kuntsche, J.; Siebert, G.; Schneider, J. Parameter identification methods for visco- and hyperelastic material models. *Glass Structures & Engineering* **2017**, *2* (2), 147-167. DOI: 10.1007/s40940-017-0042-9.

(6) Gobbo, P.; Patil, A. J.; Li, M.; Harniman, R.; Briscoe, W. H.; Mann, S. Programmed assembly of synthetic protocells into thermoresponsive prototissues. *Nat. Mater.* **2018**, *17* (12), 1145-1153. DOI: 10.1038/s41563-018-0183-5.

(7) Kusolkamabot, K.; Sae-ung, P.; Niamnont, N.; Wongravee, K.; Sukwattanasinitt, M.; Hoven, V. P. Poly(N-isopropylacrylamide)-stabilized gold nanoparticles in combination with tricationic branched phenylene-ethynylene fluorophore for protein identification. *Langmuir* **2013**, *29* (39), 12317-12327. DOI: 10.1021/la402139g From NLM Medline.

(8) Scarabelli, L.; Sanchez-Iglesias, A.; Perez-Juste, J.; Liz-Marzan, L. M. A "Tips and Tricks" Practical Guide to the Synthesis of Gold Nanorods. *J. Phys. Chem. Lett.* **2015**, *6* (21), 4270-4279. DOI: 10.1021/acs.jpclett.5b02123.

(9) Gobbo, P.; Workentin, M. S. Improved methodology for the preparation of water-soluble maleimide-functionalized small gold nanoparticles. *Langmuir* **2012**, *28* (33), 12357-12363. DOI: 10.1021/la302168g From NLM Medline.

(10) Gobbo, P.; Luo, W.; Cho, S. J.; Wang, X.; Biesinger, M. C.; Hudson, R. H.; Workentin, M. S. Small gold nanoparticles for interfacial Staudinger-Bertozzi ligation. *Org. Biomol. Chem.* **2015**, *13* (15), 4605-4612. DOI: 10.1039/c5ob00372e From NLM Medline.

(11) Galanti, A.; Moreno-Tortolero, R. O.; Azad, R.; Cross, S.; Davis, S.; Gobbo, P. A Floating Mold Technique for the Programmed Assembly of Protocells into Protocellular Materials Capable of Non-Equilibrium Biochemical Sensing. *Adv. Mater.* **2021**, *33* (24), e2100340. DOI: 10.1002/adma.202100340.

(12) Takigawa, T.; Yamawaki, T.; Takahashi, K.; Masuda, T. Change in Young's modulus of poly(N-isopropylacrylamide) gels by volume phase transition. *Polymer Gels and Networks* **1998**, *5* (6), 585-589. DOI: 10.1016/s0966-7822(97)00028-2.

(13) Matzelle, T. R.; Geuskens, G.; Kruse, N. Elastic Properties of Poly(N-isopropylacrylamide) and Poly(acrylamide) Hydrogels Studied by Scanning Force Microscopy. *Macromolecules* **2003**, *36* (8), 2926-2931. DOI: 10.1021/ma021719p.

(14) Sakai, T.; Katashima, T.; Matsushita, T.; Chung, U.-i. Sol-gel transition behavior near critical concentration and connectivity. *Polymer Journal* **2016**, *48* (5), 629-634. DOI: 10.1038/pj.2015.124.
